# The role of gut microbiome in the pathophysiology of PTSD, depression, and anxiety disorders

**DOI:** 10.1080/29933935.2026.2654224

**Published:** 2026-04-14

**Authors:** Nto Johnson Nto, Walter Pirovano, Kristien Nel Van Zyl, Stefanie Malan-Müller, Emma Koch, Matsepo Ramaboli, Dineo Madikgetla, Christiaan de Leeuw, Christopher A. Lowry, Sian Megan Joanna Hemmings

**Affiliations:** a Department of Psychiatry, Faculty of Medicine and Health Sciences, Stellenbosch University, Cape Town, South Africa; b Department of Psychiatry, SU/SAMRC Genomics of Brain Disorders Extramural Research Unit, Faculty of Medicine and Health Sciences, Stellenbosch University, Cape Town, South Africa; c Department of Anatomy, Faculty of Basic Medical Sciences, University of Nigeria, Enugu, Nigeria; d Department of Complex Trait Genetics, Center for Neurogenomics and Cognitive Research (CNCR), Vrije Universiteit Amsterdam, Amsterdam, The Netherlands; e Department of Biomedical Sciences, African Microbiome Institute, Division of Molecular Biology and Human Genetics, Faculty of Medicine and Health Sciences, Stellenbosch University, Cape Town, South Africa; f Department of Pharmacology and Toxicology, Faculty of Medicine, University Complutense Madrid (UCM), Madrid, Spain; g Department of Pharmacology and Toxicology, Neuropsychopharmacology Group, Biomedical Network Research Centre of Mental Health (CIBERSAM), Institute of Health Carlos III, Madrid, Spain; h Department of Pharmacology and Toxicology, University Complutense of Madrid (UCM), Research Institute of Hospital 12 de Octubre (Imas12), Instituto Universitario de Investigación Neuroquímica, Madrid, Spain; i Faculty of Biosciences, Heidelberg University, Heidelberg, Germany; j Department of Integrative Physiology, Center for Neuroscience, and Center for Microbial Exploration, University of Colorado Boulder, Boulder, CO, USA

**Keywords:** Gut–brain axis, stress-related disorders, dysbiosis, microbiome biomarkers, host–microbe interactions

## Abstract

Posttraumatic stress disorder (PTSD), depression, and anxiety disorders are prevalent and often overlapping mental health conditions with complex, multifactorial etiologies. Growing evidence implicates the gut microbiome in their pathophysiology through immune modulation, neurotransmitter regulation, and bidirectional gut–brain signaling. Findings remain fragmented and difficult to reconcile due to differences in study populations, clinical contexts, and analytic methods. This structured narrative review synthesizes current evidence on gut microbial alterations in PTSD, depression, and anxiety, while examining methodological sources of heterogeneity. We searched four databases: PubMed, Scopus, Web of Science, and PsycINFO, and identified 64 eligible studies assessing the gut microbiome composition in these disorders. Sample sizes ranged from small, exploratory cohorts (≈20 participants) to large population-based datasets (>1000 participants), with most studies conducted in China. Stool sampling and DNA extraction protocols varied widely, although 16S rRNA gene amplicon sequencing of the V3–V4 region on Illumina platforms predominated. Alpha-diversity results were inconsistent, whereas beta-diversity analyses distinguished cases from controls. Across these disorders, alterations in microbial composition was observed, including enrichment of proinflammatory and depletion of beneficial bacterial taxa. The current findings indicate that that the gut microbiome represents a promising avenue for biomarker discovery and therapeutic innovation.

## Introduction

Posttraumatic stress disorder (PTSD), depression, and anxiety disorders are among the most common psychiatric disorders worldwide.^1^^,^^2^ They affect quality of life and pose immense societal and economic burdens.^3^ The global lifetime prevalence of PTSD is 3.9%,^4^^,^^5^ while depression and anxiety disorders affect 4.4%^6^ and 3.7%^7^ of the world's population, respectively. Despite scientific advances, the diagnosis of these disorders relies primarily on symptom-based or clinician-dependent assessments, with objective biomarkers for prediction and monitoring still lacking. The lack of clinical biomarkers may result in misdiagnosis and consequently ineffective treatments,^8^ necessitating new insights into the pathophysiology of psychiatric disorders to enhance diagnosis and therapy. Over the past decade, growing scientific interest has focused on the gut microbiome's role in the pathophysiology of PTSD, depression, and anxiety disorders.^9^ Emerging evidence suggests that alterations in gut microbial diversity and composition are linked to the onset, progression, and severity of these conditions.^10-12^

The gut microbiome refers to the diverse community of microorganisms inhabiting the gastrointestinal tract. Beyond its roles in digestion, nutrient absorption, and immune regulation,^13^ the gut microbiome helps maintain intestinal barrier integrity, controlling the flow of substances between the gut and the bloodstream.^14^ Disruptions in this balance can lead to inflammation through bacterial translocation and increased gut permeability.^15^^,^^16^ These physiological disruptions are thought to influence mental health *via* the gut‒brain axis, a bidirectional communication network linking the gut and central nervous system.^10^ This axis integrates neural, endocrine, immune, and metabolic pathways to regulate stress responses, mood, and cognition.^10^^,^^13^^,^^17^

Preclinical studies have been instrumental in elucidating gut‒brain connections.^18^ Germ-free mice,^19-22^ fecal microbiota transplants,^22-24^ and microbiota-targeted interventions^20^^,^^25^ in animal models have demonstrated the influence of gut microbes on behavior and brain function.^19^ Findings from these studies represent important landmarks for precision medicine, positioning the gut microbiome as potential therapeutic target. However, the findings on microbial diversity and taxonomic abundance have been inconsistent between studies.^26^ While some authors have reported distinct differences in microbial composition between cases and controls, others have failed to replicate these findings. Similarly, alterations in microbial diversity between patients and controls have been observed in some studies but not in others. These inconsistent findings can be attributed to, among others, differences in experimental design and technologies, the choice of analysis frameworks and reference databases.^27^ In the context of depression, one of the most commonly investigated markers is the Firmicutes:Bacteroidetes (F:B) ratio (now referred to as the Bacillota:Bacteriodota ratio), which typically decreases in depressed individuals.^28^^,^^29^ That said, the results at lower taxonomic levels (i.e. genus or species) are considerably more variable and inconsistent than those at the phylum level.

Existing reviews have examined gut microbiome roles in PTSD, depression, and anxiety disorders,^4^^,^^30-35^ addressing individual disorders^4^^,^^30-35^ as well as therapeutic potential and gut‒brain axis interactions.^36-38^ While these reviews have provided preliminary knowledge on the role of the gut microbiome in these disorders, much of the literature remains largely descriptive and exploratory, with limited integration of mechanistic evidence. Moreover, none of the existing reviews have systematically evaluated methodological differences, such as sample collection, DNA extraction, sequencing technologies, and data analysis, as potential sources of inconsistent findings. To address these gaps, we reviewed and synthesized current evidence on the role of the gut microbiome in PTSD, depression, and anxiety disorders, focusing on human research. Our objectives were to (1) identify microbial taxa commonly associated with these disorders and integrate these findings with emerging mechanistic evidence to generate more robust hypotheses regarding functional shifts underlying disease processes and (2) evaluate study design features, including sample collection methods, sequencing platforms, and analytical approaches that introduce potential inconsistencies across studies. In addition, we outline best-practice recommendations that may strengthen methodological rigor to increase reproducibility and advance the integration of microbiome science into mental health research.

## Methods and materials

### Data sources and search strategy

We conducted a structured narrative review to examine the role of the gut microbiome in the pathophysiology of PTSD, depression, and anxiety disorders. We searched four databases: PubMed, Scopus, Web of Science, and PsycINFO. Our literature search was designed to identify publications reporting on gut microbiome, PTSD, depression and anxiety disorders in humans, and comprised the following terms “*gastrointestinal microbiome*” OR “*gut microbiome*” OR “*intestinal microbiota*” OR “*gut-brain axis*” OR “*microbial diversity*” AND “*mental disorders*” OR “*psychiatric disorders*” OR “*mental health*” OR “*post-traumatic stress disorder*” OR “*PTSD*” OR “*depression*” OR “*depressive disorders*” OR “*anxiety*” OR “*anxiety disorders*” OR “*stress-related disorders*”.

### Inclusion and exclusion criteria

We included studies that met the following criteria: (1) peer-reviewed human studies published in English; (2) studies involving individuals diagnosed with PTSD, depression, and/or anxiety disorders (either clinically diagnosed or self-reported), with healthy controls as the comparison group; (3) studies that assessed gut microbiome composition in relation to these mental health conditions; and (4) case-control (cross-sectional or longitudinal) or interventional (clinical trials).

We excluded: (1) studies for which the full text publication was not available and (2) studies involving participants with PTSD, depression, and/or anxiety disorders complicated inflammatory bowel disease (IBD), liver failure, or autoimmune disorders, due to the established mucosal inflammation and immune dysregulation inherent to these conditions, which may independently alter the gut microbiome. Studies involving participants with irritable bowel syndrome (IBS), cirrhosis, polycystic ovary syndrome (PCOS), or rectal cancer were included only when psychiatric outcomes were assessed independently of the comorbid condition, thereby permitting meaningful interpretation of gut–brain interactions relevant to PTSD, depression, and anxiety.

### Study selection and data extraction

Output obtained from the four databases were uploaded to Rayyan^39^ to facilitate the removal of duplicates and the preliminary screening of abstracts. Data extracted included (1) study and population characteristics (study design, sample size, age range, sex, ancestry, and diagnostic criteria); (2) experimental methods (sample type, method of sample collection, extraction method, 16S rRNA gene amplicon analysis, and shotgun metagenomics analysis); and (3) gut microbiota results (alpha diversity, beta diversity, and relative abundances of specific taxa).

## Results

### Search results

**Figure 1.**
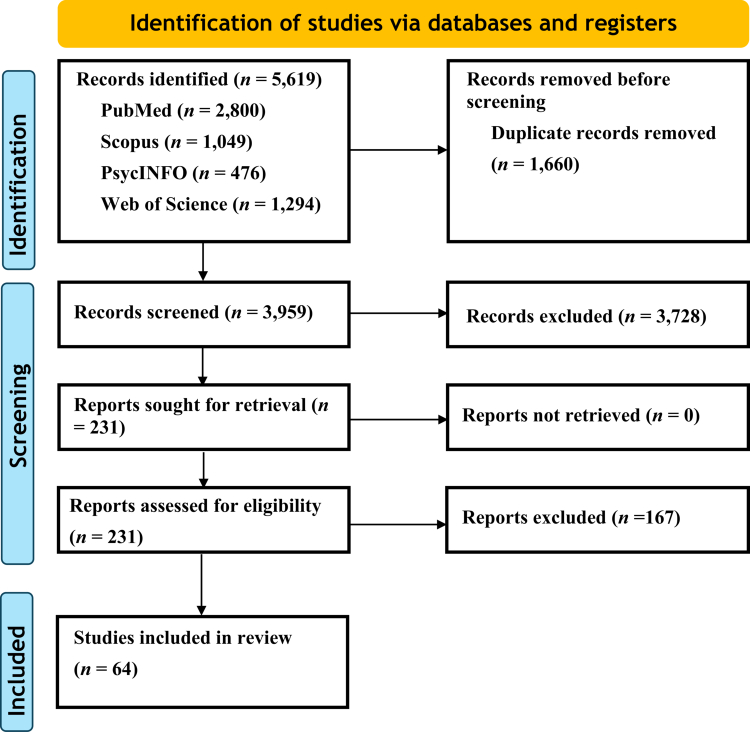
Flowchart showing the progression of information through the stages of the review.

We identified a total of 5619 records (PubMed [*n* = 2800], Scopus [*n* = 1049], PsycINFO [*n* = 476], and Web of Science [*n* = 1294]) (Figure 1). Sixty-four studies published between 2014 and 7 May 2024, were included in the final review.

### Study characteristics and geographic distribution

The characteristics of the 64 studies (Figure 1) included in this review are shown in Table 1 and Supplementary Table 1. The sample sizes of individual studies ranged from small cohorts (*n* = 20) to a large cohort (*n* = 4610). Fifty-three studies (83%) were cross-sectional, seven (11%) were longitudinal, and four (6%) were intervention-based. Sixty studies (93.75%) were case‒control studies. All were population-based studies except Yirmiya et al.^40^, in which mother‒child dyads were assessed.

**Table 1.** Study characteristics of included articles.

Study ID	Study design	Sample size	Country/ancestry	Diagnostic criteria	Diagnosis	Comorbidities
Aizawa et al. [41]	Case‒control, cross-sectional study	100	Japan/Japanese	DSM-IV, ROME III criteria	Clinically diagnosed	Not specified
Bai et al. [42]	Case‒control, cross-sectional study	112	China/not specified	DSM-IV-TR	Clinically diagnosed	Not specified
Bajaj et al. [43]	Case‒control, cross-sectional, observational study	93	USA/not specified	DSM-5	Clinically diagnosed	Cirrhosis with and without prior OHE
Bosch et al. [44]	Cross-sectional study	3211	Dutch, South-Asian Surinamese, African Surinamese, Ghanaian, Turkish, Moroccan	PHQ-9	Self-reported	Not specified
Busch et al. [45]	Case‒control, cross-sectional study	78	Germany/Deutsche	DSM-5; MINI	Clinically diagnosed	Not specified
Caso et al. [46]	Case‒control, cross-sectional study	113	Spain/Spanish (White Caucasian)	DSM-IV-TR Rome III criteria	Clinical diagnosis	Not specified
Chen et al. [47]	Case‒control study, cross-sectional survey	20;	China/not specified	DSM-IV; HAMD-17	Clinically diagnosed	Stress symptoms
Chen et al. [48]	Case‒control study, cross-sectional survey	48;	China/Han Chinese	HDRS-17	Clinically diagnosed	Not specified
Chen et al. [49]	Case‒control study, cross-sectional survey	60	China/Han Chinese	DSM-5; HAMA; SAS	Clinically diagnosed	Not specified
Chen et al. [50]	Case‒control study, cross-sectional survey	141	China/Han Chinese	DSM-IV; HAMD-17	Clinically diagnosed	Not specified
Chen et al. [51]	Case‒control study, cross-sectional survey	108	China/Han Chinese	DSM-5; MINI; HAMD-17; HAMA; PNSS, GAF	Clinically diagnosed	Anxiety symptoms
Chen et al. [52]	Case‒control study, cross-sectional survey	20	Taiwan/Taiwanese	DSM-IV; BAI; BDI; PSS	Clinically diagnosed	Anxiety symptoms
Fatt et al. [53]	Cross-sectional study	179	USA/Caucasian 133 (74.3%), Black/African American 26 (14.5%), others 13 (7.2) and unknown 7 (3.9) Hispanics in the study were 21 (11.7%)	PHQ-9, GAD-7	Self-reported	Anxiety symptoms
Chung et al. [54]	Case‒control study, cross-sectional survey	73	Taiwan/Taiwanese	DSM-5, HAMD-17, BDI, BAI, PSS, SAD-L, FFQ	Clinically diagnosed	Anxiety symptoms
Dong et al. [55]	Case‒control study, comparative, cross-sectional survey	54	China/Han Chinese	DSM-5, HAMD-24, HAMA	Clinically diagnosed	Anxiety symptoms
Dong et al. [56]	Case‒control, cross-sectional, observational study	87	China/Han Chinese	DSM-IV, HAMD-17, HAMA	Clinically diagnosed	Anxiety symptoms
Ganci et al. [57]	Cross-sectional, observational study	4610	Australia/not specified	Not Specified	Self-reported	Stress, anxiety, insomnia and fatigue symptoms
Gao et al. [58]	Longitudinal cohort study	175	China/Han Chinese	PHQ-9, GAD, PSQI, IES-R, PHQ-15	Clinically diagnosed	
Gao et al. [59]	Case‒control, longitudinal cohort study	92	China/Han Chinese	DSM-IV, HAMD-17	Clinically diagnosed	Not specified
Gonzalez-Mercado et al. [60]	Cross-sectional pilot study	40	USA/Not Specified	HAMD-17	Clinically diagnosed	Rectal cancer
Guo et al. [61]	Case‒control study, cross-sectional survey	92	China/Chinese	DSM-IV, HAMD-17	Clinically diagnosed	
Hemmings et al. [62]	Case‒control, exploratory cross-sectional study	30	South African/mixed ancestry	DSM-5 CAPS-5	Clinically diagnosed	MDD, anxiety disorders
Hope et al. [63]	Case control, cross-sectional observational study	20	USA/Chinese (55%) and Korean (45%)	PROMIS, PSQI	Self-reported	Insomnia
Hu et al. [64]	Case‒control study, Cross-sectional survey	293	China/Chinese	DSM-5 HAMD-17	Clinically diagnosed	Not specified
Huang et al. [65]	Cross-sectional study	61	China/Chinese	SAS-Chinese version 20-item	Self-reported	Not specified
Jiang et al. [66]	Case‒control, cross-sectional study	76	China/Chinese	DSM-IV, HAMA	Clinically diagnosed	Not specified
Jiang et al. [28]	Case‒control, cross-sectional study	76	China/Chinese	DSM-IV; HAMD; MADRS	Clinically diagnosed	Not specified
Kim et al. [67]	Case‒control, cross-sectional study	1238	South Korea	CES-D HC = CES-D < 16; MDD = 167 (13.5%) (CES-D ≥ 16)	Self-reported	Not specified
Kim et al. [68]	Case‒control, cross-sectional study	605	South Korea	BAI (≥8 indicated = clinical anxiety; 0 = HC)	Self-reported	Not specified
Knudsen et al. [69]	Case‒control, longitudinal study	59	Denmark/Danish	ICD-10	Clinically diagnosed	Not specified
Lai et al. [70]	Case‒control, cross-sectional study	55	China/Chinese	SCID-5-CV, HAMD scores greater than 17, HAMA, HCL-13	Clinically diagnosed	Anxiety symptom
Li et al. [71]	Case‒control, cross-sectional study	62	China/Chinese	ICD-10	Clinically diagnosed	Not specified
Ling et al. [72]	Case‒control, cross-sectional study	140	China/Chinese	HAMD, DSM-V, CCMD-3	Clinically diagnosed	Not specified
Liu et al. [29]	Cross-sectional observational study	100	China/Chinese	Rome III criteria, MINI, DSM-IV	Clinically diagnosed	Not specified
Liu et al. [73]	Case‒control, cross-sectional study	90	MDD: White = 80.1%, Hispanic = 6.7%; HC: White = 76.7%, Hispanic = 14.0%	PROMIS, SCID-5, C-SSRS, and SITBI	Self-reported	Not specified
Maes et al. [74]	Case‒control study, Cross-sectional survey	69	Thailand	DSM-5, MINI, HAMD, BDI	Clinically diagnosed	Not specified
Maes et al. [75]	Case‒control study, cross-sectional survey	69	Thailand	DSM-5, MINI, HAMD, BDI	Clinically diagnosed	Not specified
Malan-Muller et al. [76]	Case‒control study (PTSD and trauma-exposed controls), cross-sectional Survey	137	South Africa/mixed ancestry	DSM-5, CAPS-5, MINI, CTQ	Clinically diagnosed	Depression Anxiety disorders
Malan-Muller et al. [77]	Case‒control, cross-sectional, observational study	198	Spain/Spanish	Standardized self-report questionnaires validated for the Spanish population; CESD; STAI, PCL-5; CTQ	Self-reported	PTSD Depression Anxiety disorders
Mason et al. [78]	Case‒control, cross-sectional study	70	USA/Caucasian (75.7%) and Hispanic (34.3%)	SDS, GAD-7, QIDS-SR	Self-reported	Anxiety symptom
Naseribafrouei et al. [79]	Partially blinded observational study, case‒control	55	Norway	ICD-10, MADRS (score > 7)	Clinically diagnosed	Not specified
Ritchie et al. [80]	Clinical trial	117	Australia and New Zealand/Not specified	SCID-5-RV, BDI-II, GAD-7	Clinically diagnosed	Anxiety disorder
Rong et al. [40]	Case‒control, cross-sectional study	91	China/Chinese	DSM-5, HAMD-17 > 17	Clinically diagnosed	Not specified
Tsai et al. [81]	Case‒control, cross-sectional study	53	Taiwan/Not specified	DSM-IV-TR, MoCA, PSQI	Clinically diagnosed	Sleep disturbance, Cognitive impairment
Wang et al. [82]	Case-control, Longitudinal cohort study	50	China/Chinese	Chinese version of the STAI's, SDS	Self-reported	Depression
Yang et al. [83]	Case‒control, cross-sectional study	311	China/Chinese	DSM-IV, MINI	Clinically diagnosed	Not specified
Ye et al. [84]	Longitudinal observational study, Intervention cohort	54	China/Chinese	DSM-IV	Clinically diagnosed	Not specified
Yu et al. [85]	Case‒control, cross-sectional study	54	China/Chinese	DSM-IV	Not Specified	PCOS
Yirmiya et al. [86]	Case‒control, longitudinal study	232 mother-child dyads initially recruited	Israel	DSM-IV, DAWBA, CBCL	Clinically diagnosed	Not specified
Yuan et al. [87]	Case‒control, cross-sectional study	240	China/Chinese	UC = Mayo score > 2 and endoscopic sub-score > 0; Depression/Anxiety = PHQ-9 and GAD-7 scores > 4; HCs = PHQ-9 and GAD-7 scores < 5	Self-reported	Anxiety disorder
Zeamer et al. [88]	Longitudinal study	51	USA/White (61%), Black (25%), Hispanic (14%), Asian (2%) and Others (12%)	DSM-5, PCL-5, PROMIS, RPQ	Self-reported	Somatic symptom
Zhang et al. [89]	Two-arm parallel design, randomized, double-blind placebo-controlled trial	69	China/Chinese	DSM-5, HAMD-17; BDI	Clinically diagnosed	Not specified
Zhang et al. [90]	Case‒control, cross-sectional study	81	China/Chinese	ICD-10, HAMD, ISI, PSQ	Clinically diagnosed	Anxiety disorder; Fatigue; Daytime sleepiness
Zhang et al. [91]	Case‒control, cross-sectional study	81	China/Chinese	ICD-10, HAMD (>17), HAMA, FSS, ESS	Clinically diagnosed	Anxiety disorder; Fatigue; Daytime sleepiness
Zhang et al. [92]	Case‒control, cross-sectional study	39	China/Chinese	DSM-5	Clinically diagnosed	Anxiety disorder
Zhao et al. [93]	Case‒control, cross-sectional study	50	China/Han Chinese	DSM-5, HAMD-17, HAMA	Clinically diagnosed	Anxiety disorder
Zheng et al. [94]	Case‒control, cross-sectional study	60	China/Chinese	HAMA, HAMD	Self-reported	Anxiety symptom
Zhong et al. [95]	Case‒control, cross-sectional study	261	China/Chinese	DSM-IV, HDRS	Clinically diagnosed	Not specified
Zhou et al. [96]	Case‒control, cross-sectional study	57	China/Chinese	DSM-IV, HAMD-17, EPDS	Clinically diagnosed	Not specified
Zhou et al. [97]	Case‒control, cross-sectional study	171	China/Chinese	ICD-10, SDS, SAS,	Clinically diagnosed	Anxiety disorders
Zhou et al. [98]	Case‒control, longitudinal study	Baseline = 179; PPD = 88; Completed the 8-week PPD = 79	China/Chinese	DSM-5. SDS, SAS, HAMD-17 (scores between 7 and 24, Response rate based on score reduction ≥ 50% reduction or < 7 score)	Clinically diagnosed	Not specified
Zhu et al. [99]	Case‒control, cross-sectional study	69	China/Chinese	GAD-7, PHQ-9	Clinically diagnosed	Depression
Zhu et al. [100]	A randomized, double-blind, placebo-controlled design	90	China/Chinese	HAMA-14, AIS-8, HAMD-17	Clinically diagnosed	Anxiety symptom, depression, and insomnia
Zu et al. [101]	Case‒control, cross-sectional, observational study	114	China/Chinese	MINI, HAMD	Clinically diagnosed	Not specified

AIS-8 = Athens Insomnia Scale; BAI = Beck Anxiety inventory; BDI = Beck Depression Inventory; BP = Bipolar with Major Depressive Episode; CAPS-5 = Clinician-Administered PTSD Scale for DSM-5; CBCL = Child Behavior Checklist; CCMD-3 = Chinese Classification of Mental Disorders, Third Edition; CES-D = Center for Epidemiologic Studies Depression Scale; C-SSRS = Columbia-Suicide Severity Rating Scale; CTQ = Childhood Trauma Questionnaire; DAWBA = Development and Well-Being Assessment; DSM = Diagnostic and Statistical Manual of Mental Disorders; DSM-IV-TR = Diagnostic and Statistical Manual of Mental Disorders, Fourth Edition, Text Revision; EPDS = Edinburgh Postnatal Depression Scale; ESS = Epworth Sleepiness Scale; FFQ = Food Frequency Questionnaire; FSS = Fatigue Severity Scale; GAD = Generalized Anxiety Disorder; GAF = Global Assessment of Functioning; HAM-D/HDRS = Hamilton Depression Rating Scale; HAMA = Hamilton Anxiety Rating Scale; HCL-13 = Hypomania Checklist-13; IBS = Irritable Bowel Syndrome; ICD-10 = International Classification of Diseases, 10th Revision; IES-R = Impact of Event Scale—Revised; ISI = Insomnia Severity Index; LcS = *Lacticaseibacillus paracasei* strain Shirota; MADRS = Montgomery–Åsberg Depression Rating Scale; MINI = Mini International Neuropsychiatric Interview; MoCA = Montreal Cognitive Assessment; OHE = Overt Hepatic Encephalopathy; PHQ-9 = Patient Health Questionnaire-9; PHQ-15 = Patient Health Questionnaire-15; PNSS = Positive and Negative Syndrome Scale; PROMIS = Patient-Reported Outcomes Measurement Information System; PSS = Perceived Stress Scale; PSQI = Pittsburgh Sleep Quality Index; PSQ = Perceived Stress Questionnaire; QIDS-SR = Quick Inventory of Depressive Symptomatology—Self-Report; RPQ = Rivermead Post-Concussive Questionnaire; SAD-L = Seasonal Affective Disorder—Longitudinal Version; SAS = Self-Rating Anxiety Scale; SCID-5-CV = Structured Clinical Interview for DSM-5—Clinician Version; SITBI = Self-Injurious Thoughts and Behaviors Interview; SNRIs = Serotonin-Norepinephrine Reuptake Inhibitors; SSRIs = Selective Serotonin Reuptake Inhibitors; STAI = State-Trait Anxiety Inventory; UC = ulcerative colitis.

Thirty-eight (59.4%) studies were conducted with participants in China, and seven studies (11%) included participants from the United States. Three studies (4.69%) included participants from Taiwan, and two studies (3.13%) each included participants from South Africa, South Korea, Thailand, Spain, and Australia. One study each included participants from Japan, Germany, Norway, Denmark, and Israel. The HELIUS cohort included data from participants in the Netherlands, Turkey, Morocco, and Ghana (Table 1). European representation was limited, with only four studies (~6%) including participants from European countries. There is also a notable dearth of data from sub-Saharan Africa and low- and middle-income countries (LMICs).

Most studies (*n* = 51, ~80%) investigated depression, while seven studies (~11%) focused on PTSD, and six studies (~9%) focused on anxiety disorders. Participants were clinically diagnosed in 50 (~78%) studies (Table 1). This involved independent diagnosis by two psychiatrists or diagnosis based on the International Classification of Diseases, 10th edition (ICD-10),^102^ Diagnostic and Statistical Manual of Mental Disorders (DSM) fourth^103^^,^^104^ or fifth^103^ editions. In the remaining ~22% of studies, probable diagnoses were based on self-report measures or screening tools (Table 1). Twenty-five (~39.1%) cohorts exhibited comorbid conditions relevant to microbiome interpretation, including sleep disturbance and cognitive impairment in late-life depression,^81^ insomnia,^57^^,^^63^^,^^81^^,^^88^^,^^90^^,^^91^^,^^99^ and participants with co-occurring depression and anxiety^38^^,^^47^^,^^52-57^^,^^62^^,^^76-78^^,^^80^^,^^82^^,^^87-90^^,^^92^^,^^97^^,^^99^ (Table 1). However, only Malan-Müller et al.^77^ and Zeamer et al.^88^ accounted for comorbid depression, anxiety, and somatic symptoms in their analyses (Table 1).

### Methodological characteristics of articles reviewed

**Figure 2.**
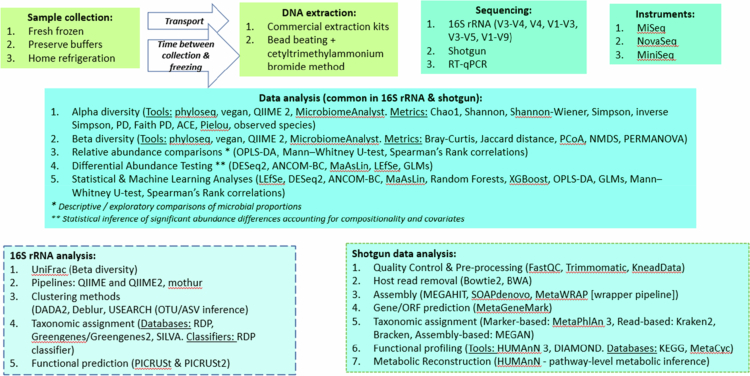
Microbiome analysis workflow in included studies. Summary of sample processing and analytical methods reported in the reviewed studies, covering collection, DNA extraction, sequencing (16S rRNA gene sequencing or whole-genome shotgun metagenomic sequencing), and downstream analyses.

The methodological characteristics, including sample collection, DNA extraction, amplicon selection and sequencing methods, and microbiome analysis methods are shown in Table 2(A‒D), Supplementary Table 2 and Figure 2. Stool samples were frozen after collection to preserve microbial integrity in 40 (~63%) studies. Time from collection to freezing varied, ranging from immediate freezing to within 15–30 min or 1 h after collection, to 6–72 h after collection, or even up to 1 week post-collection. Differences in handling procedures were noted, including the use of RNA stabilizing solution in collection tubes, freezing at home before laboratory delivery,^38^^,^^46^^,^^66^^,^^75^^,^^80^^,^^81^ and transportation in ice boxes. Most studies (*n* = 41, ~64%) stored samples at −80 °C for archival storage. Zeamer et al.^88^ implemented pre-storage treatment that involved heat inactivation at 65–70 °C to deactivate live bacteria.

**Table 2.** A–D: methodological characteristics of articles reviewed.

A. qPCR				
Study ID	Sample type	Sample collection	DNA extraction	Microbiome Analytical Approaches
Aizawa et al. [41]	Stool	Stool in RNAlater (2 mL); Ambion RNAlater (Thermo Fisher Scientific, Waltham, MA); Transport time and freezing temperature—NS	NS	Yakult Intestinal Flora-SCAN®

B. 16S rRNA gene amplicon sequencing												
Study ID	Sample type	Sample collection	DNA extraction	Region	Platform	Reads	Preprocessing tools/pipelines	Taxonomy	Database	Diversity	Abundance	Function
Bai et al. [42]	Stool	NS	E.Z.N.A.® soil DNA Kit (Omega Bio-tek, Norcross, GA, U.S.)	V3–V4	Illumina MiSeq	NS	Trimmomatic; FLASH; UCHIME; UPARSE	RDP Classifier	NS	NS	LEfSe; Random Forest	NT
Bajaj et al. [43]	Stool	NS	NS	NS	IonTorrent PGM	NA	NS	NS	NS	NS	LEfSe	PICRUSt
Bosch et al. [44]	Stool	Fresh/frozen; home freeze if >6 h delay; −20 °C storage ≤ 4 weeks; then −80 °C; local −20 °C storage	NS	V4	Illumina MiSeq V2	Paired-end 2× 250 bp	FastQC; USEARCH; UNOISE3	SINTAX	GreenGenes v13.5; SILVA 132	IQ-TREE; R- packages	NS	NT
Busch et al. [45]	Stool	Fresh; DNA/RNA Shield (Zymo); frozen within 15–30 min; stored at −80 °C	Genomic DNA Mini Kit (Invitrogen, Thermo Fisher Scientific, Langenselbold, Germany)	V3–V4	Illumina iSeq V2	Paired-end 2× 150 bp	minimap2; LotuS v2.24; VSEARCH UCHIME; CD-HIT	RDP	RDP	MAFFT; fasttree2; R-phyloseq	QIIME2	NT
Caso et al. [46]	Stool	Fresh; icebox transport ≤1 h; stored at −80 °C	QIAamp DNA Stool Mini Kit (QIAGEN, Hilden, Germany); glass-bead beating	V3–V4	Illumina MiSeq V3	Paired-end 2× 300 bp	prinseq-lite; fastq-join in ea-tools suite	RDP	RDP	R-packages; QIIME	NS	NT
			steps on a Mini-beadbeater (FastPrep; Thermo Electron Corp.)									
Chen et al. [48]	Stool	Stored at −80 °C; no further details provided	PowerSoil® DNA Isolation Kit (MO BIO Laboratories, Carlsbad, CA, USA)	V3–V5	454	NS	Mothur	RDP	RDP	NS	Random forest; LEfSe	NT
Chen et al. [49]	Stool	Fresh sterile cup; frozen at −80 °C within 15 min	QIAamp DNA Stool Mini Kit (Qiagen, Valencia, CA, USA)	V3–V4	Illumina MiSeq	Paired-end 2× 250 bp	USEARCH; UCHIME; UPARSE	RDP	SILVA vSSU132	QIIME	QIIME; LEfSe	NT
Chen et al. [50]	Stool	NS	PowerSoil® DNA Isolation Kit (MO BIO Laboratories, Carlsbad, CA, USA)	V3–V5	Roche 454	NS	Mothur	RDP	RDP		LEfSe	NT
										NS		
Chen et al. [51]	Stool	Fresh sterile cup; frozen at −80 °C within 30 min	Qiagen QIAamp DNA Stool Mini Kit (Qiagen)	V3–V4	Illumina MiSeq	NS	USEARCH; UPARSE	RDP	NS	Mothur v.1.39.5; QIIME	Random Forest	KEGG
Chen et al. [52]	Stool	Refrigerated at home; delivered at 4 °C next day; stored at −80 °C	QIAamp DNA Stool Mini Kit (QIAGEN Inc., USA) and custom protocol for phenol–chloroform extraction method	V3–V4	Illumina MiSeq	Paired-end 2× 250 bp; paired-end 2× 300 bp	FASTX	QIIME	Greengenes v13.5	NS	NS	KEGG
Fatt et al. [53]	Stool	Chilled/frozen at home; returned within 48 h; frozen at −80 °C; collected within 1 week of clinical assessments	Mechanical and enzymatic lysis; followed by phenol:chloroform extraction and a clean-up step	V3–V4	NS	NS	DADA2	NS	SILVA v128	NS	Random Forest; WGCNA	ALDEx2; WGCNA
Chung et al. [54]	Stool	Collected within 2 weeks of interview and assessments; delivered at 4 °C; stored at −80 °C; transport time not specified	QIAamp DNA Stool Mini Kit (QIAGEN Inc., USA) and custom protocol for phenol–chloroform extraction method	V3–V4	Illumina MiSeq; MiniSeq	NS	PEAR v0.9.8; DADA2	QIIME	Greengenes	QIIME	ANCOM	NT
				V4								

Dong et al. [55]	Stool	Fresh; frozen immediately in sterile cups at collection; stored at −80 °C before analysis	QIAamp DNA Stool Mini Kit (QIAGEN, Hilden, Germany); glass-bead beating steps on a Mini-beadbeater (FastPrep; Thermo Electron Corp.)	V3–V4	Illumina MiSeq	NS	UPARSE; UCHIME	RDP	SILVA vSSU138	QIIME2	QIIME2	PiCRUST
Dong et al. [56]	Stool	Fresh; frozen immediately in sterile cups at collection; stored at −80 °C before analysis	QIAamp DNA Stool Mini Kit (Qiagen, Hilden, Germany)	V3–V4	Illumina MiSeq	NS	UPARSE; UCHIME v4.1; UNOISE3	RDP	SILVA vSSU138	Vegan, R package	STAMP; Welch's *t*-test	NT
Gao et al. [58]	Stool	Fresh fecal samples collected in MGIEasy Stool DNA preservation tubes; stored at −80 °C	E.Z.N.A® DNA Kit (Omega Biotek, Norcross, GA, U.S.)	V1–V9	PacBio Sequel	NS	Deblur; SMRT Link v9.0; SMRT Portal	USEARCH v11	SILVA database vSSU132	Vegan, R package	Random Forest	NT
Gao et al. [59]	Stool	Fresh, mid-stool; clean toilet; sealed, labeled, and stored immediately at −80 °C; freeze–thaw cycles avoided	QIAamp DNA Stool Mini Kit (Qiagen, Hilden, Germany)	V3–V4	Illumina MiSeq	Paired-end 2× 300 bp	QIIME	NS	Greengenes	NS	LEfSe; Metastats	NT
Gonzalez-Mercado et al. [60]	Stool	NS	PowerSoil® DNA Isolation Kit (MO BIO Laboratories, Carlsbad, CA, USA)	V3–V4	NS	NS	Trim Galore! v0.44; DADA2; QIIME2	QIIME2	SILVA v132	QIIME2	QIIME2	NT
Guo et al. [61]	Stool	Fresh; immediately frozen at −80 °C	QIAamp DNA Stool Mini Kit (QIAGEN, Hilden, Germany)	V3–V4	Illumina NovaSeq PE250	Paired-end 2× 250 bp	QIIME2	NS	NS	QIIME2	LEfSe	NT
Hemmings et al. [62]	Stool	NS	PSP® Spin Stool DNA Plus Kit (STRATEC Molecular, Birkenfeld, Germany)	V3–V4	Illumina HiSeq	Paired-end 2× 100 bp	Illumina Casava Pipeline v1.8.2; Illumina Chastity filter; FASTQC v0.10.0	QIIME	Greengenes	QIIME	Random Forest	NT
Hope et al. [63]	Stool	Collected at home using a kit with three tubes (Fisher Scientific), placed in biohazard and padded freezer bags with ice packs, frozen immediately, and delivered or shipped within 24 h; stored at −80 °C until DNA extraction	PowerSoil isolation kit (Mo Bio Laboratories, Carlsbad, CA, U.S.)	V3–V4	Illumina MiSeq V3	Paired-end 2× 100 bp	DADA2; QIIME2	QIIME2	SILVA	NS	LEfSe	NT
Huang et al. [65]	Stool	Fresh stool in 5 mL sterile tubes (screwcap with spoon), collected ±1 week of MRI, stored at −80 °C immediately	QIAamp DNA Stool Mini Kit (QIAGEN, Hilden, Germany)	V3–V4	Illumina MiSeq V3	Paired-end 2× 300 bp	Illumina Standard Analysis Pipeline	RDP	Greengenes	QIIME	QIIME	NT
Jiang et al. [66]	Stool	Fresh; self-collected in sterile plastic cups; delivered within 30 min; stored at −80 °C	QIAamp DNA Stool Mini Kit (Qiagen, Hilden, Germany); glass-bead beating step on a mini bead beater (FastPrep Thermo Electron Corp.)	V3–V4	Illumina MiSeq V3	Paired-end 2× 300 bp	Trimmomatic; FLASH; UCHIME	QIIME	NS	NS	LEfSe	NT
Jiang et al. [28]	Stool	Fresh; collected in sterile plastic cup post-assessment; kept in icebox; delivered within 15 min; stored at −80 °C	QIAamp DNA Stool Mini Kit (Qiagen, Hilden, Germany); glass-bead beating step on a mini bead beater (FastPrep; Thermo Electron Corp.)	V1–V3	Roche 454 FLX system		Mothur v1.25.0; Titanium PyroNoise software	RDP; QIIME2	NS	Mothur	Metastats; LEfSe	NT
Kim et al. [67]	Stool	Fresh; collected at home in sterile screw-cap containers (no preservative), frozen at −20 °C, delivered within 24 h, stored at −70 °C	PowerSoil® DNA Isolation Kit (MO BIO Laboratories, Carlsbad, CA, USA)	V3–V4	Illumina MiSeq	Paired-end 2× 300 bp	DADA2	QIIME2	SILVA database v132 V4	R packages	MaAsLin	PICRUSt2
Kim et al. [68]	Stool	Self-collected fecal swabs, frozen at −20 °C, stored at −70 °C for 24 h	PowerSoil® DNA Isolation Kit (MO BIO Laboratories, Carlsbad, CA, USA)	V3–V4	Illumina MiSeq	NS	DADA2	QIIME2	SILVA v r138.1	NS	MaAsLin; LEfSe	NT
Knudsen et al. [69]	Stool	Fresh; home-collected, stored at −20 °C (max 72 h), delivered in cooling bag, stored at −80 °C	QIAamp Powerfecal DNA kit (QIAGEN)	V4	Illumina MiSeq V3	Paired-end 2× 300 bp	USEARCH v11	QIIME2	SILVA v138 SSU	MAFFT and fasttree2	ANCOM-BC.	NT
Li et al. [71]	Stool	Fresh; collected within 24 h of admission in a sterile plastic box; stored at −80 °C	StoolGen DNA kit (Beijing Youji Technology Co., China).	V4	Illumina MiSeq V3	Paired-end 2× 250 bp	FLASH v1.2.11	RDP v2.2	Greengenes v2013_5_99	Mothur	NS	NT
Ling et al. [72]	Stool	Fresh; 2 g of stool; sterile plastic cup; stored at −80 °C within 15 min until use	QIAamp DNA Stool Mini Kit (Qiagen, Hilden, Germany)	V3–V4	Illumina MiSeq V3	Paired-end 2× 300 bp	QIIME2 v2020.11	RDP; UCLUST v1.2.22	Greengenes v13.8.	QIIME2	LEfSe	PiCRUSt
Liu et al. [29]	Stool	Fresh; stored at −80 °C immediately after collection	PowerSoil® DNA Isolation Kit (MO BIO Laboratories, Carlsbad, CA, USA)	V1–V3	Roche 454 GS FLX + Titanium platform		MID tags; Mothur	BLAST	RDP; BLAST	NS	NS	NT
Liu et al. [73]	Stool	OMNIgene•GUT kit (DNA Genotek), stored at −80 °C upon receipt	ZymoBIOMICS™ DNA Kit (Zymo Research)	V4	Illumina MiSeq	Paired-end 2× 250 bp	DADA2; QIIME2	QIIME2	Silva	QIIME2; R packages	LEfSe	NT
Maes et al. [74]	Stool	Fresh; sterile tubes with 2 mL DNA/RNA Shield™ (Zymo Research); stored at −20 °C until use	ZymoBIOMICS™ DNA Miniprep Kit (ZYMO Research, USA)	V1–V9	ONT MinION Mk1C		Guppy basecaller v6.0.7, MinIONQC, NanoCLUST, and Porechop v0.2.4	NanoCLUST	RDP	QIIME2	LEfSe;	NT
Maes et al. [75]	Stool	Fresh; sterile tubes with 2 mL DNA/RNA Shield™ (Zymo Research); stored at −20 °C until use	ZymoBIOMICS™ DNA Miniprep Kit (ZYMO Research, USA)	V1–V9	ONT MinION Mk1C		Guppy basecaller v6.0.7, MinIONQC, NanoCLUST, and Porechop v0.2.4	NanoCLUST	RDP	QIIME2	LEfSe;	NT
Malan-Muller et al. [76]	Stool	PSP Spin Stool DNA Plus Kit (STRATEC); transport time and storage temperature not specified	PSP Spin Stool DNA Plus Kit (STRATEC Molecular, Birkenfeld, Germany)	V3–V4	Illumina MiSeq V2	Paired-end 2× 300 bp	FastQC; MultiQC; DADA2	RDP	RDP	R-packages	RandomForests	NT
Malan-Muller et al. [77]	Stool	PSP Spin Stool DNA Plus Kit (STRATEC); transport time and storage temperature not specified	PSP Spin Stool DNA Plus Kit (STRATEC Molecular, Birkenfeld, Germany)	V3–V4	Illumina MiSeq	Paired-end 2× 300 bp	FastQC; MultiQC; DADA2;	RDP	RDP Train Set 18 (v11.5)	R-packages	*CoDaSeq* package	NT
Mason et al. [78]	Stool	Fresh; stored immediately in home freezers; transferred to the lab; stored at −80 °C until extraction; transport time not specified	Custom protocol based on Goodman et al., 2011	V4	Roche 454 Titanium		UCLUST	NS	SILVA	NS	NS	NT
Naseribafrouei et al. [79]	Stool	Fresh; frozen at −20 °C at home; transported to centralized −70 °C storage; hospitalized patients' samples were taken directly to the centralized freezer	Mag™ Mini Kit (LGC, Middlesex, UK)	NS	Illumina MiSeq	Paired-end 2× 250 bp	QIIME; UCLUST	RDP	RDP	NS	NS	PLS-DA
Ritchie et al. [80]	Stool	Fresh; Sarstedt containers with 5 mL RNAlater®, immediately frozen; returned on ice; stored at −80 °C	NS	V3–V4	Illumina MiSeq V3	Paired-end 2× 300 bp	Metagenomics Workflow; MiSeq Pipeline	Illumina-proprietary classification algorithm	Greengenes taxonomy 13.5	R packages	DESeq2 R package	NT
Tsai et al. [81]	Stool	Fresh; RNA stabilizing reagent (RNALater); stored at −80 °C	Bead-beating method	V3–V4	Illumina MiSeq	NS	QIIME2	QIIME2	MetaSquare; SILVA; Greengenes; RDP; HOMD; Ezbiocloud	MAFFT and fasttree2	LEfSe	NT
Wang et al. [82]	Stool	Fresh; collected at baseline, week 8, and week 12; sent to the research assistant the same day, and immediately frozen at −80 °C until analysis	TIANamp Stool DNA Kit (TIANGEN Biotech Co. Ltd., Beijing, China)	V3–V4	Illumina MiSeq	Paired-end 2× 300 bp	QIIME2	QIIME2	MetaSquare; SILVA; Greengenes; RDP; HOMD; Ezbiocloud	NS	LEfSe	KEGG
Ye et al. [84]	Stool	Fresh; collected using disposable feces collectors; frozen at −80 °C	PowerSoil® DNA Isolation Kit (MO BIO Laboratories, Carlsbad, CA, USA)	V3–V4	Illumina HiSeq	Paired-end 2× 250 bp.	Trimmomatic; FLASH; UCHIME; UPARSE	RDP	NS	NS	LEfSe	NT
Yirmiya et al. [86]	Stool	Fresh; collected during home visits; kit, transport time, and freezing temperature not specified	NS	V4	Illumina MiSeq	NS	QIIME2	QIIME2	NS	NS	NS	NT
Yuan et al. [87]	Stool	Fecal biopsy from large intestine; collection and storage details not specified	AllPrep DNA/RNA Mini Kit (Qiagen, Hilden, Germany)	V3–V4	Illumina MiSeq	NS	Illumina Analysis Pipeline	RDP	SILVA v128	R-packages	NS	PICRUSt
Zhang et al. [89]	Stool	NS	E.Z.N.A. Soil DNA kit (Omega Bio-Tek, Norcross, GA, USA)	V3–V4	Illumina MiSeq	Paired-end 2× 250 bp	QIIME; UCHIME	UPARSE; RDP	SILVA v132	NS	LEfSe	NT
Zhang et al. [90]	Stool	Fresh; RNA stabilization solution (Tiny-Gen, China); ≥1 g within 2 d post-admission; stored at −80 °C	NS	V4–V5	Illumina NovaSeq6000 SP 500	Paired-end 2× 250 bp	Trimmomatic; FLASH; UPARSE; Mothur	UPARSE	NS	Mothur	ANOSIM; LEfSe	NT
Zhang et al. [91]	Stool	Fresh; RNA stabilization solution (Tiny-Gen, China); ≥ 1 g within 2 d post-admission; stored at −80 °C	NS	V4–V5	Illumina NovaSeq6000 SP 500	Paired-end 2× 250 bp	UPARSE; Trimmomatic; mothur; FLASH	UPARSE	NS	Mothur; Metastats	Metastats	NT
Zhang et al. [92]	Stool	Fresh; collected on behavioral assessment day; frozen immediately; stored at −80 °C	PowerSoil DNA Isolation Kit (Qiagen, Germany)	V4	Illumina MiSeq	Paired-end 2 250 bp	UPARSE; UCHIME	RDP; QIIME	Greengenes v201305	NS	Random Forest Analysis	NT
Zheng et al. [94]	Stool	Fresh; tube with 2 mL preservative; frozen at −80 °C	QIAamp DNA Stool Mini Kit (QIAGEN)	V3–V4	Illumina MiSeq	Paired-end 2× 300 bp	Trimmomatic; FLASH; UPARSE; Mothur	UPARSE	NS	NS	NS	NT
Zhong et al. [95]	Stool	NS	PowerSoil® DNA Isolation Kit (MO BIO Laboratories, Carlsbad, CA, USA)	V4–V5	Illumina MiSeq and NovaSeq	MiSeq: paired-end 2× 300 bp; NovaSeq: paired-end 2× 250 bp	FASTP; FLASH; UPARSE	RDP	NS	NS	NS	NT
Zhou et al. [96]	Stool	Fresh; sterile plastic cup; immediately stored at −20 °C; transported and stored at −80 °C storage	PowerSoil® DNA Isolation Kit (MO BIO Laboratories, Carlsbad, CA, USA)	V4	Illumina MiSeq V3	Paired-end 2× 150 bp	QIIME2	QIIME2	SILVA	QIIME2	LEfSe	NT
Zhou et al. [97]	Stool	Fresh; immediately transferred to the laboratory; stored at −80 °C	NS	V3–V4	Illumina MiSeq	Paired-end 2× 300 bp	ChimeraSlayer	NS	NS	QIIME	LEfSe	NT
Zhou et al. [98]	Stool	Fresh; at baseline in sterile cups, frozen at −80 °C immediately after defecation	PowerSoil® DNA Isolation Kit (MO BIO Laboratories, Carlsbad, CA, USA)	V4	Illumina MiSeq V3	Paired-end 2× 150 bp	QIIME2.0	QIIME2	SILVA	QIIME2	LEfSe	NT
Zhu et al. [99]	Stool	Fresh; smeared on FOBT cards before endoscopy; sealed, stored at −80 °C, and shipped on dry ice	PowerSoil® DNA Isolation Kit (MO BIO Laboratories, Carlsbad, CA, USA)	V4	Illumina Miniseq	Paired-end 2× 300 bp	Illumina MiniSeq Reporter	QIIME	Greengenes v13_8	QIIME	LEfSe	NT
Zhu et al. [100]	Stool	NS	TIANamp DNA Kit (QIAGEN)	V4	Illumina HiSeq2000	Paired (length not specified)	FLASH; UPARSE	NS	NS	QIIME2	NS	NT
Zu et al. [101]	Stool	Fresh; middle portion; ice box; transferred to lab; packed in cryotubes, stored at −80 °C	E.Z.N.A.® soil DNA Kit (Omega Bio-tek, Norcross, GA, US)	V4	Illumina MiSeq PE300 platform; UPARSE; Fastp v0.19.6	Paired (length not specified)	UPARSE; Fastp	RDP v2.11	SILVA	Mothur v1.30.1	NS	NT

C. Metaproteomics												
Study ID	Sample type	Sample collection	Protein extraction	Targets	Relative abundance	Differential abundance	Biomarkers					
Chen et al. [47]	Stool	Fresh, delivered within 15 min; stored at −80 °C	Proteins were extracted using SDS buffer, followed by boiling, sonication, trypsin digestion, and peptide quantification for LC‒MS/MS analysis	Peptide and protein identification	Normalized iTRAQ-labeled bacterial peptide intensities summed by COG categories	Identified using ≥1.5-fold change, permutation tests, and Mann‒Whitney tests (*p* < 0.05)	The study identified differentially expressed proteins. A total of 279 significantly differentially expressed bacterial proteins were detected, with statistical significance set at *p* < 0.05					

D. Metagenomics													
Study ID	Sample type	Sample collection	DNA extraction	Platform	Reads	Preprocessing	Assembly	ORF prediction	Clustering	Taxonomy	Function	Diversity	Abundance
Chen et al. [51]	Stool	Fresh sterile cup; frozen at −80 °C within 30 min	Qiagen QIAamp DNA Stool Mini Kit (Qiagen)	Illumina NovaSeq 6000	Paired-end 2× 150 bp reads	Sequence filtering: remove bases with mass value less than 20 and sequences less than 30bp	Megahit	MetaGeneMark	CD-HIT; Bowtie2; eXpress	MetaPhlAn v2.0	DIAMOND with KEGG; Bowtie1; eXpress	R vegan package v2.5-2	eXpress v1.5.1 MetaPhlAn2
Dong et al. [56]	Stool	Fresh; frozen immediately in sterile cups at collection; stored at −80 °C before analysis	QIAamp DNA Stool Mini Kit (Qiagen, Hilden, Germany)	Illumina NovaSeq	NS	MetaWRAP	MetaWRAP	Prokka	MetaPhlAn3; MetaWRAP	MetaPhlAn3	KEGG	R vegan package v2.5-7	MetaPhlAn3
Hu et al. [64]	Stool	Fresh stool in sterile tubes (7–10 am), stored at 4 °C, transferred to −80 °C within 6 h	E.Z.N.A. Soil DNA Kit (Omega Bio-Tek, Norcross, GA, USA)	Illumina NovaSeq	NS	Sickle	MEGAHIT	Metagen	CD-HIT	Metaphlan2 v2.0	Diamond v0.8.35; KEGG db	Past 4.0 PERMANOVA	LEfSe; Random Forest; ROC curve
Lai et al. [70]	Stool	Fresh; collected the completion of questionnaire assessments; immediately frozen at −80 °C	StoolGen DNA kit (CWBiotech Co., China)	Illumina HiSeq2500	Paired-end 2× 150 bp reads	NS	NS	NS	NS	MEGAN5	KEGG	NS	LEfSe; Random Forest
Rong et al. [40]	Stool	Fresh; stored at −80 °C; Transport time not specified	StoolGen DNA kit (CWBiotech Co., Beijing, China)	Illumina HiSeq2500	Paired-end 2× 150 bp	NS	NS	NS	NS	MEGAN5	KEGG	NS	NS
Yang et al. [83]	Stool	NS	E.Z.N.A. Soil DNA Kit (Omega Bio-tek, Norcross, GA, USA)	Illumina NovaSeq		MetaPhlAn3	MEGAHIT		CD-HIT	MetaPhlAn3	DIAMOND; KEGG	PERMANOVA	LEfSe; KEGG
Yu et al. [85]	Stool	Fresh; collected at the second visit using home kits in the nonmenstrual morning and stored immediately at −80 °C	Cetyltrimethylammonium bromide method	Illumina HiSeq 2500	Paired-end 2× 150 bp Shotgun	Trimmomatic; Bowtie2; FASTQC v0.11.9	NS	NS	NS	KraKen2 v2.0.7; Bracken V2.0	Human3; GO; KEGG; MetaCyc	NS	LEfSe
Zeamer et al. [88]	Stool	OMNIgene•GUT kits (OMR-200); heat-inactivated at 65–70 °C for 1 h; stored at −80 °C; transport time not specified	QIAGEN DNeasy PowerSoil Pro Kits (QIAGEN, catalog no. 47016)	Illumina NextSeq 500	Paired-end 2× 150 bp	KneadData	NS	MetaPhlAn3	NS	Metaphlan3	HUMAn3	NS	MetaPhlAn3; HUMAn3
Zhao et al. [93]	Stool	Fresh; collected same day as symptom assessment; frozen immediately; stored at −80 °C	NS	Illumina NovaSeq 6000	Paired-end 2× 150 bp	Readfq	SOAPdenovo v2.04	MetaGeneMark v2.10	CD-HIT v.4.5.8	DIAMOND	DIAMOND; KEGG	R ade4 v2.15.3	Metastats; LEfSe; Random Forest

Microbial DNA extraction methods varied across studies, with most (*n* = 42, ~66%) utilizing commercially available extraction kits (Table 2(A‒D), Supplementary Table 2 and Figure 2). Among the extraction kits, the QIAamp DNA Stool Mini Kit (QIAGEN) was the most frequently used, appearing in 13 studies (~20%). Other commonly used kits included the DNA Isolation Kit (MoBio, Carlsbad, CA, USA) (*n* = 8; ~13%), PowerSoil Isolation Kit (QIAGEN) (*n* = 5; ~8%), and the E.Z.N.A.® Soil DNA Kit (Omega Bio-tek) (*n* = 5; ~8%). Less frequently used kits included the ZymoBIOMICS™ DNA Miniprep Kit (ZYMO Research) (*n* = 3; ~5%), PSP Spin Stool DNA Plus Kit (STRATEC Molecular) (*n* = 3, ~5%), and FastDNA SPIN Kit for Feces (MP Biomedicals) (*n* = 2; ~3%) (Table 2(A‒D), Supplementary Table 2 and Figure 2).

Across the 64 included studies, the majority employed 16S rRNA gene amplicon sequencing, with the V3–V4 region most commonly targeted (Table 2(A‒D), Supplementary Table 2 and Figure 2). Only a minority of studies have used shotgun metagenomics, reflecting the limited adoption of whole-metagenomic approaches in psychiatric cohorts (Table 2(A‒D)). Specifically, 54 studies (~84%) targeted the 16S rRNA gene, with the V3–V4 region being the most analyzed (*n* = 30, ~55.56%), followed by the V4 region (*n* = 13, ~24.1%). Ten studies (~18.52%) focused on the V1‒V3 (*n* = 2, ~3.7%), V3‒V5 (*n* = 2, ~3.7%), V4‒V5 (*n* = 3, ~5.56%), and full-length (V1-V9) (*n* = 3, ~5.56%) regions. Chung et al.^54^ targeted both the V3–V4 and V4 regions. Two studies^43^^,^^79^ did not specify the regions targeted (Table 2(A‒D), Supplementary Table 2 and Figure 2). Aizawa et al.^41^ utilized RT‒qPCR (Yakult Intestinal Flora-SCAN®) to quantify gut taxonomic groups. Shotgun metagenomic sequencing was employed in nine studies (~14%) (Table 2(A‒D), Supplementary Table 2 and Figure 2). The nine studies utilized Illumina sequencing platforms (MiniSeq, HiSeq2500, HiSeq4000, HiSeq X Ten, NovaSeq 6000, and NextSeq 500). Two studies^51^^,^^56^ utilized both 16S rRNA gene amplicon sequencing and shotgun metagenomic sequencing.

Bioinformatic preprocessing and quality control steps varied across studies (Table 2(A‒D), Supplementary Table 2 and Figure 2). Common steps included adapter trimming and read merging using tools such as FLASH,^105^ fastq-join,^106^ Trimmomatic,^107^ and FASTP.^108^ Chimera detection was performed using UCHIME,^109^ and operational taxonomic unit (OTU) clustering at 97% similarity was standard using UPARSE,^110^ CD-HIT,^111^ or Mothur.^112^ However, a number of studies employed amplicon sequence variant (ASV)-based approaches using Divisive Amplicon Denoising Algorithm 2 (DADA2)^113^ within the Quantitative Insights Into Microbial Ecology 2 (QIIME2)^114^ framework (Table 2(A‒D), Supplementary Table 2 and Figure 2). Quality filtering thresholds (e.g. Q < 20 over 50 bp, no ambiguous bases) and the reporting of these metrics varied. Taxonomic classification methods also varied considerably across studies. Among the 16S rRNA gene amplicon sequencing studies, taxonomy assignment was most commonly performed using the Ribosomal Database Project (RDP) Classifier^115^ (*n* = 13) trained on databases such as SILVA^114^^,^^115^ (*n* = 4), Greengenes^115^ (*n* = 2), and RDP (*n* = 1). Studies using QIIME or QIIME2 (*n* = 17, 26.6%) frequently employed Naïve Bayes classifiers^116^ trained on SILVA (*n* = 10) or Greengenes (*n* = 6). Others include SINTAX with Greengenes and SILVA, UCLUST,^110^ BLAST,^110^ and NanoCLUST.^117^ The SILVA database^118^ (versions v132 and vSSU138) was the most frequently used reference for taxonomy assignment (*n* = 16). However, nine studies did not specify the taxonomy assignment tool or reference database used (Table 2(A‒D), Supplementary Table 2 and Figure 2).

More complex pipelines for taxonomic profiling and functional prediction were used in the studies that employed shotgun metagenomics (Table 2(A‒D), Supplementary Table 2 and Figure 2). Metagenomic phylogenetic analysis^119^ version 2 (MetaPhlAn2) was used in three studies, while MetaPhlAn3 featured in two studies. Double index alignment of next-generation sequencing data^120^ (DIAMOND) was used for taxonomic profiling in two studies, with Zhao et al.^93^ applying the lowest common ancestor (LCA)^121^ algorithm referencing the National Center for Biotechnology Information nonredundant protein sequence^122^ (NCBI NR) database. Yu et al.^85^ employed Kraken2 + Bracken trained on a local NCBI nucleotide and RefSeq database. One study^40^ did not report the taxonomic assignment method, limiting reproducibility. Lai et al.^87^ used the MEtaGenome ANalyzer^121^ version 5 (MEGAN5) for both sequence analysis and taxonomic profiling, with filtering based on a prevalence threshold of <80% across all samples. Functional prediction and annotation of genes found in microbial communities was commonly performed using Kyoto Encyclopedia of Genes and Genomes (KEGG).^123^

### Alpha and beta diversity

Alpha diversity is a measure of microbial richness and evenness within individual samples.^124^ In this review, 61 (~95.3%) studies employed sequencing-based approaches (Table 2(A‒D), Supplementary Table 2 and Figure 2). Among these, two (~3.28%) studies^60^^,^^93^ did not process or specify alpha diversity metrics. Beta diversity was either not processed or not specified in three (~4.92) studies.^65^^,^^88^^,^^93^ Beta diversity indices were not specific to five (~8.2)^42^^,^^48^^,^^50^^,^^51^^,^^85^ studies. One study^53^ performed alpha diversity analysis but did not report the results.

In the included studies, Shannon's diversity index (*n* = 51, ~80%) was the most commonly reported alpha diversity measure, followed by Chao1 (*n* = 30, ~47%), and then Simpson (*n* = 28, ~44%), ACE (*n* = 18, ~28%), observed OTUs/ASVs (*n* = 13, ~20%), and Faith's phylogenetic diversity (*n* = 10, ~16%) (Tables 3–5). The findings on alpha diversity were inconsistent across all the disorders included (Tables 3–5). Of the seven studies reporting PTSD, four studies ^43^^,^^58^^,^^77^^,^^86^ reported a significant reduction in alpha diversity among individuals with PTSD (Table 3). However, two studies^62^^,^^76^ reported no significant difference in alpha diversity between cases and controls.

**Table 3.** Alpha and beta diversity outcomes in individuals with PTSD compared to controls.

Study ID	Alpha diversity						Beta diversity						
	Shannon	Chao1	Simpson	ACE	Observed OTU/ASV	Faiths PD	BC	WU	UU	AD	JD	Analysis	Finding
Bajaj et al. [43]	↓												
Gao et al. [58]	↓	↓	↓	↓	↓		*					PCoA	*
Hemmings et al. [62]	NS						NS	NS	NS			PCoA	NS
Malan-Muller et al. [76]			NS							NS		MDS	NS
Malan-Muller et al. [77]			↓							NS		MDS	NS
Yirmiya et al. [86]	↓									*			
↓/*	**3**	**1**	**2**	**1**	**1**		**1**			**1**			**1**
NS	**1**		**1**				**1**	**1**	**1**	**2**			**2**

↑ = increase; ↓ = decrease; NS = not significant; * = significant; ACE = abundance-based coverage estimator; AD = Aitchison distance; ASV = amplicon sequence variant; BC = Bray–Curtis; JD = Jaccard dissimilarity; MDS = multidimensional scaling; OTU = operational taxonomic unit; PCoA = principal coordinates analysis; UU = unweighted UniFrac; WU = weighted UniFrac.

**Table 4.** Alpha and beta diversity outcomes in individuals with depression compared to controls.

Study ID	Alpha diversity						Beta diversity						
	Shannon	Chao 1	Simpson	ACE	Observed OTU/ASV	PD	BC	WU	UU	AD	JD	Analysis	Finding
Bai et al. [42]	NS	NS					NS					OPLS-DA	*
Bosch et al. [44]	↓						*	*				PCoA	*
Busch et al. [45]	↓		↓				NS					NMDS	NS
Caso et al. [46]	NS						NS				NS	PCoA PERMANOVA	NS
Chen et al. [47]													
Chen et al. [48]	NS		NS		NS	NS	NS					PCoA PLS-DA	*
Chen et al. [50]		NS		NS			NS					OPLS-DA	*
Chen et al. [51]	NS		NS	NS				*	*		*	PCoA	*
Chen et al. [52]	NS			NS			NS					PCoA	NS
Fatt et al. [53]							NS					WCNA	*
Chung et al. [54]	NS			NS	NS	NS		*	*			PERMANOVA	*
Dong et al. [55]	NS	NS	NS	NS			NS					PCA	NS
Dong et al. [56]	NS	NS					NS					NMDS	NS
Gao et al. [59]	NS	↓	↓		NS	NS					* (R) NS (TR)	PCoA	* (R) NS (TR)
Guo et al. [61]			↓					*				PCoA	*
Hope et al. [63]	↓	↓							*			PCoA PERMANOVA	*
Hu et al. [64]	↓		↓ (Moderate & severe MDD) NS (mild MDD)					*				PCoA	*
Jiang et al. [28]	↓	NS		NS						NS		PCoA	NS
Kim et al. [67]	↓					NS	*	NS	NS		NS	PERMANOVA	* NS
Knudsen et al. [69]	NS				NS	NS	NS	NS	NS			PERMANOVA	NS
Lai et al. [70]	NS						*					PCoA	*
Li et al. [71]	↓	↓	↑	↓									
Ling et al. [72]	NS	↑	NS	↑	↑		*	*	*		*	PCoA	*
Liu et al. [29]	↓											PCA	*
Liu et al. [73]	NS		NS		NS	↓	*	*	*			PCA	NS
Maes et al. [74]	NS	NS					NS						
Maes et al. [75]	NS	NS					NS						
Mason et al. [78]	NS							NS				PERMANOVA	NS
Naseribafrouei et al. [79]			NS				NS					PCA PLS-DA	NS
Ritchie et al. [80]	NS	NS	NS	NS			NS	NS				PCoA PERMANOVA	NS
Rong et al. [40]	NS	↓	NS				*						*
Tsai et al. [81]	NS					↓			*			PCoA	*
Yang et al. [83]	NS	NS	NS				NS					PCoA PERMANOVA	*
Ye et al. [84]	↓	↓							*			PCoA	*
Yu et al. [85]		↑					NS					PCoA	*
Yuan et al. [87]	↓					↓			*			PCoA	*
Zhang et al. [89]	NS	NS					*					PCoA ANOSIM	*
Zhang et al. [90]	NS	NS	NS	NS			*	*	*		*	PCoA	*
Zhang et al. [91]	NS	NS	NS	NS			NR						
Zhang et al. [92]	NS	NS	NS							NS		PCA	NS
Zhao et al. [93]												PCA	*
Zheng et al. [94]	NS	NS	NS	NS									
Zhong et al. [95]	NS		NS									PCoA	*
Zhou et al. [96]	NS				NS	NS		*				PCoA	*
Zhou et al. [97]	↓	↓		↓				*				PCoA	*
Zhou et al. [98]	NS				NS	NS	*	*				PCoA	*
Zhu et al. [99]	↓	↓			↓		NS	NS	NS		*	PCoA	NS *
Zhu et al. [100]	↑	↑					NS					PCoA	*
Zu et al. [101]	NS	NS	NS	NS								PCA	NS
↑	**1**	**3**	**1**	**1**									
↓/*	**12**	**7**	**4**	**2**	**1**	**3**	**9**	**11**	**8**	**2**	**3**		**30**
NS	**29**	**15**	**16**	**11**	**7**	**7**	**10**	**5**	**3**	**4**	**1**		**16**

↑ = increase; ↓ = decrease; NS = not significant; * = significant; ACE = abundance-based coverage estimator; AD = Aitchison distance; ASV = amplicon sequence variant; BC = Bray–Curtis; JD = Jaccard dissimilarity; MDS = multidimensional scaling; NMDS = non-metric multidimensional scaling; NR = not reported; NS = not specified; OPLS-DA = orthogonal partial least squares discriminant analysis; OTU = operational taxonomic unit; PCA = principal component analysis; PCoA = principal coordinates analysis; PERMANOVA = permutational multivariate analysis of variance; R = responders; TR = treatment resistant; UU = unweighted UniFrac; WU = weighted UniFrac; WCNA = weighted correlation network analysis.

**Table 5.** Alpha and beta diversity outcomes in individuals with anxiety disorders compared to controls.

Study ID	Alpha diversity						Beta diversity						
	Shannon	Chao1	Simpson	ACE	Observed OTU/ASV	PD	BC	WU	UU	AD	JD	Analysis	Finding
Chen et al. [49]	NS		NS	↓				*	*			PCoA PERMANOVA	*
Dong et al. [55]	↓	↓	↓	↓			NS					PCA	NS
Huang et al. [65]		↓											
Jiang et al. [66]	NS	↓	NS		↓				*			PCoA	*
Kim et al. [68]	↓ (Men) NS (Women)				↓ (Men) NS (Women)	↓ (Men) NS (Women)	*	*	*			PERMANOVA	*
Mason et al. [78]	NS							NS				PERMANOVA	NS
Wang et al. [82]		NS	NS	NS			*	*	*		*	PCoA	*
Zhu et al. [99]	↓	↓			↓		NS	NS	NS		*	PCoA	NS *
Zhu et al. [100]	↑	↑					NS					PCoA	*
↑	**1**	**1**											
↓/*	**3**	**4**	**1**	**2**	**3**	**1**	**2**	**3**	**4**		**1**		**6**
NS	**4**	**1**	**3**	**1**	**1**	**1**	**2**	**2**	**1**		**1**		**3**

↑ = Increase; ↓ = Decrease; NS = Not significant; * = Significant; ACE = Abundance-based Coverage Estimator; AD = Aitchison distance; ASV = Amplicon Sequence Variant; BC = Bray–Curtis; JD = Jaccard dissimilarity; MDS = Multidimensional Scaling; NMDS = Non-metric Multidimensional Scaling; NS = Not specified; OPLS-DA = Orthogonal Partial Least Squares Discriminant Analysis; OTU = Operational Taxonomic Unit; PCA = Principal Component Analysis; PCoA = Principal Coordinates Analysis; PERMANOVA = Permutational Multivariate Analysis of Variance; R = Responders; TR = Treatment resistant; UU = Unweighted UniFrac; WU = Weighted UniFrac; WCNA = Weighted Correlation Network Analysis.

Among the studies that investigated gut microbiota and depression (Table 4), 24 (~46%) studies reported no significant differences in alpha diversity between individuals with depression and healthy controls. In contrast, 9 (~17%) studies observed a significant reduction in alpha diversity while 2 (~4%) studies reported a significant increase in alpha diversity in individuals with depression. Measures of richness (e.g. observed species, Chao1, and ACE), which reflect the number of different taxa present, were calculated in 32 (~63%) studies. Measures of richness and evenness, such as Shannon and Simpson indices, which capture both the number of species and their relative abundance, were used in 44 (~86%) studies, while Faith's phylogenetic diversity, which considers evolutionary relationships between taxa, was reported in 10 (~20%) studies. Some studies reported a reduction in these indices (Table 4).

Hu et al.^64^ reported that persons with moderate MDD (HAMD-17 scale score 17–23) and severe MDD (HAMD-17 scale score > 24) had significantly reduced alpha diversity compared to healthy controls, whereas no significant difference was found in those with mild MDD (HAMD-17 scale score 8–16). Gao et al.^59^ observed a significant reduction in the Chao1, Pielou's evenness, and Simpson indices in treatment-resistant and responsive subgroups compared to controls, although Shannon, Faith's PD, observed species, and Good's coverage showed no significant differences. Li et al.^125^ also reported decreased Shannon's diversity, ACE, and Chao1 indices but a higher Simpson index in MDD patients. In contrast, Ling et al.^72^ observed significantly higher ACE, Chao1, and observed OTUs in children with MDD, with no significant differences in Shannon's diversity or Simpson indices.

Regarding anxiety disorders (Table 5), alpha diversity indices were significantly reduced in three studies^55^^,^^65^^,^^99^ and increased in one study^100^ in individuals with anxiety disorders compared to controls. Two studies^78^^,^^82^ observed no significant differences in alpha diversity measures (Shannon, Simpson, ACE, and observed OTUs/ASVs) in patients with anxiety disorder compared to controls. Richness-only measures, which quantify the number of distinct taxa present in a sample without considering their relative abundance or evenness (e.g. observed OTUs/ASVs, Chao1, ACE), were reported in six studies (Table 5). Richness and evenness indices like Shannon diversity index, were used in seven studies (Table 5), while Simpson's diversity index was used in four studies. One study^49^ reported that ACE was significantly reduced in the GAD group compared to healthy controls, with no differences in Shannon or Simpson indices. Notably, Kim et al.^68^ reported that in men with anxiety, alpha diversity measures were significantly lower compared to men without anxiety, while no significant differences were found in women with and without anxiety in the same study.

Beta diversity measures the differences in microbial community composition between samples.^126^ The abundance-based metric, Bray‒Curtis dissimilarity, was commonly reported and featured in 24 (~39%) studies, followed by weighted UniFrac and unweighted UniFrac, which are phylogeny-aware measures.^126^ The weighted version accounting for both evolutionary distance and relative abundance, while the unweighted version considering only presence/absence of taxa across phylogenetic trees.^126^ Weighted UniFrac and unweighted UniFrac were used in 19 (~31%) and 18 (~30%) studies, respectively. Jaccard dissimilarity, a presence/absence-based matrix, was used in 6 (~10%) studies. Aitchison distance, which is based on log-ratio transformations and is appropriate for compositional data, was used in four (~7%) studies. Distance matrices were visualized using ordination methods, including principal coordinate analysis (PCoA), principal component analysis (PCA), metric multidimensional scaling (MDS) or nonmetric multidimensional scaling (NMDS), and statistical testing was performed using tools, such as permutational multivariate analysis of variance (PERMANOVA), analysis of similarities (ANOSIM), orthogonal partial least squares discriminant analysis (OPLS-DA), partial least squares discriminant analysis (PLS-DA), or weighted gene coexpression network analysis (WGCNA)^127^ to determine the significance of group differences (Tables 3–5).

In PTSD, five of seven (~71%) studies assessed beta diversity, as shown in Table 3. There were no significant differences in beta diversity between cases and controls in three studies,^62^^,^^76^^,^^77^while two^58^^,^^86^ reported significant differences between cases and controls. In depression, beta diversity was examined in 48 (~92%) studies, as shown in Table 4. Of these, 28 (~58%) studies reported significant differences between cases and controls, most visualized via PCoA, while 16 (~33%) studies reported no significant group separation. However, Kim et al.^67^ found a significant difference in microbial composition between MDD patients and controls based on Bray–Curtis distance, while UniFrac and Jaccard distances did not reveal significant differences. Similarly, Gao et al.^59^ reported that PCoA based on Jaccard dissimilarity revealed significant separation between MDD responders and healthy controls but no significant difference between MDD treatment-resistant individuals and controls. For anxiety disorders (Table 5), five of the nine studies (~56%)^49^^,^^66^^,^^68^^,^^82^^,^^100^ reported significant differences between cases and controls, while three studies (~33%)^55^^,^^78^^,^^99^ found no significant separation.

### Taxonomic abundance

**Figure 3.**
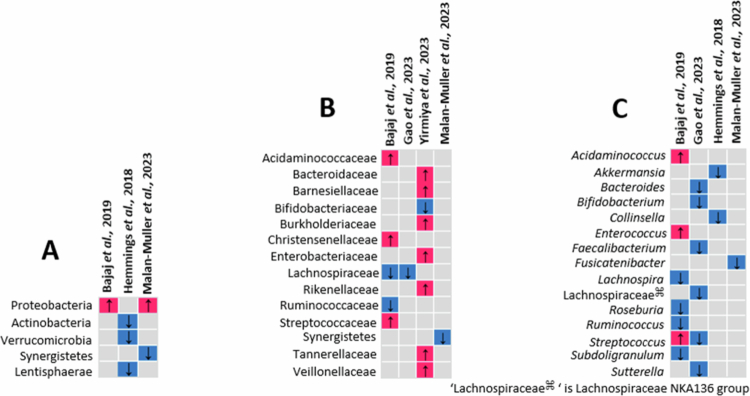
Summary of taxa relative abundance differences in persons with PTSD compared to controls. (A) Phylum level; (B) family level; and (C) genus level.

**Figure 4.**
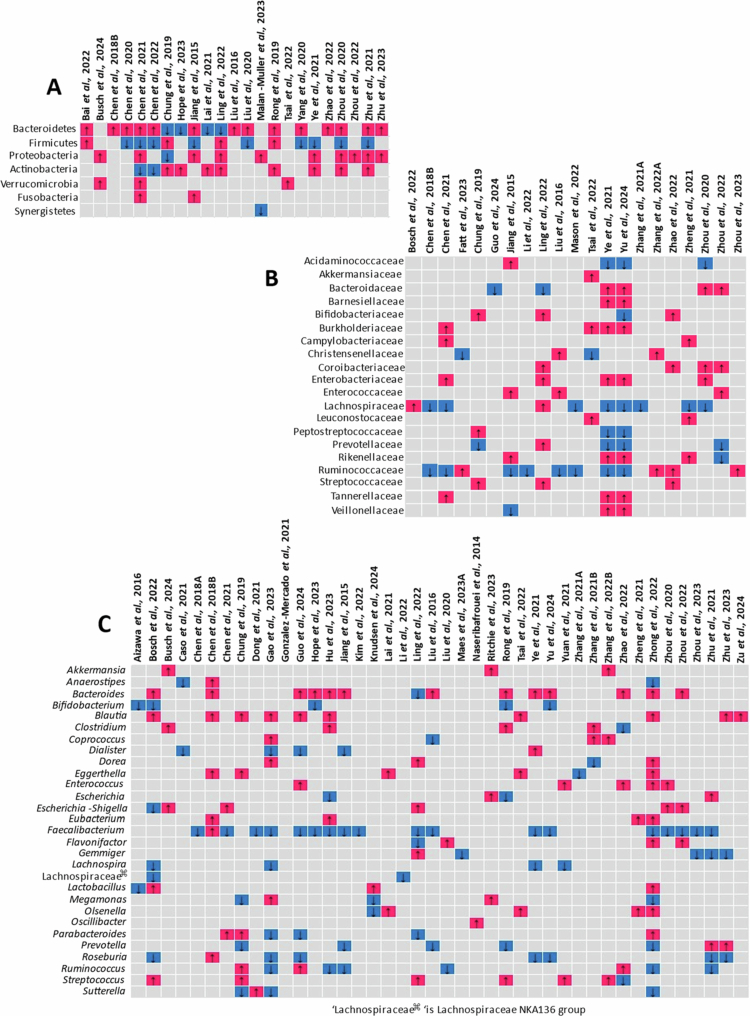
Summary of taxa abundance differences in persons with depression compared to controls. (A) Phylum level; (B) family level; and (C) genus level.

**Figure 5.**
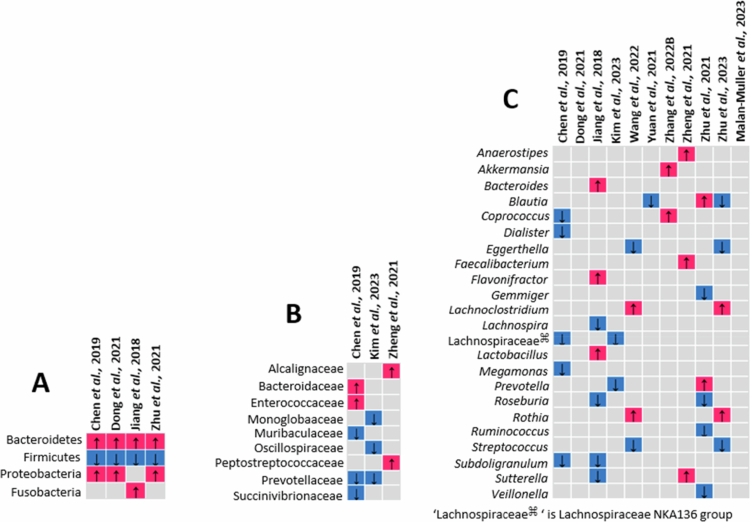
Summary of taxa abundance differences in persons with anxiety disorders compared to controls. (A) Phylum level; (B) Family level; and (C) Genus level.

Our synthesis revealed distinct yet overlapping patterns of gut microbiota alterations observed in PTSD (Figure 3A‒C), depression (Figure 4A‒C), and anxiety disorders (Figure 5A‒C). In studies focusing on PTSD, representative taxa at the phylum, family, and genus levels are summarized in Figure 3. Individuals with PTSD showed an increased relative abundance of taxa previously associated with proinflammatory activity, including the phylum Proteobacteria^43^^,^^77^ and families such as Acidaminococcaceae,^43^ Enterobacteriaceae,^86^ Streptococcaceae,^43^ and Veillonellaceae.^86^ Enrichment was also noted in short-chain fatty acid–producing families such as Bacteroidaceae and Barnesiellaceae. The following taxa were depleted: phyla, such as Actinobacteria^62^ and Verrucomicrobia^62^ and families, such as Lachnospiraceae,^43^^,^^58^ Ruminococcaceae,^43^ Rikenellaceae,^86^ and Bifidobacteriaceae^86^ (Figure 3A‒C). At the genus level, reductions in *Faecalibacterium,*^58^ *Roseburia,*^43^ *Subdoligranulum,*^43^ *Ruminococcus,*^43^ and *Lachnospira*^43^ suggest a depletion of key SCFA-producers, while increases in *Streptococcus*^43^^,^^58^ and *Enterococcus*^43^ were also observed (Figure 4A‒C).

Studies investigating the gut microbiota in individuals with depression (Figure 4A‒C) reported increased levels of the following phyla in patients compared to controls: Bacteroidetes, Proteobacteria, Actinobacteria, and Verrucomicrobia and a depletion of Firmicutes. At the family level, Lachnospiraceae, Ruminococcaceae, Acidaminococcaceae, and Prevotellaceae were decreased, while Bacteroidaceae, Rikenellaceae, Akkermansiaceae, Enterobacteriaceae, Enterococcaceae, Streptococcaceae, Burkholderiaceae, and Tannerellaceae were enriched in individuals with depression compared to controls. At the genus level, reductions in beneficial SCFA producers such as *Faecalibacterium, Bifidobacterium, Lachnospira, Ruminococcus*, *Lachnospiraceae* *NK4A136* group, *Roseburia*, and *Gemmiger* were observed in individuals with depression compared to controls, whereas there was enrichment of proinflammatory genera such as *Streptococcus*, *Eggerthella*, *Enterococcus*, *Escherichia-Shigella*, and *Flavonifractor*. Also enriched were SCFA-producing genera, including *Bacteroidetes, Coprococcus, Blautia, Clostridium, Dorea, Eubacterium,* and *Lactobacillus.* The gut microbial composition in depression varied with age,^50^ symptom severity,^44,63,64,92,95,95^ co-morbid symptoms,^29^^,^^61^^,^^80^^,^^92^^,^^94^^,^^99^^,^^128^ sex differences (microgenderome),^57^^,^^64^^,^^68^ subtypes,^72^^,^^81^ and treatment response categories.^28^^,^^46^^,^^59^^,^^61^^,^^66^

Regarding anxiety disorders, the representative taxa findings are summarized in Figure 5A‒C. At the phylum level, Bacteroidetes and Proteobacteria were enriched, while Firmicutes were depleted in individuals with anxiety disorders compared to controls. At the family level, Bacteroidaceae,^49^ Enterococcaceae,^49^ and Peptostreptococcaceae^94^ were enriched, while Prevotellaceae,^49^^,^^68^ Muribaculaceae,^49^ and Oscillospiraceae^68^ were depleted. Genus-level differences included depletion of generally beneficial taxa such as *Blautia,*^87^^,^^100^ *Coprococcus,*^49^ *Dialister,*^49^ *Subdoligranulum,*^49^^,^^66^ *Ruminococcus,*^99^ and *Roseburia,*^66^^,^^99^ with increases in genera linked to inflammation, such as *Flavonifractor*^66^ and *Rothia,*^82^^,^^100^ SCFA-producing taxa such as *Lachnoclostridium*^82^^,^^100^ and *Faecalibacterium,*^94^ and *Akkermansia,*^92^ an anti-inflammatory taxa. In anxiety disorders, sex-specific alterations in the gut microbiota was observed (e.g. males with anxiety disorders showed reduced *Monoglobales*, *Monoglobaceae*, and *Lachnospiraceae NK4A136*, while females with anxiety disorders presented with lower abundances of *Prevotellaceae* and *Prevotella*). Interventions further modulated these patterns. Mindfulness-based cognitive therapy (MBCT)^82^ resulted in the enrichment of *Streptococcus*, *Blautia*, *Romboutsia*, and *Eggerthella* and the depletion of *Lachnoclostridium*, *Rothia*, *Lachnospiraceae UCG010*, *Faecalibacterium*, *Coprococcus*, and *Eubacterium eligens*^73^ in the high-trait anxiety group [those who scored ≥50 points on the trait anxiety subscale of the State-Trait Anxiety Inventory (STAI)].

## Discussion

Understanding the relationship between the gut microbiota and mental health is a rapidly expanding area within the field of psychiatry. However, despite the growing number of studies, the field remains relatively new and fragmented, with many investigations conducted in silos. Current research is limited by a lack of standardization in study design, including differences in sample collection, sequencing platforms, and analytical pipelines. Moreover, microbial composition is shaped not only by mental health status but also by host- and environment-related factors such as age,^129^ sex,^130^ disease severity,^131^ and medication, all of which can act as important confounders. These complexities make cross-study comparisons challenging. Our synthesis of the current literature revealed variability in methodological choices, from the timing and handling of fecal sample collection to the use of bioinformatic pipelines, which may contribute to heterogeneity in reported microbial associations.

### Alpha and beta diversity

Across studies, alpha diversity findings in PTSD, depression, and anxiety disorders were inconsistent, with some reporting reductions in richness and evenness indices in cases versus controls, while others reporting no significant differences (Tables 3–5). Several studies reported reduced alpha diversity reported reduced alpha diversity in indices such as Shannon,^28^^,^^43-45^^,^^55^^,^^58^^,^^63^^,^^64^^,^^67^^,^^68^^,^^71^^,^^73^^,^^84^^,^^86^^,^^87^^,^^96^^,^^98^^,^^99^ Chao1,^40^^,^^55^^,^^58^^,^^59^^,^^61^^,^^63^^,^^65^^,^^66^^,^^71^^,^^84^^,^^96^^,^^99^^,^^100^ Simpson,^45^^,^^55^^,^^58^^,^^61^^,^^64^^,^^77^ ACE,^50^^,^^55^^,^^58^^,^^71^^,^^96^ Observed OUT/ASV,^58^^,^^68^^,^^99^^,^^100^ and Faith's PD^43^^,^^62^^,^^84^^,^^89^—in individuals with PTSD, depression, and anxiety compared to controls (Tables 3–5). These reductions were associated with symptom severity,^63^^,^^67^ treatment response,^59^ and comorbid conditions such as anxiety trait^77^ and anorexia nervosa.^61^ A reduced alpha diversity may reflect an altered gut microbiome profile, impaired microbial resilience, and reduced functional redundancy, which are thought to contribute to inflammation, altered neurotransmitter metabolism, and dysregulated gut‒brain communication.^28^^,^^35^ However, not all studies aligned: one study^66^ reported increased Shannon diversity in active-MDD compared to controls, while another study^72^ observed higher richness indices (ACE, Chao1, observed OTUs) in children with MDD versus controls. Sex-based differences were also reported for alpha diversity indices. Kim et al.^68^ reported lower Shannon diversity, Faith's PD, and observed ASVs in men with anxiety compared to male controls, whereas no significant differences emerged among women. These findings suggest that biological factors, such as disease stage, age-related microbiome profile, host genetics, and diet, along with methodological and population-based differences, may influence diversity outcomes.

Regarding beta diversity, about half of the studies reported significant diagnostic group differences. The differences in beta diversity outcomes across studies reflect underlying methodological and biological variability. For example, distinct microbial clustering was associated with several factors, including symptom severity,^59^^,^^68^^,^^95^ comorbid conditions,^87^ sex differences,^48^^,^^68^ and treatment response categories.^59^ For instance, microbial composition differed between moderate and severe MDD patients^64^^,^^95^ and between MDD patients with and without comorbid anorexia.^61^ Intervention studies provided more insight into the dynamic nature of gut microbial diversity. Wang et al.^82^ demonstrated that individuals with trait anxiety initially had significantly altered beta diversity compared to controls, but their microbiome composition gradually shifted closer to that of healthy individuals following an 8-week mindfulness-based cognitive therapy. Similarly, Zhang et al.^89^ reported only minor differences in beta diversity between baseline and postintervention time points in both the probiotic and placebo groups among participants with depression, indicating that the microbial community structure may exhibit resilience over time. Despite these findings, inconsistencies remain. While many studies reported altered beta diversity, some studies (Tables 3–5) did not observe significant differences. The observed discrepancies could stem from differences in study design, including sample sizes, sequencing techniques, geographical factors, age, and sex.

### Gut microbiome alterations

Figures 3‒5(A‒C) demonstrate taxonomic differences in the gut microbial composition between individuals with PTSD, depression, anxiety disorders, and controls. However, most of the included studies relied on 16S rRNA gene amplicon sequencing and did not directly assess microbial functional pathways (e.g. metagenomics, metabolomics, or transcriptomics). The mechanistic interpretations discussed below are grounded in established experimental and translational literature and are presented as biologically plausible pathways supported by prior evidence. Across PTSD, depression, and anxiety disorders, the only consistently reported taxonomic alteration was an increase in Proteobacteria. Reductions in key short-chain fatty acid (SCFA)–producing genera were disorder-specific rather than consistent: Faecalibacterium was decreased in depression and PTSD but increased in anxiety disorders, while Subdoligranulum was not observed in depression.

### Gut barrier–immune activation axis

Chronic low-grade inflammation and neuroinflammation are increasingly recognized as risk factors for stress-related psychiatric disorders.^132-137^ In our synthesis, enrichment of Proteobacteria was observed across PTSD^43^^,^^77^ (Figure 3A), depression^28^^,^^45^^,^^51^^,^^72^^,^^84^^,^^96^^,^^97^^,^^99^^,^^100^ (Figure 4A), and anxiety disorders^49^^,^^99^^,^^100^ (Figure 5A). Proteobacteria is regarded as a marker of gut dysbiosis^138^^,^^139^ and major source of lipopolysaccharide (LPS).^45^^,^^49^ LPS are components of the outer membrane of Gram-negative bacteria (e.g. *Escherichia* and *Shigella*)^71^^,^^75^. LPS activates Toll-like receptor 4 (TLR4) signaling, promoting systemic cytokine release (e.g. IL-1β, IL-6, TNF,) and sustaining chronic low-grade inflammation.^46^^,^^140^^,^^141^ The enrichment of LPS-associated taxa is consistent with prior mechanistic literature linking gut-derived inflammatory signaling to mood and anxiety disorders.^33^^,^^46^^,^^71^^,^^73^^,^^81^^,^^142-146^ While the reviewed studies did not measure circulating LPS profiles in all cases, the enrichment of LPS-producing taxa is consistent with prior mechanistic evidence linking gut-derived inflammation to neuropsychiatric vulnerability.

Conversely, depletion of SCFA-producing families such as Lachnospiraceae and Ruminococcaceae, and genera including *Faecalibacterium* and *Roseburia*^33^^,^^46^^,^^71^^,^^73^^,^^81^ may suggest reduced microbial support for epithelial integrity. SCFAs (e.g. butyrate, acetate, and propionate) are gut microbial metabolites from fiber fermentation.^147^ Butyrate, for example, is known to (1) support gut barrier integrity by stimulating the production of mucus and tight junction proteins to increase the integrity of the intestinal epithelium; (2) regulate immune responses; (3) influence neuroplasticity through histone deacetylase inhibition; (4) modulate microglia; and (5) exhibit anti-inflammatory properties.^148-150^ It also directly influences CNS function by modulating the expression of brain-derived neurotrophic factor.^148^^,^^149^ Reduced abundance of these SCFA-producing taxa is therefore biologically plausible within models proposing impaired epithelial support and altered immune signaling.

Several proinflammatory bacterial families and genera were enriched across these disorders (Figures 3B, 4B, and 5B). Families such as Enterobacteriaceae, Enterococcaceae, Streptococcaceae, and Burkholderiaceae and genera such as *Enterococcus, Escherichia-Shigella, Ruminococcus gnavus,* and *Streptococcus*in PTSD, depression, and anxiety disorders were enriched compared to controls. *Enterococcus, Escherichia-Shigella*, and *Ruminococcus gnavus* have been linked to increased intestinal permeability, oxidative stress, and systemic inflammation, all of which contribute to mood disturbances. *Enterococcus* species are known for their opportunistic nature and can exacerbate gut dysbiosis, increase levels of proinflammatory cytokines, and compromise gut barrier function.^151^ Increased levels of *Escherichia-Shigella* correlate with elevated LPS and inflammatory cytokines in depression.^152^ Proinflammatory gut microbe linked to inflammatory bowel disease, was increased in both PTSD^88^ and anxiety disorders.^66^ Streptococcaceae, particularly the *Streptococcus* species, may influence these psychiatric disorders by affecting blood‒brain barrier permeability and modulating neuroactive compounds such as histamine and serotonin precursors, which are key to mood regulation.^60^ Put together, these patterns support a gut barrier–immune axis model in which dysbiosis promotes epithelial compromise and systemic inflammation.

### Ecological context of immune dysregulation

A number of complementary ecological hypotheses propose that reduced exposure to diverse environmental microorganisms in modern urban societies contributes to impaired immunoregulation and chronic low-grade inflammation.^153^^,^^154^ These include the hygiene hypothesis,^155^ the Old Friends hypothesis,^156-159^ the biodiversity hypothesis,^160^ the disappearing microbiota hypothesis,^161^ and the industrialized microbiota hypothesis^162^ (Table 6). Although differing in emphasis, these frameworks converge on the concept that diminished microbial exposure and diversity may impair regulatory immune pathways and increase vulnerability to inflammatory disorders.

**Table 6.** Ecological hypotheses linking microbial exposure to immune dysregulation and psychiatric risk.

Hypothesis	Core concept	Proposed immunological mechanism	Relevance to psychiatric disorders
Hygiene hypothesis [155]	Reduced early-life exposure to microbes due to improved sanitation and smaller family size	Impaired immune maturation; imbalance in Th1/Th2 responses; increased inflammatory susceptibility	Chronic low-grade inflammation implicated in depression, anxiety, and stress-related disorders
Old Friends hypothesis [156]	Loss of co-evolved, immunoregulatory microorganisms that historically shaped human immune tolerance	Reduced induction of regulatory T cells (Tregs); impaired immunoregulation; exaggerated inflammatory responses	Increased vulnerability to stress-induced immune activation and neuroinflammation
Biodiversity hypothesis [160]	Reduced environmental biodiversity leads to diminished host microbial diversity	Lower microbial richness and resilience; impaired immune tolerance	Greater susceptibility to dysbiosis and inflammatory disorders, including psychiatric conditions
Disappearing microbiota hypothesis [161]	Progressive extinction of ancestral commensal microbes due to antibiotics, cesarean delivery, diet shifts	Loss of functional redundancy and metabolic capacity within the gut ecosystem	Reduced resilience to inflammatory and stress-related perturbations
Industrialized microbiota hypothesis [162]	Westernized lifestyle reshapes microbial composition across generations	Diet-driven loss of fiber-fermenting taxa; altered SCFA production; reduced microbial diversity	Microbiome shifts observed in depression, PTSD, and anxiety may reflect industrialized ecological patterns

### The complex landscape of short-chain fatty acid production

Interestingly, some studies reported enrichment of certain SCFA-associated taxa, for example, Bacteroidetes, was elevated in depression^28^^,^^29^^,^^42^^,^^50-52^^,^^54^^,^^70^^,^^72^^,^^73^^,^^85^^,^^93^^,^^96^^,^^99^^,^^100^ and anxiety disorders.^49^^,^^66^^,^^99^^,^^100^ Bacteroidaceae, Barnesiellaceae, Rikenellaceae, and Tannerellaceae were enriched across studies of PTSD,^86^ depression,^28^^,^^84^^,^^85^^,^^94^^,^^96^^,^^97^ and anxiety disorders.^49^ Members of these families are associated with the production of SCFAs and other metabolites involved in gut–brain signaling. Metabolic capacity may vary across species and strains within the same taxonomic group. Hence, the enrichment at the family level does not necessarily reflect uniform functional output.

### Neurotransmitter modulation

The current review is consistent with the hypothesis that microbiome-driven modulation of neurotransmitter, availability, and signaling constitutes a key mechanistic link between the gut microbiome and vulnerability to stress-related psychiatric disorders. Among the neurotransmitter pathways implicated, this model is most extensively developed for tryptophan metabolism, which is tightly regulated by host–microbial interactions. Gut microbiota influence the partitioning of tryptophan between the serotonergic pathway, which supports central serotonin synthesis, and the kynurenine pathway, which generates neuroactive metabolites with immunomodulatory and neurotoxic or neuroprotective properties.^163^^,^^164^ Dysregulation of this metabolic balance, often driven by inflammation and microbial compositional shifts, has been implicated in altered stress responsivity, affective dysregulation, and the pathophysiology of depression and related disorders.^165^ These observations support a model in which microbiome-dependent control of neurotransmitter precursor metabolism contributes to individual differences in stress susceptibility and psychiatric risk.

### Age and microbiome patterns

The gut microbiota evolves across the lifespan, with distinct microbial profiles observed in childhood, and adulthood.^50^^,^^166^ During the neonatal period, the dominant taxa are Actinobacteria (particularly *Bifidobacterium*) and Proteobacteria, while the relative abundance of Firmicutes and Bacteroidetes begins to increase between three and 6 months of age.^50^^,^^166^ Bacteroidetes and Firmicutes are the dominant bacterial taxa in adults.^50^^,^^167^ The Firmicutes/Bacteroidetes ratio is a broad ecological index of gut community composition, which reflects the relative abundance of two major bacterial phyla in the gut microbiome and is widely used as a marker of gut dysbiosis.^168^ A reduced abundance of Bacteroidetes has been documented in older adults compared to younger individuals.^50^^,^^169^ Microbiota disturbances in early life, defined as the prenatal period, infancy, and early childhood,^170^ can have lasting effects on mental health. This developmental window is critical for physical, cognitive, emotional, and social growth, laying the foundation for lifelong physical and behavioral health. Disruptions during this period, whether caused by stress, antibiotics, or diet,^171^^,^^172^ can impair the gut–brain axis, leading to HPA axis dysregulation, neuroinflammation, altered neurotransmitter production, and immune dysfunction.^35^^,^^50^

Microbial communities evolve with age due to dietary changes, hormonal shifts, and cumulative environmental exposures, altering their functional impact on the brain.^173^ Chen et al.^50^ demonstrated age-specific differential changes in the gut microbiome in people with MDD. Lachnospiraceae, Ruminococcaceae, and Peptostreptococcaceae families were enriched in young persons with MDD compared to middle-aged persons with MDD, while Prevotellaceae, Veillonellaceae, and Acidaminocaccaceae families were enriched in the middle-aged MDD group compared to the young MDD group. These findings underscore the importance of considering age as a key factor when investigating gut microbiome alterations in depression as well as PTSD and anxiety disorders. The differential enrichment of bacterial families in young versus middle-aged persons with depression reflects age-specific interactions between the gut microbiome, host physiology, and environmental factors, leading to distinct mechanistic pathways driving the disorder. This reinforces the potential for personalized microbiome-based interventions to improve outcomes in individuals with PTSD, depression, and anxiety disorders.

Most microbiome studies on these neuropsychiatric disorders use broad age ranges (e.g. 18–65 y) to investigate gut‒brain interactions. While this approach allows for a larger sample size and statistical power, it overlooks age-specific microbial patterns. This was demonstrated by Chen et al.^50^, who noted distinct gut microbiome differences between young and middle-aged people with MDD, suggesting that treating 18–65-y-olds as a single group may mask meaningful biological variations. For example, young adults (18–30 y) may experience PTSD, depression, and anxiety disorders within the context of academic stress, social pressures, and early-life trauma, whereas middle-aged individuals (31–45 y) may be more affected by chronic stress, metabolic disorders, and neurodegenerative changes.^174^

### Stratifying microbiome profiles by sex and disease severity

Sex hormones shape gut microbiota composition,^48^^,^^175-178^ contributing to differences in disease susceptibility, symptom expression, and treatment response.^57^^,^^179^ The concept of the microgenderome suggests that sex hormones modulate the gut microbiome, leading to sex-specific host‒microbiota interactions.^48^^,^^175-178^ Sex differences in the gut microbiome have been demonstrated in both preclinical studies and human studies.^48^^,^^57^^,^^167^^,^^176-181^ In this review, sex-specific differences in the gut microbiota composition were noted in a few studies.^48^^,^^57^^,^^64^^,^^68^ Hu et al.^64^ reported that *Ruminococcus* was enriched only in males with MDD, suggesting a potential sex-linked microbial signature. Ganci et al. ^57^ had documented a higher relative abundance of *Ruminococcus gnavus* in depressed males compared to females. Kim et al.^68^ reported a reduced relative abundance of Lachnospiraceae_NK4A136 group in males with anxiety disorder and the depletion of *Prevotella* in females with anxiety disorders. Chen et al.^48^ also documented distinct microbial compositions in females with MDD compared to males with MDD. They reported enrichment of Actinobacteria*, Actinomyces, Bifidobacterium, Atopobium, Eggerthella, Roseburia,* and *Faecalibacterium* in females with MDD compared to males with MDD, while female healthy controls compared to male healthy controls exhibited higher abundances of *Howardella, Sutterella,* and *Pyramidobacter*. In contrast, males with MDD compared to females with MDD showed higher relative abundances of Bacteroidetes, *Bacteroidia*, *Bacteroides*, *Veillonella*, and *Erysipelotrichaceae incertae sedis*, whereas male healthy controls had an enrichment of *Anaerovorax, Gordonibacter,* and *Pyramidobacter*compared to female healthy controls.

These findings suggest that the gut microbiota's role in PTSD, depression, and anxiety disorders may be modulated by sex; however, most of the studies in this review did not incorporate sex-stratified analyses, limiting our understanding of the biological mechanisms underlying sex-specific vulnerabilities. Given that these disorders may be influenced by hormonal fluctuations,^48^^,^^175-178^ neglecting sex as a biological variable may contribute to inconsistent findings on microbiome alterations. Importantly, the absence of sex-stratified data hampers efforts to inform precision medicine approaches, where microbiota-based interventions could be tailored differently for males and females.

The synthesis of results also shows that the gut microbiota composition in depression may be influenced by disease state (active vs. remitted) and subtype (typical vs. atypical). This highlights the need for a more nuanced approach to studying microbiota alterations in PTSD, depression and anxiety disorders. Jiang et al.^28^ demonstrated that both active (A-MDD) and remitted MDD (R-MDD) share common microbial signatures compared to control (e.g. enrichment in Bacteroidetes, Proteobacteria, and Enterobacteriaceae, and depletion of Firmicutes, Lachnospiraceae, Ruminococcaceae, and Veillonellaceae) compared to healthy controls. However, distinct differences were observed between the two groups, with A-MDD characterized by an increased presence of *Alistipes*, *Blautia*, *Clostridium XIX*, and *Roseburia*, whereas R-MDD displayed an enrichment of *Clostridium sensu stricto* and *Bacteroides* and the depletion of key beneficial taxa such as *Faecalibacterium* and *Ruminococcus*. These findings suggest that microbiota imbalances may persist even after remission, potentially contributing to the risk of relapse. The study by Caso et al.^46^ identified elevated levels of *Bilophila* and *Alistipes* in both acute and remitted MDD patients compared to controls. However, the depletion of *Anaerostipes* and *Dialister* was more pronounced in acute cases. Interestingly, the persistence of *Bilophila* enrichment in remitted MDD patients could indicate lingering dysbiosis that may influence mental health outcomes.

Busch et al.^45^ provided insight into microbiota differences between MDD subtypes, revealing that atypical MDD was associated with an enrichment of Verrucomicrobia, particularly *Akkermansia*, while both atypical and typical MDD patients exhibited increased Proteobacteria and *Bifidobacterium* levels. However, specific microbial signatures distinguished typical MDD, with *Escherichia/Shigella* and Clostridia being more abundant, whereas *Phascolarctobacterium* and *Romboutsia* were prevalent in healthy controls. Conversely, Jiang et al.^66^ reported that the depletion of *Bacteroides* spp. in remissive-GAD compared to active-GAD and the enrichment of *Faecalibacterium*, *Eubacterium rectale*, and *Sutterella* in remissive-GAD compared to the active-GAD. The presence of distinct microbial patterns between the MDD and GAD subtypes suggests that the gut microbiota composition may not only reflect disease status but also contribute to the heterogeneity observed in symptom presentation and treatment response. These findings underscore the complexity of the gut microbiome in PTSD, depression, and anxiety disorders and hence the need to stratify based on disease stage and subtype in microbiota research.

The findings of Zhang et al.^92^, Zhong et al.^95^, and Hu et al.^64^ suggest that microbial alterations are associated with symptom severity. Zhang et al.^92^ reported that *Akkermansia* and *Phascolarctobacterium* were enriched in individuals with severe depressive symptoms, while severe anxiety symptoms were associated with increased relative abundances of *Akkermansia, Coprococcus,* and *Streptococcus* Compared to persons with less severe symptoms. These findings suggest that specific microbial taxa may be linked to the severity of depressive and anxiety symptoms. Zhong et al.^95^ reported distinct gut microbiota patterns across depressive symptom severity levels compared to control: moderate depression (Hamilton Depression Rating Scale, HDRS < 25) group showed depletion of *Faecalibacterium* and *Prevotella* and enrichment of *Dorea, Alistipes, Blautia*, and *Parabacteroides* compared to controls, while the severe depression (HDRS ≥ 25) group had increased abundance of *Eggerthella, Bacteroides, Megasphaera*, and *Bilophila*, and reduced *Lactococcus, Sutterella*, and *Faecalibacterium* compared to controls, mild and severe depression groups. Taxa such as *Collinsella, Eggerthella, Alistipes, Faecalibacterium,* and *Flavonifractor* were altered in both the moderate and severe depression groups compared to controls, with a predictive model based on the areas under the curve (AUCs) of 0.786, which suggests that the pattern of abundance carries diagnostic signal strong enough to classify individuals with reasonable accuracy, highlighting their potential as microbial biomarkers of depression severity. However, this is an associative model, not proof of causality. Hu et al.^64^ found a higher prevalence of *Bacteroides*-dominated enterotypes in moderate (HAMD-17 score 17–23) and severe (HAMD-17 score ≥ 24) depression patients. Using 37 bacterial taxa as biomarkers, they built a predictive model that distinguished depression severity (mild vs moderate, mild vs. severe, and moderate vs. severe) with excellent diagnostic accuracy (AUC: 0.992–0.998). These findings demonstrate the need to consider disease severity when analyzing gut microbiome alterations in PTSD, depression, and anxiety disorders. Most of the studies (*n* = 61, 95.3%) in this review treated these disorders as homogeneous conditions, failing to consider the potential variability in microbial signatures across different severity levels. This may have obvious implications for the interpretation of results, the identification of reliable biomarkers, and the development of microbiota-targeted interventions.

### Gut microbial signatures vary by treatment status and response in anxiety and depression

Five studies reported that gut microbial profiles diverged in treatment-naïve,^66^^,^^73^ medicated,^59^^,^^73^^,^^100^ and treatment-resistant^59^ groups, indicating that psychotropic exposure and clinical response may reshape gut communities. In anxiety disorder, treatment-naïve patients showed enrichment of *Bacteroides*, *Escherichia–Shigella*, *Ruminococcus gnavus*, *Lactobacillus*, and *Fusobacterium*, alongside depletion of butyrate-producing taxa (*Faecalibacterium*, *Eubacterium rectale*, *Roseburia*, *Lachnospira*), consistent with a more proinflammatory, barrier-perturbing milieu.^66^

In MDD, medicated patients, relative to unmedicated patients and controls, had broad reductions in Firmicutes (notably Clostridia/Clostridiales, Ruminococcaceae, Faecalibacterium, *[Eubacterium] coprostanoligenes group*) and enrichment of Bacteroidetes/Bacteroidia/Bacteroidales; Enterococcus/Enterococcaceae were detected chiefly in the medicated group.^73^ Unmedicated patients were enriched for Christensenellaceae (including the R-7 group), Barnesiellaceae/Barnesiella, and an uncultured Clostridiales (BB60 family), with Fusicatenibacter depleted.^73^ Together, these shifts imply that medication exposure can reduce key SCFA producers and favor taxa adapted to altered bile-acid/redox states, with downstream effects on inflammatory and metabolic tone.

Among SSRI-treated MDD participants, responders (vs nonresponders and controls) were enriched in SCFA-linked genera (*Blautia*, *Bifidobacterium*, *Coprococcus*, *Collinsella*, *Dorea*), whereas treatment-resistant patients showed enrichment of *Megamonas.*^59^ A microbiome-based model distinguished SSRI responders with AUC = 0.931 (sensitivity: 0.879; specificity: 0.920),^59^ underscoring predictive potential for treatment outcomes. Non-pharmacologic and psychobiotic interventions: In high trait-anxiety, strong responders to mindfulness-based cognitive therapy exhibited enrichment of *Subdoligranulum*, *Eubacterium group*, and *Ruminococcus.*^82^ In students with depression/anxiety, *Lactobacillus plantarum* JYLP-326 shifted composition toward Prevotella and Bifidobacterium, with reductions in Bacteroides and Roseburia.^100^

Treatment-naïve anxiety and depression is thus marked by loss of butyrate producers and gain of pathobionts; medication and behavioral/probiotic interventions each impose distinct, often directionally consistent shifts, and responder vs non-responder profiles diverge—pointing to candidate microbial biomarkers and mechanisms (e.g. SCFA availability, bile-acid signaling, mucosal inflammation) that may shape both efficacy and side-effect profiles.^59,66,73,73,100^ Future studies should stratify by drug class, dose, and duration and use longitudinal pre/post designs with rigorous control for diet, BMI, smoking, and co-medications.

### Methodological quality and sources of bias

Gut microbiome alterations were reported in PTSD, depression, and anxiety; however, the strength and reliability of these findings are limited by methodological differences. There were differences in study design, participant demographics, sample sizes, and geographic representation, with a predominance of studies from China and limited data from Europe, Africa, and other regions (Tables 1 and 2(A–D), Supplementary Tables 1 and 2 and Figure 2). Geographic location and the sociocultural, dietary, and environmental factors it reflects have been shown to influence gut microbiome composition.^182^ Geographic region was found to explain the largest proportion of the overall variance in gut microbiota composition.^183^ The predominance of Chinese cohorts in the literature may therefore limit the generalizability of taxa-level associations to other cultural and dietary contexts and could partly account for inconsistencies observed across studies. In addition, most of the included studies were cross-sectional, limiting causal interpretations. Depression was the most studied disorder (Tables 1 and 2(A–D), Supplementary Tables 1 and 2, and Figure 2), while PTSD and anxiety were underrepresented, making it difficult to draw broad conclusions about microbiota changes across these mental health conditions.

Comorbid conditions and uncontrolled confounders represent important considerations in interpreting gut microbiome alterations in PTSD, depression, and anxiety.^2^^,^^184^ Several included cohorts exhibit overlapping psychiatric or somatic symptoms; for example, sleep disturbance and cognitive impairment in late-life depression,^81^ insomnia,^57^^,^^63^^,^^81^^,^^90^^,^^91^^,^^99^ and participants with cooccurring depression and anxiety^38^^,^^47^^,^^52-57^^,^^62^^,^^76-78^^,^^80^^,^^82^^,^^87^^,^^89^^,^^90^^,^^92^^,^^97^^,^^99^ (Table 1 and Supplementary Table 1). Such overlapping phenotypes may blur disorder-specific microbial signatures, complicating efforts to identify robust, cross-study associations. Furthermore, many studies did not control for psychotropic medications. Antidepressants, anxiolytics, and antipsychotics are known to alter the gut microbial composition, yet medication use was inconsistently reported and rarely incorporated into analytical models.^59^ As a result, the observed microbial differences may reflect treatment effects rather than disease-specific signatures. Key lifestyle and metabolic variables, including diet, body mass index, smoking, alcohol use, and physical activity, were similarly underreported or uncontrolled, despite their well-established influence on gut microbial structure and function. Collectively, these factors underscore the need for cautious interpretation of reported microbial patterns and highlight the importance of rigorous study design, comprehensive phenotyping, and covariate adjustment in future microbiome–psychiatric research. There were methodological inconsistencies in sample collection, DNA extraction, sequencing regions, and data processing. Variability in fecal sample handling, DNA extraction protocols, and targeted 16S rRNA gene regions likely contributed to differences in microbial profiles^185-187^ (Table 2(A–D), Supplementary Table 2 and Figure 2). While most studies have used modern analytical pipelines such as QIIME2^114^ and DADA2^113^ (Table 2(A–D), Supplementary Table 2 and Figure 2), differences in versions, parameters, and reference databases have reduced comparability. Different software versions, analysis settings, and reference databases can alter sequence processing and taxonomic assignment, creating technical variability that limits cross-study comparability.^188^ A lack of standardization in sample collection, DNA extraction, sequencing regions, and data processing hinders reproducibility and limits the synthesis of findings across studies. Furthermore, few studies have accounted for psychiatric comorbidities, which can influence the gut microbiota independently. Future research will benefit from standardized protocols, better reporting practices and greater inclusion of diverse populations and psychiatric phenotypes.

### Recommendation

Understanding the microbiome–gut–brain axis in PTSD, depression, and anxiety disorders holds immense promise; however, inconsistent methodologies and interpretive gaps limit the translational impact of available microbiome research in these disorders. To increase the reproducibility, interpretability, and clinical relevance of microbiome research in PTSD, depression, and anxiety disorders, future studies should consider adopting harmonized best practices that span study design, data generation, and analysis. Diagnostic consistency is important^189^; hence, future studies should employ standardized criteria and incorporate validated symptom severity scales to ensure accurate phenotyping and comparability across cohorts. Key confounding factors such as diet, BMI, Bristol stool scale, age, sex, psychotropic medication, and comorbid psychiatric conditions should be accounted for in both study design and statistical modeling, given their profound influence on gut microbial composition.^190^ Longitudinal, interventional, or responder/nonresponder designs should be prioritized over purely cross-sectional approaches to enable causal inference and temporal mapping of microbial shifts relative to disease onset, treatment, or symptom severity.

**Figure 6.**
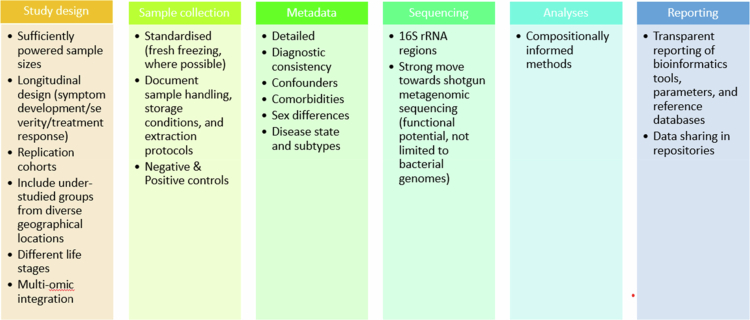
Best practices for microbiome research.

Sample collection and handling protocols should also be standardized: stool collection should use validated methods with immediate freezing at –80 °C or stabilization buffers, and metadata must document exact sample collection times, storage conditions, and extraction protocols (Figure 6). Batch effects, contamination, and DNA yield variability should be addressed by including negative and positive controls and by performing technical replicates where feasible. Studies should strive for adequate sample sizes and geographic, racial, and dietary diversity to ensure generalizability and uncover population-specific microbial signatures.

Multiomic integration is necessary; 16S rRNA gene sequencing or shotgun metagenomics with fecal metabolomics, host metabolomics and transcriptomics, immune profiling, and brain imaging can reveal functional and mechanistic insights into microbiome-gut–brain axis communication (Figure 2). Computational analysis must move beyond alpha and beta diversity, incorporating compositionally aware methods, feature selection algorithms, and systems biology tools (e.g. WGCNA). Moreover, robust cross-validation, replication in independent cohorts, and transparent reporting of bioinformatics tools, parameters, and reference databases are necessary to support reproducibility.

We propose a roadmap that emphasizes: (1) harmonization of protocols; (2) improved metadata annotation; (3) greater inclusion of underrepresented populations, particularly from LMICs; (4) collaborative data sharing through open-access platforms; and (5) translational follow-up through psychobiotic trials, fecal microbiota transplantation, and microbial target validation in gnotobiotic or humanized models. Such efforts will collectively strengthen the evidence base and accelerate the translation of microbiome insights into actionable diagnostics and therapeutic strategies in mental health.

## Conclusion

Converging evidence on the gut microbiome in PTSD, depression, and anxiety disorders points to a common trend of dysbiosis, characterized by the enrichment of proinflammatory bacterial taxa (e.g. Proteobacteria, Burkholderiaceae, Enterobacteriaceae, Streptococcaceae, *Enterococcus, and Escherichia–Shigella*) and a depletion of beneficial gut bacteria such as Firmicutes, Lachnospiraceae, and Ruminococcaceae, *Faecalibacterium, Roseburia,* and *Subdoligranulum*. However, despite methodological differences, certain taxa, such as *Bacteroides, Blautia, Bifidobacterium*, and *Faecalibacterium*, were consistently altered in individuals with depression. Notably, most data come from high-income countries, with limited representation from LMICs and African populations. Future research should prioritize longitudinal studies, methodological standardization, and the inclusion of diverse populations. While current evidence does not yet support clinical translation, the gut microbiome remains a promising avenue for understanding neuropsychiatric disorders and identifying potential biomarkers or therapeutic targets.

## Supplementary Material

Supplementary materials KGMR_A_2654224_SM1751.docx

## Data Availability

The data that support the findings of this study are derived from publicly available sources. All data analyzed during this study are included in this published article and its supplementary information files.
