# Supplementary material for: The role of gut microbiome in the pathophysiology of PTSD, depression, and anxiety disorders
Source: Gut Microbes Rep. 2026 Apr 14;3(1):2654224. doi: 10.1080/29933935.2026.2654224 (PMC13085948; doi:10.1080/29933935.2026.2654224)
Supplement: Supplementary materials — KGMR_A_2654224_SM1751.docx [file KGMR_A_2654224_SM1751.docx]

Supplementary Table 1: Study Characteristics of Included Articles

| **Study ID** | **Sample size** | **Age range/ Age mean ± SD**  **(years)** | **Sex (female)** |
| --- | --- | --- | --- |
| Aizawa et al^66^ | 100; MDD = 43 (30 on medication); HC = 57 | MDD = 39.4 ± 10.0; HC = 42.8 ± 12.7 | 53 (53%) MDD = 18 (41 (%); HC = 35 (61.4%) |
| Bai et al^67^ | 112; MDD = 56; HC = 56 | HC = 35.71 ± 15.99; MDD = 35.11 ± 16.79 | 74 (66.1%);  HC = 36 (64.3%); MDD = 38 (67.9%) |
| Bajaj et al^68^ | 93, PTSD = 29, non-PTSD = 64 | PTSD = 59.9 ± 8.4; None-PTSD = 58.8 ± 8.2 | 0 (0%) |
| Bosch et al^69^ | 3211; Dutch (*n* = 769), African Surinamese (*n* = 767), South-Asian Surinamese (*n* = 527), Turkish (*n* = 349), Moroccan (*n* = 473), and Ghanaian (*n* = 458) | 18–70; 50.4 ± 11.1 | 1773 (53%) |
| Busch et al^70^ | 78; MDD (Atypical) = 15; MDD (Typical) = 44; HC = 19 | MDD (Atypical) = 33.9 ± 12.3; MDD (Typical) = 28.9 ± 7.9; HC = 33.1 ± 7.2 | MDD (Atypical) = 12 (80 %); MDD (Typical) = 31 (70.4 %); HC = 11 (57.9 %) |
| Caso et al^71^ | 113 MDD = 68 (a-MDD = 46; r-MDD = 22).   HC = 45 | 43.98; a-MDD = 42.1;  r-MDD = 45.85; HC = 44.72 | 80 (77.77%) a-MDD = 36 (78.26%) r-MDD = 17 (77.27%) HC = 34 (75.5%) |
| Chen et al^50^ | 20; MDD = 10; HC = 10 | MDD = 43.9 ± 13.8; HC = 39.6 ± 9 | MDD = 5 (50%); HC = 5 (50%) |
| Chen et al^72^ | 48; MDD = 24; HC = 24 | MDD (female) = 41.5 ± 11.53; MDD (male) = 40.35 ± 11.05; HC (female) = 43.95 ± 12.11; HC (male) = 42.80 ± 15.13 | MDD = 24 (54.55%); HC = 24 (54.55%) |
| Chen et al^73^ | 60;  GAD = 36; HC = 24 | GAD = 46.08 ± 12.09; HC = 41.83 ± 13.93 | GAD = 20 (55.6%); HC = 14 (58.3%) |
| Chen et al^74^ | 141; MDD = 70 (young MDD = 25, middle-aged = 45); HC = 71 (young HC = 27, middle-aged HC = 44) | Young MDD = 24.0 ± 3.74, middle-aged MDD = 44.96 ± 7.76; Young HC = 24.96 ± 2.31, middle-aged HC = 47.16 ± 8.07 | MDD = 49 (70%) (young MDD = 18 (72%), middle-aged = 31 (68.9%)); HC = 53 (74.7%) (young HC = 19 (70.4), middle-aged HC = 34 (77.3%)) |
| Chen et al^75^ | 108; MDD = 62; HC = 46 | MDD = 39.58 ± 12.66; HC = 36.93 ± 8.58 | 100% |
| Chen et al^51^ | 20; MDD = 10; HC = 10 | MDD = 40.90 ± 14.86; HC = 38.20 ± 15.24 | MDD = 8 (80%); HC = 7 (70%) |
| Fatt et al^52^ | 179 | 46.2 ± 15.9 | 124 (69%) |
| Chung et al^53^ | 73;  MDD= 36; HC = 37 | MDD = 45.83 ± 14.08; HC = 41.19 ± 12.73 | MDD = 28 females (82.35%); HC = 23 females (62.16%) |
| Dong et al^54^ | 54; MDD = 23; GAD = 21; HC =10 | MDD = 30.04 ± 5.90; GAD = 30.43 ± 7.95; HC = 30.22 ± 6.50 | MDD = 16 (69.57%); GAD = 14 (66.67%); HC = 6 (60.00%) |
| Dong et al^55^ | 87; MDD= 57; HC = 30 | MDD = 29.01 ± 8.39; HC = 29.23 ± 6.57 | MDD = 37 (64.91%); HC = 20 (66.67%) |
| Ganci et al^44^ | 4610 (1143 males and 3467 females) | 18 - 87; 43.09 ± 13.48 | 3467 (75.2%) |
| Gao et al^76^ | 175; FHWs = 71; SHWs = 104 | 18-50 | Not Specified |
| Gao et al^77^ | 92; MDD = 62 (R = 37, TR = 25); HC = 32 | MDD -R = 20.67 ± 6.35; MDD-TR = 24.40 ± 8.26; HCs = 23.34 ± 3.13 | MDD-R = 29 (78.38%); MDD -TR = 9 (36%); HCs = 15 (46.88%) |
| Gonzalez-Mercado et al^78^ | 40 | 58 | 17 (42%) |
| Guo et al^79^ | 92; MDD = 46 (MDA0 = 20, MDA1 = 26); HC = 46 | HC = 26.00 ± 5.30; MDA0 = 26.85 ± 4.70; MDA1 = 27.96 ± 5.04 | HC = 32 (69.57%); MDA0 = 15 (75%); MDA1 = 17 (65.38%) |
| Hemmings et al^56^ | 30;  PTSD = 18; TE = 12 | PTSD = 42.0 ± 12.6; TE = 38.7 ± 11.7 | PTSD = 14 (77.8%); TE = 7 (58.3%) |
| Hope et al.^47^ | 20 | Not Specified | 15 (75%) |
| Hu et al^80^ | 293;  HC = 155; MDD = 138 (Mild = 24, Moderate = 72, Severe = 42) | 29.99 ± 7.31 | Not Specified |
| Huang et al^81^ | 61 | 31 ± 5; 20–42 | 32 females (52.45%) |
| Jiang et al^82^ | 76;  GAD= 40; HC = 36 | GAD = 33.4 ± 3.58; HC = 35.6 ± 6.98 | GAD = 30 (75%); HC = 23 (64%) |
| Jiang et al ^28^ | 76;   MDD = 46; HC = 30 | HC = 26.8 ± 5.4; A-MDD = 25.3 ± 5.4; R-MDD) = 27.1 ± 5.4 | HC: 50% (15/30); A-MDD = 38% (11/29); R-MDD = 47% (8/17) |
| Kim et al^83^ | 1238;  HC = 1071 (86.5%); MDD = 167 (13.5%) | HC = 45.69 ± 8.94; MDD = 44.0 ± 8.76 | HC = 356 female (33.24%); MDD = 92 female (55.09%) |
| Kim et al^84^ | 605 Anxiety = 337; HC = 267 | Anxiety group: Male = 45.65 ± 9.30, Female = 44.43 ± 9.13; HC: Male = 46.63 ± 8.78; Female = 43.52 ± 7.93 | 212 (35.0%); Anxiety group = 156 (46.3%); HC = 56 (20.9%) |
| Knudsen et al^85^ | 59; MDD = 27; HC = 32 | 18-24; MDD = 20.9 ± 4.2; HC = 23.7 ± 10.7 | 41 (69.5%); MDD = 16 (59.3%); HC = 25 (78.1%) |
| Lai et al^86^ | 55;  HC = 29; MDD = 26 four subgroups: SSRIs (*n* = 12), SNRIs (*n* = 7), other drugs (*n* = 2, mirtazapine and trazodone), and drug-free (*n* = 5) | HC = 39.41 ±10.96 (28–51); MDD = 43.73 ± 11.46 (32–52) | HC = 18 (62%); MDD = 16 (61.5%) |
| Li et al^87^ | 62;  Depression = 40; HC = 22 | Depression = 37.9 ± 14.3; HC = 44.0 ± 14.3 | Depression = 25 (62.5%); HC = 9 (40.9%) |
| Ling et al^88^ | 140;  HC = 48; MDD = 92 | 6-12; MDD = 8.84 ± 1.89; HC = 9.27 ± 2.11 | MDD = 50 (54.3%); HC = 26 (54.2%) |
| Liu et al^29^ | 100;  Control = 20; IBS-D = 40; Depression = 15; COMO = 25 | Control = 43.9 ± 11.2; IBS-D = 38.5 ± 13.6; Depression = 44.8 ± 14.9; COMO = 39.0 ± 13.9 | 47 (47%); Control = 13 (65%); IBS-D = 12 (30%); Depression: 11 (73.33%); COMO: 11 (44%) |
| Liu et al^89^ | 90;  MDD = 43; HC = 47 | MDD = 22.7 ± 1.8; HC = 21.7 ± 2.1 | MDD = 38 (88.4%); HC = 34 (72.3%) |
| Maes et al^90^ | 69; MDD = 32; HC = 37 | 19-58; MDD = 25.9 ± 9.1; HC = 28.4 ± 6.9 | MDD = 26 (81.3%); HC = 31 (83.8%) |
| Maes et al^91^ | 69; MDD = 32; HC = 37 | 19-58; MDD = 25.9 ± 9.1; HC = 28.4 ± 6.9 | MDD = 26 (81.3%); HC = 31 (83.8%) |
| Malan-Muller et al^57^ | 137;  PTSD = 79; TE = 58 | 44 (18-65); PTSD = 42; TE = 51 | 110 (80.29%); PTSD = 63 (79.74%); TE = 47 (81.03%) |
| Malan-Muller et al^58^ | 198; HC = 106; Case = 92 | 37; HC = 38 | 139 (70%); HC = 71 (67%) |
| Mason et al^59^ | 70;  HC = 10; MDD and Anxiety = 38; Anxiety = 8; MDD = 14 | HC = 33± 8.4; MDD and Anxiety = 39.2±10.6; Anxiety = 40.0 ± 13.7; MDD = 41.9 ± 12.0 | HC = 6 (60%); MDD and Anxiety = 31 (82%); Anxiety = 8 (100%); MDD = 11 (79% female) |
| Naseribafrouei et al^92^ | 55; MDD = 37; HC =18 | HC = 46.1 SD 13.9; MDD = 49.2 SD 13.9 | Not Specified |
| Ritchie et al^60^ | 117;  MDE and anxious distress = 63; MDE without anxious distress = 54 | MDE and anxious distress = 38.97 ± 14.21; MDE without anxious distress = 37.01 ± 12.22 | MDE and anxious distress = 41 (65.1 %); MDE without anxious distress = 38 (70.4 %) |
| Rong et al^93^ | 91;  MDD = 31; BP = 30; HC = 30 | MDD = 41.58 ± 10.40; BP = 38.40 ± 8.33; HC = 39.47 ± 10.22 | MDD = 22 (71.0%); BP = 15 (50%); HC = 16 (53.3%) |
| Tsai et al^43^ | 53;  LLD = 36; HC = 17 | 65.2 ± 7.7; LLD = 65.6 ± 7.3; HC = 64.1 ± 7.9 | 37 (69.8%); LLD = 28 (77.8%); HC = 9 (52.9%) |
| Wang et al^61^ | 50; Anxiety group = 21; HC = 29 | Anxiety group = 20.38 ± 1.12; HC = 20.97 ± 1.72 | 12 (24%); Anxiety group = (23.8%); HC = 7 (24.1%) |
| Yang et al^94^ | 311;  HC = 155; MDD = 156 | 18-65 | Not Specified |
| Ye et al^95^ | 54;  MDD = 26; HC = 28 | 26.04 ± 7.83 | HC= 21 (75%); MDD = 21 (81%) |
| Yu et al^96^ | 54;  MDD = 26; HC = 28 | 26.04 ± 7.83 | HC = 21 (75%); MDD = 21 (81%) |
| Yirmiya et al^97^ | 232 mother-child dyads initially recruited TE = 14; HC = 84 | Early childhood = 2.76 ± 0.91; Middle childhood = 7.68 ± 0.7; Late childhood = 9.3 ± 1.41; Early adolescence = 11.66 ± 1.23; Late adolescence = 16.13 ± 1.22 | 52.40% |
| Yuan et al^62^ | 240; UC (phase I) = 69; UC (phase II) = 60; MDD = 49; HC = 62 | UC (phase I) = 37 ± 53.6; UC (phase II) = 28 ± 46.7; MDD = 29 ± 59.2; HC = 36 ± 58.1 | UC (phase I) = 40 years; UC (phase II) = 40 years; MDD = 35 years; HC = 42 years |
| Zeamer et al^49^ | 51 | 52 | 26 (51%) |
| Zhang et al^63^ | 69; Placebo group = 31; LcS group = 38 | Placebo group = 49.7 ± 9.6; LcS group = 45.8 ± 12.3 | Placebo group = 20 (64.5%); LcS group = 24 (63.2%) |
| Zhang et al^45^ | 81; MDD = 36; HC = 45 | MDD= 36.81 ± 13.52; HC = 39.29 ± 11.44 | MDD = 15 (41.67%); HC = 26 (57.78%) |
| Zhang et al^46^ | 81; MDD = 36; HC = 45 | MDD = 36.81± 13.52; HC = 39.29 ± 11.44 | MDD = 15 (41.67%); HC = 26 (57.78%) |
| Zhang et al^64^ | 39 unmedicated MDD patients | 44.92 ± 2.53 | 29 (74.4%) |
| Zhao et al^98^ | 50; MDD = 24; Control = 26 | MDD = 29.96 ± 8.54; Control = 31.31 ± 9.70 | Not specified |
| Zheng et al^99^ | 60; MDD = 30; HC = 30 | PG = 30.80 ± 10.85; HG= 33.37 ± 7.02 | PG = 18 (60%); HG = 17 (56.7%) |
| Zhong et al^100^ | 261;  HC = 131; MDD = 130 | HC = 37.07 ± 14.22; Moderate MDD = 35.77 ± 13.92; Severe MDD = 37.88 ± 15.5 | HC = 89 (67.9%); Moderate MDD = 53 (67.95%); Severe MDD = 35 (67.31%) |
| Zhou et al^101^ | 57  PPD = 39; HC = 18 | HC = 32.57 ± 3.98; PPD = 33.64 ± 4.27 | 100% |
| Zhou et al^65^ | 171; PD = 101; Control = 70 | PD = 13.7 ± 2.6; Control = 13.5 ± 3.1 | PD = 56 (62.2%); Control = 54 (53.4%) |
| Zhou et al^102^ | Baseline = 179; PPD = 88; Completed the 8-week PPD = 79 | Acupuncture therapy = 35.35 ± 4.01; Control = 32.21 ± 3.16 | 100% |
| Zhu et al.,^48^ | 69; GAD = 23; Control = 46 | GAD = 55.30 ± 8.20; Control = 55.63 ± 7.78 | 35 (50.7)  GAD = 10 (43.5) Control = 25 (54.3) |
| Zhu et al^103^ | 90;  Anxiety/depression = 60; HC = 30 | HB = 22.25 ± 0.11; P = 22.5 ± 0.25; PB = 22.3 ± 0.25 | 50% |
| Zu et al^104^ | 114; MDD = 57; HC =57 | Not Specified | Not Specified |
| A-MDD = Active Major Depressive Disorder; FHWs = Frontline Health Care Workers; GAD = Generalized Anxiety Disorder; HB = Healthy Group; HC = Healthy Control HG = Healthy Control Group; LLD = Late-Life Depression; MDD = Major Depressive Disorder; MDDA1 = MDD with Anorexia; MDDA0 = MDD without Anorexia; MDE = Major Depressive Episode; OHE = Overt Hepatic Encephalopathy; P = Placebo Group; PB = Probiotic Group; PD = Pediatric Depression; PG = Patient Group; PPD = Post-Partum Depression; PTSD = Post-Traumatic Stress Disorder; R = Responders; R-MDD = Responding-MDD; SHWs = Second-line Health Care WorkersTE = Trauma-Exposed; TR = Treatment Resistant; UC = Ulcerative Colitis. | | | |

Supplementary Table 2: Methodological Characteristics of Articles Reviewed

| **Study Id** | **Sample type** | **Sample collection** | **DNA extraction** | **Microbiome Analytical Approaches** |
| --- | --- | --- | --- | --- |
| Aizawa et al^66^ | Fecal | Stool in RNAlater (2 mL)  Ambion RNAlater (Thermo Fisher Scientific, Waltham, MA)  Transport time and freezing temperature not specified | Not specified | Analytical method: 16S rRNA gene-targeted RT-qPCR (Yakult Intestinal Flora-SCAN®); |
| Bai et al^67^ | Fecal sample | Not specified | E.Z.N.A.® soil DNA Kit (Omega Bio-tek, Norcross, GA, U.S.). | Analytical method: 16S rRNA gene (V3-V4); Primers: not specified; Sequencing platform: Illumina MiSeq; Sequence reads: not specified; Demultiplexing: max barcode mismatches = 0; max primer mismatches = 2;  Adapter Trimming not specified; Quality filtering: Trimmomatic (Q < 20 over 50 bp, no ambiguous bases); Read merging: FLASH v1.2.7 (≥ 10 bp overlap);  Chimera removal: UCHIME; Clustering: UPARSE (USEARCH v7.1, 97% similarity);  Taxonomic assignment: RDP Classifier;  Alpha diversity: Chao1, Shannon;  Beta diversity: OPLS-DA;  Relative abundance: not specified;  Differential abundance: LEfSe. |
| Bajaj et al^68^ | Fecal sample | Not specified | Not specified | Analytical method: 16S rRNA gene;  Primers: not specified; Sequencing platform: IonTorrent PGM; Data processing and taxonomic assignment: not specified;  Alpha diversity: Shannon;  Relative abundance: not specified;  Differential abundance: LEfSe; Functional analysis: PiCRUST. |
| Bosch et al^68^ | Fecal sample | Fresh/frozen; home freeze if > 6 h delay; −20 °C storage ≤ 4 weeks; then −80 °C; local −20 °C storage | Not specified | Analytical method: 16S rRNA gene (V4); Primers: 515F and 806R; Sequencing platform: Illumina MiSeq V2; Sequence reads: paired-end 2x 250bp;  Demultiplexing: not specified;  Adapter Trimming: not specified; Quality control: FastQC;  Quality filtering: USEARCH v11.0.667 (max 1 expected error per merged contig); Read merging: USEARCH v11.0.667 (30 max accepted differences, 80% min overlap identity); Dereplication: UNOISE3 (α-parameter = 2.0; min 8 sequence occurrences in the entire dataset); ASV table construction: back-alignment of all merged reads to the inferred ASVs; Taxonomic assignment: SINTAX algorithm trained on Greengenes v13.5 and SILVA 132; Phylogenetic analysis: IQ-TREE v1.6.11 (MAFFT v.7.427, R-phytools);  Rarefaction: ASV table was rarefied to 14,942 counts per sample;  Alpha diversity: Shannon, Chao1, Observed ASVs, ACE, Simpson, Phylogenetic Diversity;  Beta diversity: Weighted UniFrac and Bray-Curtis distances (PCOA);  Relative abundance: rarefied ASV table;  FDR correction using Benjamini-Hochberg procedure; FDR-adjusted *p* < 0.05; |
| Busch et al^70^ | Fecal sample | Fresh; DNA/RNA Shield (Zymo); frozen within 15–30 min; stored at −80 °C | Genomic DNA Mini Kit (Invitrogen, Thermo Fisher Scientific, Langenselbold, Germany) | Analytical metho d:16S rRNA gene (V3-V4); Primers: not specified; Sequencing platform: Illumina iSeq V2; Sequence reads: paired-end 2x 150bp; PhiX removal: minimap2;  Read processing: LotuS v2.24 and implemented programs;  Dereplication: LotuS v2.24 (filter 8:1, 4:2, 3:3); minimap2; Chimera removal: VSEARCH UCHIME (RDP gold and de novo); Clustering: CD-HIT (97% similarity cutoff); Taxonomic assignment: RDP Classifier (-rdp_thr 0.8), QIIME2 BIOM file for abundance calculation; Phylogenetic tree: MAFFT and fasttree2;  Rarefaction: lowest sequencing depth; Alpha diversity : Shannon, Simpson, inverse Simpson;  Beta-diversity: Bray-Curtis distances visualized (NMDS);  Relative abundance: BIOM file generated from OTU tables;  Differential abundance: R-phyloseq; |
| Caso et al^71^ | Fecal sample | Fresh; icebox transport ≤1 h; stored at −80 °C | QIAamp DNA Stool Mini Kit (QIAGEN, Hilden, Germany); glass-bead beating steps on a Mini-beadbeater (FastPrep; Thermo Electron Corp.) | Analytical method: 16S rRNA gene amplicon sequencing, V3-V4 Primers: F (TCGTCGGCAGCGTCAGATGTGTATAAGAGACAGCCTACGGGNGGCWGCAG); R (TAGCAGAGACTTGGTCTGACTACHVGGGTATCTAATCC) Sequencing platform: Illumina MiSeq V3 Sequence reads: paired-end 2x 300bp  Demultiplexing: Not specified  Adapter Trimming: Not specified Quality filtering: prinseq-lite Read merging: fastq-join in ea-tools suite  Dereplication: Not specified  Chimera removal: Not specified Clustering: OTUs were clustered (no further details specified) Taxonomic assignment: RDP classifier (version and database not specified)  Alpha diversity : Shannon diversity; Rarefaction depth: 27,000 reads/sample; Statistical tests: Kruskal–Wallis, Wilcoxon rank-sum  Beta-diversity: Bray–Curtis, binary Jaccard; PCOA; PERMANOVA (999 permutations)  Relative abundance: Microbial composition at the genus level was quantified, and comparisons were made using Kruskal–Wallis and Wilcoxon rank sum tests |
| Chen et al^50^ | Fecal sample | Fresh, delivered within 15 min; stored at −80 °C | Proteins were extracted using SDS buffer, followed by boiling, sonication, trypsin digestion, and peptide quantification for LC-MS/MS analysis | Analytical method: Metaproteomics  Peptide and protein identification  Relative abundance: Normalized iTRAQ-labeled bacterial peptide intensities summed by COG categories.  Differential abundance: Identified using ≥1.5-fold change, permutation tests, and Mann–Whitney tests (*p* < 0.05)  Biomarker analysis: The study identified differentially expressed proteins. A total of 279 significantly differentially expressed bacterial proteins were detected, with statistical significance set at *p* < 0.05 |
| Chen et al^72^ | Fecal sample | Stored at −80 °C; no further details provided | PowerSoil® DNA Isolation Kit (MO BIO Laboratories, Carlsbad, CA, USA) | Analytical method: 16S rRNA gene amplicon sequencing, V3-V5 Primers: Not specified Sequencing platform: 454 sequencing system (Hoffman-La Roche, Switzerland) Sequence reads: Not specified Demultiplexing: Not specified  Adapter trimming: Not specified  Quality filtering: Mothur 1.31.2; Reads < 200 bp or > 1,000 bp, Barcode/primer mismatches, ambiguous bases, or had homopolymer runs > six bases  Read merging: Not specified  Dereplication: Not specified  Chimera removal: Not specified Clustering: Not specified Taxonomic assignment: RDP reference database  Alpha diversity: observed species, phylogenetic diversity, Shannon index, and Simpson index Beta diversity: PCOA, Further analysis - PLS-DA  Relative abundance: Differential abundance: Random forest analysis, Cytoscape 3.2.1, and LEfSe |
| Chen et al^73^ | Fecal sample | Fresh sterile cup; frozen at −80 °C within 15 min | QIAamp DNA Stool Mini Kit (Qiagen, Valencia, CA, USA) | Analytical method: 16S rRNA gene amplicon sequencing, V3-V4 Primers: 338F (5′- ACTCCTACGGGAGGCAGCAG-3′); 806R (5′-GGACTACHVGGGTWTCTAAT-3′) Sequencing platform: Illumina MiSeq Sequence reads: paired-end 2x 250bp Demultiplexing; Quality filtering: USEARCH v8.0 (zero index-mismatches, min overlap 50 bp, min final seq length 400 bp, error rate < 0.1)  Adapter Trimming: Not specified  Quality filtering: USEARCH v8.0; Overlap <50 bp, Length after merging <400 bp, Overlap error rate >0.1  Read merging: Not specified  Dereplication: Not specified  Chimera removal: UCHIME Clustering: UPARSE v7.1 (97% similarity cut-off) in the online platform from Sinotech Genome Technology Co. Chimera removal: UCHIME in the online platform from Sinotech Genome Technology Co. Taxonomic assignment: RDP Classifier (confidence threshold = 70%); SILVA database vSSU123  Alpha diversity metrics: Shannon–Wiener, Simpson index Beta diversity metrics: UniFrac (unweighted and weighted) distance visualized with PCoA. PERMANOVA (10,000 permutations)  Relative abundance: Differential abundance: Microbial community compositions (QIIME), LEfSe (α = 0.05; LDA effect size > 2)  SPSS v19.0 and R for statistical testing (chi-squared, *t*-test, Mann–Whitney *U*) |
| Chen et al^74^ | Fecal sample | Not Specified | PowerSoil® DNA Isolation Kit (MO BIO Laboratories, Carlsbad, CA, USA) | Analytical Method: 16S rRNA gene amplicon sequencing, V3-V5.  Primers: bar-coded universal primers containing linker sequences for pyrosequencing Sequencing platform: Roche 454  Sequence read: Not specified  Demultiplexing; Not specified  Adapter Trimming: Not specified Quality filtering: Mothur v1.31.2 (min seq length 200bp, max seq length 1000bp; no homopolymers > 6 bases)  Read merging: Not specified  Dereplication: Not specified  Chimera removal: Not specified Clustering: Mothur v1.31.2 (97% similarity threshold) Taxonomic assignment: RDP database  Alpha diversity: Chao1, ACE  Beta diversity: Not specified; however, OPLS-DA was used to identify group differences  Relative abundance: Microbial abundances were analysed at the family and genus levels.  Differential abundance: LEfSe (LDA scores > 2), Random forest analysis, ROC curve analysis, AUC |
| Chen et al^75^ | Fecal sample | Fresh sterile cup; frozen at −80 °C within 30 min. | Qiagen QIAamp DNA Stool Mini Kit (Qiagen) | Analytical method: 16S rRNA gene amplicon sequencing, V3-V4  Primers: V3-V4: 341F (5′-CCTACGGGNGGCWGCAG-3'); 805R (5'-GACTACHVGGGTATCTAATCC-3')  Sequencing platform: Illumina MiSeq  Sequence reads: not specified Demultiplexing; Quality-filtering: USEARCH v8.0 Clustering: UPARSE v7.1 (97% sequence identity cutoff) Taxonomic assignment: RDP Classifier  Rarefaction: normalized OTU abundance table was used for beta-diversity analysis Alpha diversity: Chao1, ACE, Shannon, Simpson Beta diversity: UniFrac (unweighted and weighted) distances, visualized/separated with PCoA Relative abundance: Differential abundance: OTUs, Wilcoxon test (*p* < 0.05), Random forest analysis, ROC curve analysis, AUC  Analytical method: Shotgun whole genome analysis Sequencing platform: Illumina NovaSeq 6000 Sequence reads: paired-end 2x 150bp reads Sequence filtering: remove bases with mass value less than 20 and sequences less than 30bp Assembly; Short read splicing: Megahit v1.1.3 ORF prediction: MetaGeneMark v3.38 Gene clustering: CD-HIT v4.6.8. Gene annotation: Bowtie2 v2.3.4.1 trained on non-redundant gene sets. Gene abundance estimation: eXpress v1.5.1.  Taxonomic assignment (profiling): Metaphlan2 v2.0. Functional analysis: DIAMOND (v0.0.19) to compare gene set protein sequence with the KEGG database to obtain KO numbers. Bowtie2 v2.3.4.1 trained on non-redundant gene sets. Gene abundance estimation: eXpress v1.5.1 Alpha diversity: Shannon index  Beta diversity : Unifrac (Weighted and Unweighted); PCoA  Relative abundance: Differential abundance: Mann-Whitney *U* and Wilcoxon tests with FDR correction. LEfSe identified differentially abundant taxa. |
| Chen et al^51^ | Fecal sample | Refrigerated at home; delivered at 4 °C next day; stored at −80 °C | QIAamp DNA Stool Mini Kit (QIAGEN Inc., USA) and custom protocol for phenol–chloroform extraction method | Analytical Method: 16S rRNA gene amplicon sequencing, V4 and V3-V4 regions Primers: V4: 515F (5′-TGCCAGCMGCCGCGGTAA-3'); 806R (5'-GGACTACNNGGGTATCTAAT-3'); V3-V4: 5'-CCTACGGGNGGCWGCAG-3'; 3'-GACTACHVGGGTATCTAATCC-5')  Sequencing platform and sequence reads: V4: Illumina MiniSeq; V3-V4: Illumina Miseq. Sequence reads: V4: paired-end 2x 250 bp; V3-V4: paired-end 2x 300 bp  Demultiplexing: Not specified  Adapter Trimming: fastx_clipper Quality filtering: fastq_quality_filter in FASTX (-q 20 -p 70) Read merging: pandaseq; note: after read merging the V3 region was removed from the V3-V4 reads using fastx_clipper in FASTX  Dereplication: Not specified  Chimera removal: Not specified Clustering: closed-reference picking script in QIIME v1.7.0 Taxonomic assignment: QIIME v1.7.0 trained on the Greengenes database v13.5 (97% similarity cut-off) Alpha diversity: Richness, Shannon Beta diversity : Bray-Curtis distances; PCoA, Wilcoxon rank sum test  Relative abundance: Differential abundance: Differential miRNA expression between MDD and controls was analysed with log2-transformed, quantile-normalized data and visualized by a volcano plot. Multiple testing corrections used FDR < 0.05 |
| Fatt et al^52^ | Fecal sample | Chilled/frozen at home; returned within 48 h; frozen at −80 °C; collected within 1 week of clinical assessments. | Mechanical and enzymatic lysis; followed by phenol:chloroform extraction and a clean-up step. | Analytical method: 16S rRNA gene amplicon sequencing, V3-V4 Primers: 341F (CCTACGGGNGGCWGCAG); 802R (GGACTACNVGGGTWTCTAAT)  Sequencing platform and sequence reads: not specified Sequencing data processing: DADA2  Demultiplexing; Not specified  Adapter Trimming: Not specified  Quality filtering: Performed as part of DADA2 pipeline. Samples were also filtered for sequencing depth and α-diversity outliers (Shannon index < threshold)  Read merging: DADA2  Dereplication: Not specified  Chimera removal: Not specified Clustering: ASV-based analysis (Retained ASVs: prevalence > 50% or mean relative abundance > 0.1% and prevalence > 10%; remaining ASVs aggregated to genus level using tax_glom in phyloseq) Taxonomic assignment: ASV annotation trained on the SILVA database v128 (software not specified)  Alpha diversity: Shannon index Beta diversity: Not specified Relative abundance: CLR-transformed taxa abundances (WGCNA)  Differential abundance: ALDEx2 (p < 0.05) and random forest (top 20 important taxa). Taxa significance in WGCNA (correlation with traits) |
| Chung et al^53^ | Fecal sample | Collected within 2 weeks of interview and assessments; delivered at 4 °C; stored at −80 °C; transport time not specified | QIAamp DNA Stool Mini Kit (QIAGEN Inc., USA) and custom protocol for phenol–chloroform extraction method | Analytical method: 16S rRNA gene amplicon sequencing, V3-V4 and V4 Primers: V3-V4: 5′-TCGTCGGCAGCGTCAGATGTGTATAAGAGACAGCCTACGGGNGGCWGCAG-3'; 5′-GTCTCGTGGGCTCGGAGATGTGTATAAGAGACAGGACTACHVGGGTATCTAATCC-3′  Primers: V4: 5′-TCGTCGGCAGCGTCAGATGTGT ATAAGAGACAGGTGCCAGCMGCCGCGGTAA-3'; 5′GTCTCGTGGGCTCGGAGATGTGTATAAGAGACAGGGACTACHVGGGTWTCTAAT-3′. " Sequencing platform: Illumina MiSeq (V3-V4) and MiniSeq (V4) Sequence reads: Not specified  Demultiplexing: Not specified  Adapter Trimming: Not specified Quality filtering: QIIME v1.9.1 (default parameters)  Read merging: PEAR v0.9.8 (min overlap 5 bp)  Dereplication: QIIME/DADA2  Chimera removal: DADA2 Clustering: QIIME v1.9.1  Taxonomic assignment: QIIME v1.9.1 trained on the Greengenes database vMay13 (97% similarity cut-off)  Alpha diversity: Shannon, Chao1, Observed OTUs, PD Whole Tree  Beta diversity: UniFrac (unweighted and weighted) distance; PERMANOVA  Relative abundance: Summarized from 129 taxa (with > 0.01% abundance) across phylum to genus levels  Differential abundance: ANCOM (α = 0.05) |
| Dong et al^54^ | Fecal sample | Fresh; frozen immediately in sterile cups at collection; stored at −80 °C before analysis | QIAamp DNA Stool Mini Kit (Qiagen, Hilden, Germany) | Analytical method: 16S rRNA gene amplicon sequencing, V3-V4 Primers: not specified, but following NIH Human Microbiome Project protocols (16S 454 Sequencing Protocol HMP consortium; https://www.hmpdacc.org) Sequencing platform: Illumina MiSeq  Sequence reads: Not specified  Demultiplexing; Not specified  Adapter Trimming: Not specified Quality filtering: QIIME2 pipeline  Read merging: UPARSE and QIIME2 protocol  Dereplication: UPARSE  Chimera removal: UCHIME (v4.1) Clustering: UPARSE (97% similarity)  Taxonomic assignment: RDP Classifier trained on the SILVA database vSSU138 (confidence threshold 70%)  Rarefaction: normalization of the sequence number of each sample was performed (no further specifications provided). Alpha diversity: ACE, Chao1, Simpson, Shannon indices Beta diversity: Bray-Curtis dissimilarity Relative abundance:  Differential abundance: Assigning taxonomy to clustered OTUs, with microbial relative abundances compared between groups using ANOVA (*p* < 0.05) and corrected for false discovery (Benjamini–Hochberg) OTUs. Other analysis –PiCRUST, Cytoscape, KEGG ortholog correlation |
| Dong et al^55^ | Fecal sample | Fresh; frozen immediately in sterile cups at collection; stored at −80 °C before analysis | QIAamp DNA Stool Mini Kit (Qiagen, Hilden, Germany) | Analytical method: 16S rRNA gene amplicon sequencing, V3-V4  Primers: 338F (5′-ACTCCTACGGGAGGCAGCAG-3'); 806R (5'-GGACTACHVGGGTWTCTAAT-3').  Sequencing platform: Illumina MiSeq Sequence reads: Not specified  Demultiplexing; Not specified  Adapter Trimming: Not specified Quality filtering: QIIME2 pipeline  Read merging: Not specified  Dereplication: UPARSE  Chimera removal: UCHIME v4.1 OTU identification: OTU analysis: QIIME2 Denoising: QIIME2 and UNOISE3  Clustering: UPARSE v7.1 (97% similarity) after normalization  Taxonomic assignment: RDP Classifier trained on the SILVA database vSSU138 (confidence threshold 70%)  Rarefaction: A rarefaction curve was used to validate the amount of sequencing data and the richness of species in the samples  Alpha diversity: Chao1, Shannon indices Beta-diversity: Bray–Curtis dissimilarity visualized with NMDS using the vegan (v 2.5-7) package in R (v R-4.0.5); Other diversity analysis: ANOSIM tests to identify differences in β-diversity between the two groups  Relative abundance:  Differential abundance: STAMP and Welch’s *t*-test method (*p* value < 0.05) were used to identify key species and strain classification responsible or the differences between the MDD and HC groups  Analytical method: Shotgun whole genome sequencing  Sequencing platform: Illumina NovaSeq Assembly (single strains): MetaWRAP (completeness > 75; contamination < 5)  Clustering: MetaPhlAn3 for profiling; MetaWRAP used to assemble individual strains (filtered for completeness > 75% and contamination < 5%) Taxonomic assignment: MetaPhlAn3 |
| Ganci et al^44^ | Fecal sample | Collected in FMA kit with anaerobic pouch; transported in polystyrene boxes with ice packs below 12 °C; delivered within 48 hours | Microbial culturing; quantification; and morphological examination of colonies were performed instead of DNA extraction | Analytical method: Fecal microbial analysis |
| Gao et al^76^ | Fecal sample | Fresh fecal samples collected in MGIEasy Stool DNA preservation tubes; stored at −80 °C. | E.Z.N.A® DNA Kit (Omega Biotek, Norcross, GA, U.S.) | Analytical method: 16S rRNA gene amplicon sequencing, full length (V1-V9) Sequencing platform: PacBio Sequel  Sequence reads: Circular consensus sequence  CCS read generation: SMRT Link v9.0 (min number of passes = 3, min. predicted accuracy = 0.99) Length filtering; Quality filtering: SMRT Portal (length < 800 bp or > 2500 bp) Denoising; Deblur denoising algorithm (100% similarity)  Clustering: ASVs at 100% similarity  Taxonomic assignment: UCLUST algorithm in USEARCH v11 using the SILVA database vSSU132 (98.6 sequence identity threshold)  Alpha diversity: Chao1, Faith PD, Goods coverage, Shannon, Simpson, Pielou Observed species indices Beta diversity: Bray-Curtis dissimilarity visualized with PCoA, PERMANOVA  Relative abundance: Differential abundance: Random Forest |
| Gao et al^77^ | Fecal sample | Fresh, mid-stool; clean toilet; sealed, labeled, and stored immediately at −80°C; freeze-thaw cycles avoided. | QIAamp DNA Stool Mini Kit (Qiagen, Hilden, Germany) | Analytical Method: 16S rRNA gene amplicon sequencing, V3-V4. Primers: 338F-806R Sequencing platform: Illumina MiSeq Sequence reads: paired-end 2x 300 bp  Demultiplexing; Not specified  Adapter Trimming: Not specified Quality filtering: QIIME2 pipeline  Read merging: Not specified  Dereplication: Not specified  Chimera removal: Not specified  Clustering: QIIME Taxonomic assignment: OTU representative sequences were compared trained on the Greengenes database  Alpha diversity: Chao1, Faith PD, Goods coverage, Shannon, Simpson, Pielou’s evenness, Observed species indices Beta-diversity metrics: Jaccard dissimilarity visualized with PCoA  Relative abundance: Differential abundance: LEfSe (LDA scores > 3.5), Metastats, ROC |
| Gonzalez-Mercado et al^78^ | Fecal sample | Not specified | PowerSoil® DNA Isolation Kit (MO BIO Laboratories, Carlsbad, CA, USA) | Analytical method: 16S rRNA gene amplicon sequencing, V3-V4 Primers: Not specified Sequencing technology: Illumina (platform not specified) Sequence reads: Not specified  Demultiplexing; Not specified  Adapter Trimming: Trim Galore! v0.44 (Q = 25) Quality filtering: DADA2 plugin in QIIME2 v2019.17 Denoising: DADA2 plugin in QIIME2 v2019.17  Read merging: Not specified  Dereplication: Not specified  Chimera removal: Not specified  Clustering: Not performed; instead, ASV-level resolution used via DADA2  Taxonomic assignment: QIIME2 naive Bayes classifier trained on the SILVA database v132  Rarefaction: rarefaction of the ASV table to 4,226 sequences/sample Alpha diversity: Not performed/specified Beta diversity: Not performed/specified  Relative abundance: Differential abundance: Predicted from the rarified table (QIIME2; q2-picrust2 command). Other analysis - MetaCyc pathway |
| Guo et al^79^ | Fecal sample | Fresh; immediately frozen at −80 °C | QIAamp DNA Stool Mini Kit (QIAGEN, Hilden, Germany) | Analytical method: 16S rRNA gene amplicon sequencing, V3-V4 Primers: 338F (5'-ACTCCTACGGGAGGCAGCA-3'); 806R (5'-GGACTACHVGGGTWTCTAAT-3') Sequencing platform: Illumina NovaSeq PE250 platform Sequence reads: paired-end 2x 250bp  Demultiplexing; QIIME2 v2019.1  Adapter Trimming: Not specified Quality filtering: QIIME2 v2019.1  Chimera removal: QIIME 2 v2019.1 Denoising; Sample inference: QIIME2 v2019.1 Clustering: QIIME2 v2019.1 (ASVs, 100% similarity cut-off) Taxonomic assignment: no details provided  Alpha diversity: Chao1, Faith's PD, Goods coverage, Shannon, Simpson, Pielou’s evenness, Observed species indices. Beta-diversity metrics: Bray-Curtis dissimilarity visualized with PCoA  Relative abundance: Differential abundance: LEfSe |
| Hemmings et al^56^ | Fecal sample | Not specified | PSP® Spin Stool DNA Plus Kit (STRATEC Molecular, Birkenfeld, Germany) | Analytical Method: 16S rRNA gene amplicon sequencing, V3-V4 Primers: Klindworth et al., 2013: 341F (5'-CCTACGGGNGGCWGCAG-3'); 785R (5'-GACTACHVGGGTATCTAATCC-3') Sequencing platform: Illumina HiSeq Sequence reads: paired-end 2x 100 bp  Demultiplexing: QIIME2 v2019.1 (default parameters with min quality score = 25)  Adapter trimming: Illumina Casava Pipeline v1.8.2 (removing reads containing adaptors and/or PhiX control) Quality control: 1st assessment: data passing the Illumina Chastity filter; 2nd assessment: FASTQC v0.10.0  Read merging: Not specified  Dereplication: Not specified  Chimera removal: Not specified Clustering: closed-reference script in QIIME v1.9 Taxonomic assignment: QIIME v1.9 using the Greengenes database (97% similarity cut-off)  Rarefaction analysis: 30,000 seqs per sample/person and filtering to retain OTUs present in at least 20% of the participants Alpha diversity: Chao1, observed OTUs, PD whole tree and Shannon diversity indices Beta diversity: Bray-Curtis dissimilarity and UniFrac (unweighted and weighted) distances Relative abundance: OTUs were taxonomically collapsed and analysed for relative abundances across samples  Differential abundance: Random Forest analysis |
| Hope et al.^47^ | Fecal sample | Collected at home using a kit with three tubes (Fisher Scientific), placed in biohazard and padded freezer bags with ice packs, frozen immediately, and delivered or shipped within 24 hours; stored at −80 °C until DNA extraction | PowerSoil isolation kit (Mo Bio Laboratories, Carlsbad, CA, US) | Analytical method: 16S rRNA gene sequencing, V3-V4 Primers: 341F-805R (no sequences specified) Sequencing platform: Illumina MiSeq V3 Sequence reads: paired-end 2x 100 bp  Demultiplexing; Not specified  Adapter Trimming: Not specified Quality filtering: DADA2 in QIIME2 (2019) Denoising: DADA2 in QIIME2 (2019)  Read merging: Not specified  Dereplication: Not specified  Chimera removal: Not specified Clustering: ASVs were generated using DADA2 Taxonomic assignment: QIIME2 pre-trained classifier trained on the SILVA database data in QIIME to correct for sequencing depth  Alpha-diversity: Shannon, Chao1, Faith’s PD, Pielou’s evenness indices Beta-diversity: Jaccard and weighted UniFrac distances visualized with PCoA. PERMANOVA, Spearman correlations (continuous var) and Kruskal-Wallis pairwise test (categorical var)  Relative abundance: Taxonomic profiles assessed at phylum and genus levels  Differential abundance: LEfSe |
| Hu et al^80^ | Fecal sample | Fresh stool in sterile tubes (7–10 am), stored at 4 °C, transferred to −80 °C within 6 h | E.Z.N.A. Soil DNA Kit (Omega Bio-Tek, Norcross, GA, USA) | Analytical method: Shotgun whole genome sequencing  Sequencing platform: Illumina NovaSeq Sequence reads: paired-end (length not specified) Quality filtering: Sickle (min seq length 50 bp; no homopolymers > 10 bp; no ambiguous base calls)  Host contamination removal: BWA trained on the human genome Assembly: MEGAHIT (min seq length 300 bp) ORF prediction: Metagene Gene clustering: CD-HIT (95% seq id cutoff)  Taxonomic assignment (profiling): Metaphlan2 v2.0 Re-alignment: SOAPaligner Enterotype clustering: Dirichlet multinomial mixtures (DMM) for clustering; Calinksi-Harabasz index for defining optimal cluster numbers  Functional analysis: gene set annotations using Diamond v0.8.35 trained on the NCBI database, gene set alignments trained on the KEGG db (e-value cutoff 1 × 10^−5^).  Alpha diversity: Dominance, Simpson, Shannon and Evenness indices  Beta diversity: Bray-Curtis dissimilarity visualized with PCoA Other diversity: PERMANOVA to test the overall and pairwise beta diversity group differences Relative abundance: Species level was calculated based on Reads Per Kilobase Million  Differential abundance: LEfSe, (LDA scores > 2.5, *p* < 0.05), Random Forest, ROC curve |
| Huang et al^81^ | Fecal sample | Fresh stool in 5 mL sterile tubes (screwcap with spoon), collected ±1 week of MRI, stored at −80 °C immediately | QIAamp DNA Stool Mini Kit (QIAGEN, Hilden, Germany) | Analytical method: 16S rRNA gene amplicon sequencing, V3-V4 Primers: Not specified, amplicon size selection at 550 bp Sequencing platform: Illumina MiSeq V3 Sequence reads: paired-end 2x 300 bp  Demultiplexing; Illumina Standard Analysis Pipeline  Adapter Trimming: Not specified  Quality filtering: QIIME2  Read merging: Not specified  Dereplication: Not specified  Chimera removal: Not specified  Clustering: QIIME (97% similarity threshold) Taxonomic assignment: RDP Classifier using the Greengenes database (version not specified)  Rarefaction: even sampling depth 50,000 reads Alpha diversity: Chao1, Shannon, Simpson indices  Beta diversity: Not processed/specified Relative abundance: Differential abundance: Calculated using QIIME's Taxa summary on multiple taxonomic levels; Other analysis - FDR correction was conducted for multiple comparisons (*p* < 0.05) |
| Jiang et al^82^ | Fecal sample | Fresh; Self-collected in sterile plastic cups; delivered within 30 min; stored at −80 °C | QIAamp DNA Stool Mini Kit (Qiagen, Hilden, Germany); glass-bead beating step on a mini bead beater (FastPrep; Thermo Electron Corp.) | Analytical method: 16S rRNA gene amplicon sequencing, V3-V4 Primers: 338F (ACTCCTACGGGAGGCAGCAG); 806R (GGACTACHVGGGTWTCTAAT) Sequencing platform: Illumina MiSeq V3 Sequence reads: paired-end 2x 300 bp Demultiplexing/Quality-filtering: Trimmomatic and FLASH (versions not specified)  Read merging: FLASH Chimera removal: UCHIME (version not specified) Clustering: QIIME v1.7 (97% similarity threshold Taxonomic assignment: Not specified  Alpha diversity: Shannon, Chao1  Beta diversity: weighted UniFrac visualized with PCoA; PERMANOVA Relative abundance: Computed from OTUs (QIIME  Differential abundance: LEfSe (alpha = 0.05, LDA scores > 2) |
| Jiang et al ^28^ | Fecal sample | Fresh; collected in sterile plastic cup post-assessment; kept in icebox; delivered within 15 min; stored at −80 °C | QIAamp DNA Stool Mini Kit (Qiagen, Hilden, Germany); glass-bead beating step on a mini bead beater (FastPrep; Thermo Electron Corp.) | Analytical method: 16S rRNA gene amplicon sequencing, V1-V3 Primers: 27F (5'-AGAGTTTGATCCTGGCTCAG-3'); 533R (5'-TTACCGCGGCTGCTGGCAC-3') Sequencing platform: Roche 454 FLX system.  Demultiplexing; Mothur (v1.25.0) and custom Perl scripts  Adapter Trimming: Not specified  Quality-filtering: Mothur v1.25.0 and custom Perl scripts  Denoising: Titanium PyroNoise software  Read merging: Not specified  Dereplication: Not specified Chimera removal: ChimeraSlayer Clustering: Mothur v1.25.0 (97% threshold) Taxonomic assignment: RDP Naïve Bayesian Classifier (50% bootstrap score)  Rarefaction: rarefaction curve analyses with Mothur v1.25.0  Alpha diversity: Shannon, Simpson, ACE, Chao1, Good’s Coverage indices, Venn diagram,  Beta diversity metrics: unweighted UniFrac distance and PCoA Relative abundance: Differential abundance: Metastats (Mothur); LEfSe (alpha = 0.05, LDA scores > 2). Kruskal–Wallis test |
| Kim et al^83^ | Fecal sample | Fresh; collected at home in sterile screw-cap containers (no preservative), frozen at −20 °C, delivered within 24 h, stored at −70 °C | PowerSoil® DNA Isolation Kit (MO BIO Laboratories, Carlsbad, CA, USA) | Analytical method: 16S rRNA gene amplicon sequencing, V3-V4 Primers: 341F; 805R (sequences not specified) Sequencing platform: Illumina MiSeq Sequence reads: paired-end 2x 300 bp  Demultiplexing; Not specified  Adapter Trimming: DADA2 pipeline Quality filtering: DADA2 plugin in QIIME2 v2.0, 2020.8  Denoising: DADA2 plugin in QIIME2 v2.0, 2020.8)  Read merging: DADA2  Dereplication: DADA2 Chimera removal: DADA2 plugin in QIIME2 v2.0, 2020.8  Clustering: ASVs generated using DADA2 Taxonomic assignment: QIIME2 v2019.17 (q2-feature-classifier command) trained on the SILVA database v132 V4 (99% identity cut-off)  Rarefaction: 5011 reads per sample  Alpha diversity : Observed ASVs, Shannon indices, Pielou’s evenness, Faith's PD  Beta diversity: Bray-Curtis dissimilarity, Jaccard and UniFrac (unweighted and weighted) UniFrac distances  Relative abundance: Differential abundance: MaAsLin. Other analysis Mann–Whitney, Spearman rank correlation, PERMANOVA, STAMP v2.1.3, PICRUSt2, (MetaCyc database) |
| Kim et al^84^ | Fecal sample | Self-collected fecal swabs, frozen at −20 °C, stored at −70 °C for 24 h | PowerSoil® DNA Isolation Kit (MO BIO Laboratories, Carlsbad, CA, USA) | Analytical method: 16S rRNA gene amplicon sequencing, V3-V4 Primers: 341F (5′-TCGTCGGCAGCGTCAGATGTGTATAAGAGACAGCCTACGGGNGGCWGCAG-3′); 805R (5′-GTCTCGTGGGCTCGGAGATGTGTATAAGAGACAGGACTACHVGGGTATCTAATC C-3′) Sequencing platform: Illumina MiSeq Sequence reads: Not specified  Demultiplexing; Not specified  Adapter Trimming: DADA2 pipeline Quality-filtering: DADA2 plugin in QIIME2 v2.0, 2021.4  Denoising: DADA2 plugin in QIIME2  Read merging: DADA2  Dereplication: DADA2 Chimera removal: DADA2 plugin in QIIME2 v 2.0  Clustering: ASVs generated using DADA2  Taxonomic assignment: Naïve Bayes classifier in QIIME2 (q2-feature-classifier command) trained on the SILVA database v r138.1  Rarefaction: 5011 reads per sample  Alpha diversity: Observed ASVs, Shannon indices, Pielou’s evenness, Faith's PD  Beta diversity: Bray-Curtis dissimilarity, Jaccard and UniFrac (unweighted and weighted) UniFrac distances  Relative abundance: Differential abundance: MaAsLin; LEfSe; Other analysis - Mann–Whitney, Spearman rank correlation, PERMANOVA, Welch’s non-parametric *t*-test via STAMP v2.1.3, PICRUSt2, (MetaCyc database) Bonferroni (*p* < 0.05) |
| Knudsen et al^85^ | Fecal sample | Fresh; home-collected, stored at −20 °C (max 72 h), delivered in cooling bag, stored at −80 °C | QIAamp Powerfecal DNA kit (QIAGEN) | Analytical method: 16S rRNA gene amplicon sequencing, V4 Primers: (515 F(Parada) and 806R(Apprill)) Sequencing platform: Illumina MiSeq V3 Sequence reads: paired-end 2x 300 bp  PhiX removal; Demultiplexing: USEARCH v11  Adapter Trimming: QIIME2 Quality-filtering: QIIME2 v2020.8 (truncation to 250bp) Denoising: DADA2 plugin in QIIME2 v2020.8 Phylogenetic tree: MAFFT and fasttree2 in QIIME2 (q2-alignment and q2-phylogeny commands)  Read merging: DADA2  Dereplication: DADA2 Chimera removal: Not specified  Clustering: Not specified Taxonomic assignment: Naïve Bayesian classifier in QIIME2 (q2-feature-classifier command) trained on the SILVA database v138 SSU  Alpha diversity: ASV richness, Faith’s PD and Shannon indices  Beta diversity: Bray-Curtis dissimilarity and UniFrac (unweighted and weighted) visualized with PCoA Relative abundance Differential abundance: ANCOM-BC. Multiple testing was corrected using Benjamini-Hochberg FDR (*q* < 0.05); Other analysis – PERMANOVA, Student’s *t*-test /Mann-Whitney *U* test |
| Lai et al^86^ | Fecal sample | Fresh; collected the completion of questionnaire assessments; immediately frozen at −80 °C | StoolGen DNA kit (CWBiotech Co., China). | Analytical method: Shotgun whole genome sequencing Sequencing platform: Illumina HiSeq2500  Sequence analysis: MEGAN5  Filtering and Preprocessing: Prevalence < 80% in all samples Taxonomic profiling: MEGAN5  Alpha diversity: Fisher, Shannon Beta diversity: Bray-Curtis dissimilarity and visualized with PCoA.  Relative abundance: Differential abundance: LEfSe (alpha = 0.05, LDA scores ≥ 3.0), FDR-adjusted *p* < 0.05; Random Forest and Boruta machine learning algorithm (> 0.1% genus, > 0.01% species). Other analysis: Mann–Whitney *U*, PERMANOVA, the metagenomeSeq test (with zero-inflated Gaussian fit statistical model) |
| Li et al^87^ | Fecal sample | Fresh; collected within 24 hours of admission in a sterile plastic box; stored at − 80 °C | StoolGen DNA kit (Beijing Youji Technology Co., China). | Analytical method: 16S rRNA gene amplicon sequencing, V4 Primers: 338F (5′-ACTCCTACGGGAGGCAGCA-3′); 806R (5′-GGACTACHVGGGTWTCTAAT-3′) Sequencing platform: Illumina MiSeq V3 Sequence reads: paired-end 2x 250 bp  Demultiplexing: Not specified  Adapter Trimming: Not specified Quality-filtering: internal script filters out low-quality reads; if a 30-bp window started truncating read end seqs, the final read length <75% of the reads were removed Read merging: FLASH v1.2.11  Dereplication: Not specified Chimera removal: Not specified  Clustering: OTU-based clustering at 97% similarity Taxonomic assignment: RDP Classifier v2.2 trained on the Greengenes database v2013_5_99  Alpha diversity: Observed species, Chao, ACE, Shannon, Simpson  Beta diversity: PCA  Relative abundance: OTU relative abundances were calculated. Statistical tests (rank sum, Fisher’s exact, *t*-test, variance test) with Benjamini-Hochberg FDR correction identified taxa at multiple taxonomic levels (phylum to species) |
| Ling et al^88^ | Fecal sample | Fresh; 2 g of stool; sterile plastic cup; stored at −80 °C within 15 minutes until use | QIAamp DNA Stool Mini Kit (Qiagen, Hilden, Germany) | Analytical method: 16S rRNA gene amplicon sequencing, V3-V4 Primers: 341F (5’-CCTACGGGNGGCWGCAG-3’); 785R (5’-ACTACHVGGGTATCTAATCC-3’) Sequencing platform: Illumina MiSeq V3 Sequence reads: paired-end 2x 300 bp Sequence processing; Quality control: QIIME2 v2020.11 (default parameters) Demultiplexing: Performed (inferred as standard in Illumina MiSeq workflow)  Adapter Trimming: Not specified  Quality-filtering: Performed in QIIME2 (v2020.11) using default parameters  Read merging: Not specified  Dereplication: Not specified Chimera removal: Not specified  Clustering: OTU clustering at 97% similarity Taxonomic assignment: RDP Classifier and UCLUST v1.2.22 methods in QIIME2 trained on the Greengenes database v13.8.  Rarefaction: normalization to even sampling depths Alpha diversity: observed species, ACE, Chao1, Shannon, Simpson, Evenness and PD whole tree  Beta diversity: Jaccard and UniFrac (unweighted and weighted) distances and Bray-Curtis dissimilarity  Relative abundance: Differential abundance: STAMP software v.2.1.3 and LEfSe (selecting only phylotypes with an av. rel. abund. of > 0.01%). ROC and AUC analysis, PiCRUSt v1.0.0 |
| Liu et al^29^ | Fecal sample | Fresh; stored at −80 °C immediately after collection | PowerSoil® DNA Isolation Kit (MO BIO Laboratories, Carlsbad, CA, USA) | Analytical method: 16S rRNA gene amplicon sequencing, V1-V3 Primers: 27F (5’-AGAGTTTGATCCTGGCTCAG-3’); 533R (5’-TTACCGCGGCTGCTGGCAC-3’), and MID tags (for distinguishing the direction of the sequence: 5’-ATTACCGCGGCTGCTGGCA-3’) Sequencing platform: Roche 454 GS FLX+ Titanium platform  Demultiplexing: MID tags  Adapter Trimming: Not specified  Quality-filtering: Not specified Read alignment: align.seqs function in Mothur Distance matrix: dist.seqs function in Mothur Clustering: cluster function in Mothur Taxonomic assignment: blastall function in BLAST (e-value 10^-5^ or less and the highest score) trained on the RDP database  Rarefaction: Mothur (make.shared and rarefaction.shared functions) to transform OTU information into rarefaction data (sampling identity 100%, 97%, 95%) Alpha diversity: Shannon index  Beta diversity: PCA |
| Liu et al^89^ | Fecal sample | OMNIgene•GUT kit (DNA Genotek), stored at −80 °C upon receipt | ZymoBIOMICS™ DNA Kit (Zymo Research) | Analytical method: 16S rRNA gene amplicon sequencing, V4 Primers: 515F (5′-GTGYCAGCMGCCGCGGTAA-3'); 806R (5'-GGACTACNVGGGTWTCTAAT-3') following the Earth Microbiome Protocol Sequencing platform: Illumina MiSeq Sequence reads: paired-end 2x 250 bp Sequence import; Demultiplexing: tools and demux plugins in QIIME2 v2019.17  Adapter Trimming: DADA2  Quality-filtering: DADA2 plugin in QIIME2 v2019.17  Denoising: DADA2 plugin in QIIME2 v2019.17  Read merging: DADA2  Dereplication: DADA2  Chimera removal: DADA2 Clustering: ASVs were generated using DADA2 Phylogenetic tree: phylogeny plugin in QIIME v2019.17 Taxonomic assignment: feature classifier plugin in QIIME2 v2019.17 and the SILVA v132 V4 classifier (99% identity)  Alpha diversity: Shannon, Simpson, Observed ASVs, Faith's PD  Beta diversity: Bray-Curtis Dissimilarity and Unifrac (unweighted and weighted) distances using Phyloseq v1.28.0 (phyloseq:distance function), PCA analysis  Relative abundance: Differential abundance: ASV tables were agglomerated at multiple taxonomic levels (phylum to genus); LEfSe; Other analysis- MetaCyc pathways output from PICRUSt2 (Data S2) |
| Maes et al^90^ | Fecal sample | Fresh; sterile tubes with 2 mL DNA/RNA Shield™ (Zymo Research); stored at −20 °C until use | ZymoBIOMICS™ DNA Miniprep Kit (ZYMO Research, USA) | Analytical method: 16S rRNA gene amplicon sequencing, full length Primers: Jitvaropas et al., 2022: Fwd 5'-TTTCTGTTGGTGCTGATATTGCAGRGTTYGATYMTGGCTCAG-3'; Rev 5'-ACTTGCCTGTCGCTCTATCTTCCGGYTACCTTGTTACGACTT-3' Sequencing platform: ONT MinION Mk1C Flowcell: R10.4 Basecalling: Guppy basecaller v6.0.7 (super accuracy model, min acceptable quality scores Q > 10) Quality control: MinIONQC Demultiplexing; Adapter trimming: Porechop v0.2.4  Read merging: Not applicable  Dereplication: Not specified  Chimera removal: DADA2  Clustering; Polishing: NanoCLUST Taxonomic assignment: NanoCLUST trained on the RDP database  Rarefaction: 2,000 reads per sample  Alpha diversity: Chao1, Shannon  Beta diversity: Bray-Curtis Dissimilarity  Relative abundance: Differential abundance: Normalized taxonomic data. Enterotype analysis; MicrobiomeAnalyst, LEfSe (*p* < 0.05, LDA scores > 2), ILR (Isometric log-ratio) transformation + Box–Cox applied at phylum, genus, species levels |
| Maes et al^91^ | Fecal sample | Fresh; sterile tubes with 2 mL DNA/RNA Shield™ (Zymo Research); stored at −20 °C until use | ZymoBIOMICS™ DNA Miniprep Kit (ZYMO Research, USA) | Analytical method: 16S rRNA gene sequencing, full length Primers: Jitvaropas et al., 2022: Fwd 5'-TTTCTGTTGGTGCTGATATTGCAGRGTTYGATYMTGGCTCAG-3'; Rev 5'-ACTTGCCTGTCGCTCTATCTTCCGGYTACCTTGTTACGACTT-3' Sequencing platform: ONT MinION Mk1C Flowcell: R10.4 Basecalling: Guppy basecaller v6.0.7 (super accuracy model, min acceptable quality scores Q > 10) Quality control: MinIONQC Demultiplexing; Adapter trimming: Porechop v0.2.4  Read merging: Not applicable  Dereplication: Not specified  Chimera removal: DADA2  Clustering; Polishing: NanoCLUST Taxonomic assignment: NanoCLUST trained on the RDP database  Rarefaction: 2,000 reads per sample  Alpha diversity: Chao1, Shannon  Beta diversity: Bray-Curtis Dissimilarity  Relative abundance: Differential abundance: Normalized taxonomic data. MicrobiomeAnalyst, LEfSe (*p* < 0.05, LDA scores > 2), ILR (Isometric log-ratio) transformation + Box–Cox applied at phylum, genus, species levels |
| Malan-Muller et al^57^ | Fecal sample | PSP Spin Stool DNA Plus Kit (STRATEC); transport time and storage temperature not specified | PSP Spin Stool DNA Plus Kit (STRATEC Molecular, Birkenfeld, Germany) | Analytical method: 16S rRNA gene amplicon sequencing, V3-V4 Primers: Caporaso et al., 2019: 515F (5'-GTGCCAGCMGCCGCGGTAA-3'); 806R (5'-GGACTACHVGGGTWTCTAAT-3') Sequencing platform: Illumina MiSeq V2 Sequence reads: paired-end 2x 300 bp  Demultiplexing: bcl2fastq (ver. 2.17.1.14)  Adapter Trimming: DADA2 Quality control: FastQC and MultiQC Quality filtering: DADA2 v1.12.1 in R (default parameters: error threshold = 2; fwd/rev primer removal) Dereplication; Denoising: DADA2 v1.12.1in R (default parameters) Chimera removal: ASVs constructed. DADA2 v1.12.1 in R (default parameters) Taxonomic assignment: RDP Classifier (local copy, Train Set 16, database v11.5)  Alpha diversity: Simpson index  Beta diversity: Aitchison distances visualized with MDS  Relative abundance: Differential abundance: Normalized from taxonomic feature table. Data agglomerated to genus and phylum levels. CLR transformation, Metadata associations tested via capscale function in vegan (R), RandomForests (v4.6.14) package. Wilcoxon rank-sum test (2-level categorical), Spearman’s correlation (continuous), GLMs on CLR-transformed data adjusting for covariates (age, gender, BMI, GI diseases), Multiple testing corrected via Benjamini–Hochberg FDR (significance: *q* ≤ 0.1) |
| Malan-Muller et al^58^ | Fecal sample | PSP Spin Stool DNA Plus Kit (STRATEC); transport time and storage temperature not specified | PSP Spin Stool DNA Plus Kit (STRATEC Molecular, Birkenfeld, Germany) | Analytical method: 16S rRNA gene sequencing, V3-V4 Primers: 341F (5’- CCTACGGGNGGCWGCAG-3’); 805R (5'-GACTACHVGGGTATCTAATCC-3') Sequencing platform: Illumina MiSeq Sequence reads: paired-end 2x 300 bp  Demultiplexing: Illumina MiSeq® standard output  Adapter Trimming: DADA2 Quality control: FastQC and MultiQC Quality filtering: DADA2 v1.12.1 in R (default parameters: error threshold = 2; fwd/rev primer removal) Dereplication; Denoising: DADA2 v1.12.1 in R (default parameters) Chimera removal: DADA2 v1.12.1 in R (default parameters) Taxonomic assignment: RDP Classifier (local copy, Train Set 18, database v11.5)  Alpha diversity: Simpson’s index  Beta diversity: Aitchison distances visualized with MDS  Relative abundance: Differential abundance: Taxon’s read count by the total reads per sample, then applying CLR transformation with zero imputation for compositional data analysis. Other analysis: Wilcoxon rank-sum tests, PERMANOVA, Spearman correlation; adjusted using GLMs and FDR correction (*q* ≤ 0.1) |
| Mason et al^59^ | Fecal sample | Fresh; stored immediately in home freezers; transferred to the lab; stored at −80 °C until extraction; transport time not specified | Custom protocol based on Goodman et al.,^103^ | Analytical method: 16S rRNA gene amplicon sequencing, V4 Primers: Not specified Sequencing platform: Roche 454 Titanium  Sequence reads: Not specified  Adapter Trimming: Not specified Quality filtering: performed but specifications not provided Denoising: QIIME (version not specified) Clustering: UCLUST (97% similarity threshold) Taxonomic assignment: SILVA database (software not specified)  Alpha diversity: Shannon index Beta diversity: weighted UniFrac  Relative abundance: Differential abundance: Computed as the proportion of each bacterial taxon per sample and analysed using regression models and FDR-corrected tests to assess associations with clinical variables; Other analysis: PERMANOVA, FDR-adjusted using the Benjamini-Hochberg method |
| Naseribafrouei et al^92^ | Fecal sample | Fresh; frozen at −20 °C at home; transported to centralized −70 °C storage; hospitalized patients’ samples were taken directly to the centralized freezer | Mag™ Mini Kit (LGC, Middlesex, UK) | Analytical method: 16S rRNA gene amplicon sequencing Primers: Not specified Sequencing platform: Illumina MiSeq  Sequence reads: paired-end 2x 250 bp Demultiplexing; Quality filtering: QIIME2. Reads with average Phred score > 31 included (error rate < 0.001). Sequences with < 2000 or < 3000 reads per parallel were excluded from analysis  Denoising: QIIME v1.6.0  Clustering: QIIME v1.6.0 with closed-reference OTU picking using modified uclust parameters at 1% dissimilarity  Alpha diversity: Shannon index Beta diversity: PCA, PLS-DA  Taxonomic assignment: RDP Classifier  Relative abundance: Differential abundance: Assessed by associating bacterial group quantifications with diagnostic categories using logistic and multiple regression models; tested for differences using FDR-corrected permutation testing |
| Ritchie et al^60^ | Fecal sample | Fresh; Sarstedt containers with 5 mL RNAlater®, immediately frozen; returned on ice; stored at −80 °C | Not specified | Analytical method: 16S rRNA gene amplicon sequencing, V3-V4 Primers: F (5'-TCGTCGGCAGCGTCAGATGTGTATAAGAGACAGCCTACGGGNGGCWGC-3’); R (5'-GTCTCGTGGGCTCGGAGATGTGTATAAGAGACAGGACTACHVGGGTATCTAATCC-3'), derived from Klindworth et al., 2013 Sequencing platform: Illumina MiSeq V3 Sequence reads: paired-end 2x 300 bp Demultiplexing; FASTQ file generation: The Metagenomics Workflow (Illumina, 2014; min seq. length 1250 bp, max 50 wobble bases M, R, W, S, Y, K, V, H, D, B, and N)  Adapter Trimming: Not specified  Quality-filtering: Excluded: reads < 1250 bp, > 50 wobble bases, or unclassified at genus/species level  Read merging: Done by overlapping ends of paired-end reads (MiSeq protocol)  Dereplication: Not specified  Chimera removal: Not specified  Clustering: OTU assignment through MiSeq pipeline and Calypso; max 3000 taxa filtered by mean  Taxonomic assignment: Illumina-proprietary classification algorithm (ClassifyReads) trained on the Illumina-curated version of the Greengenes taxonomy database 13.5 (filter entries with no classification for genus or species).  Rarefaction analysis: 86,378 reads per sample Alpha diversity: Shannon, Simpson, ACE, Chao1, Pielou's evenness  Beta diversity: Bray–Curtis dissimilarity and weighted UniFrac distance, PCOA, PERMANOVA  Relative abundance: Differential abundance: Total sum normalization (TSS) followed by centered log-ratio (CLR) transformation. Differential abundance was assessed using DESeq2 with FDR correction, and correlations with mental health symptoms were evaluated using Spearman's rho and visualized via microViz |
| Rong et al^93^ | Fecal sample | Fresh; stored at −80 °C; Transport time not specified | StoolGen DNA kit (CWBiotech Co., Beijing, China) | Analytical method: Shotgun whole genome sequencing  Sequencing platform: Illumina HiSeq2500 Sequence reads: paired-end 2x 150 bp  Taxonomic assignment (profiling): Not specified Metabolic function analyses: the metagenomic catalogue was annotated to the KEGG orthologies (KO) database. PCA was used to visualize the differences of identified KOs among the three groups  Alpha diversity metrics: Chao1, Shannon, and Inverse Simpson indices using VEGAN'S diversity function Beta diversity: Bray-Curtis dissimilarity Relative abundance: Differential abundance: Gini coefficient; Wilcoxon rank-sum tests with FDR correction (*p* < 0.05); LEfSe (LDA > 2.5) |
| Tsai et al^43^ | Fecal sample | Fresh; RNA stabilizing reagent (RNALater); stored at −80 °C | Bead-beating method | Analytical method: 16S rRNA gene amplicon sequencing, V3-V4 Primers: 341F (CCTACGGGNGGCWGCAG); 805R (GACTACHVGGGTATCTAATCC)  Sequencing platform: Illumina MiSeq  Sequence reads: Not specified  Demultiplexing; QIIME2 Adapter trimming: q2-cutadapt in QIIME2 Quality filtering: DADA2 plugin in QIIME2 (q2-dada2 command) Denoising: DADA2 plugin in QIIME2 (q2-dada2 command) Phylogenetic tree: MAFFT and fasttree2 in QIIME2 (q2-phylogeny command) Taxonomic assignment: Naïve Bayes taxonomy classifier in QIIME2 (q2-feature-classifier classify-sklearn command) trained on the MetaSquare, Silva, Greengenes, RDP, HOMD, and Ezbiocloud databases  Rarefaction: performed but no specifications provided Alpha diversity: Shannon, Faith's PD Beta diversity: unweighted UniFrac distance, PCoA  Relative abundance: Differential abundance: LEfSe (alpha = 0.05, LDA scores > 2) |
| Wang et al^61^ | Fecal sample | Fresh; collected at baseline, week 8, and week 12; sent to the research assistant the same day, and immediately frozen at −80 °C until analysis | TIANamp Stool DNA Kit (TIANGEN Biotech Co. Ltd., Beijing, China) | Analytical method: 16S rRNA gene amplicon sequencing, V3-V4 Primers: 341F (5′-CCTAYGGGRBGCASCAG-3'); 806R (5'-GGACTACNNGGGTATCTAAT-3') Sequencing platform: Illumina MiSeq Sequence reads: paired-end 2x 300 bp  Demultiplexing: QIIME2  Adapter Trimming: cutadapt (via q2-cutadapt) Quality filtering: QIIME2 v2019.7  Dereplication; DADA2  Chimera removal: DADA2 Taxonomic assignment: Naïve Bayes classifier (q2-feature-classifier classify-sklearn) using MetaSquare, Silva, Greengenes, RDP, HOMD, and Ezbiocloud databases (plus manually added novel species)  Rarefaction: feature-table rarefaction using QIIME2 v2019.7 Alpha diversity: ACE, Simpson_E, Chao1, Fisher Beta diversity: Bray-Curtis dissimilarity, Generalized UniFrac distance, Jaccard dissimilarity, Unweighted and (normalized) Weighted UniFrac, PCoA  Relative abundance: Differential abundance: LEfSe (alpha = 0.05, LDA scores > 2). Other analysis: Mann-Whitney *U* test (*p* value 0.05 two-sided), PICRUSt2 |
| Yang et al^94^ | Fecal sample | Not specified | E.Z.N.A. Soil DNA Kit (Omega Bio-tek, Norcross, GA, USA) | Analytical method: Shotgun whole genome sequencing  Sequencing platform: Illumina NovaSeq Assembly (single strains): MetaWRAP (completeness > 75; contamination < 5)  Clustering: MetaPhlAn3 for profiling; MetaWRAP used to assemble individual strains (filtered for completeness > 75% and contamination < 5%) Taxonomic assignment: MetaPhlAn3  Alpha diversity metrics: Chao, Shannon and Inverted Simpson indices using Vegan in R and visualized using fossil in R  Beta diversity metrics: PCoA; PERMANOVA  Relative abundance: Differential abundance: LEfSe (LDA scores > 2.5). Other analysis: Wilcoxon rank sum test (false discovery rate, < 0.05), Diamond for KEGG annotation (e value cutoff of 1 × 10^−5^). |
| Ye et al^95^ | Fecal sample | Fresh; collected using disposable feces collectors; frozen at −80 °C | PowerSoil® DNA Isolation Kit (MO BIO Laboratories, Carlsbad, CA, USA) | Analytical method: 16S rRNA gene amplicon sequencing, V3-V4 Primers: F (5′-ACTCCTACGGGAGGCAGCA-3'); R (5'-GGACTACHVGGGTWTCTAAT-3') Sequencing platform: Illumina HiSeq Sequence reads: paired-end 2x 250 bp.  Demultiplexing: Not specified  Adapter Trimming: Trimmomatic v0.33 Quality filtering: Trimmomatic v.0.33 Read merging: FLASH (v 1.2.7)  Dereplication; Not specified Chimera removal: UCHIME v4.2 Clustering: UPARSE (97% similarity cut-off) Taxonomic assignment: RDP classifier  Alpha diversity: Chao1, Shannon  Beta diversity: unweighted UniFrac distance visualized with PCoA Relative abundance: Differential abundance: LEfSe (alpha = 0.05, LDA scores > 3.5) |
| Yu et al^96^ | Fecal sample | Fresh; collected at the second visit using home kits in the non-menstrual morning and stored immediately at −80 °C | Cetyltrimethylammonium bromide method | Analytical method: Shotgun whole genome sequencing  Sequencing platform: Illumina HiSeq 2500  Sequence reads: paired-end 2x 150 bp Shotgun Quality-filtering: Trimmomatic (ILLUMINACLIP:adapters path:2:30:10 SLIDINGWINDOW:4:20 MINLEN:50) Host read removal: Bowtie2 (parameter -very-sensitive) in KneadData v0.7.04 Quality control: FASTQC v0.11.9 Taxonomic profiling: KraKen2 v2.0.7 beta and Bracken V2.0 trained on a local database based on NCBI nucleotide and RefSeq wholegenome datasets  Alpha diversity: Chao1, Shannon, Simpson  Beta diversity: indices not specified  Functional analysis: GO, KEGG, and MetaCyc using HUMAnN3  Relative abundance: Differential abundance: LEfSe (LDA score > 2) |
| Yirmiya et al^97^ | Fecal sample | Fresh; collected during home visits; kit, transport time, and freezing temperature not specified. | Not specified | Analytical method: 16S rRNA gene amplicon sequencing, V4 Primers: Caporaso et al. 2012. From suppl. mat. 515F (5′-AATGATACGGCGACCACCGAGATCTACACGCT-3'); 806R (5'-TATGGTAATTGTGTGYCAGCMGCCGCGGTAA-3') Sequencing platform: Illumina MiSeq Sequence reads: Not specified Demultiplexing; Quality filtering: QIIME2 Denoising: DADA2 plugin in QIIME2 Taxonomic assignment: Not specified  Rarefaction: 8,000 sequences per sample Alpha diversity: Shannon index. (Kruskal–Wallis H test and Mann-Whitney *U* test)  Beta diversity: Aitchison distance (Euclidean distance over CLR-transformed ASVs)  Relative abundance: Differential abundance: Performed at genus level with log normalization; XGBOOST. Other analysis - PICRUSt2; Kruskal–Wallis, Mann–Whitney *U* test |
| Yuan et al^62^ | Fecal sample | Fecal biopsy from large intestine; collection and storage details not specified | AllPrep DNA/RNA Mini Kit (Qiagen, Hilden, Germany) | Analytical method: 16S rRNA gene amplicon sequencing, V3-V4 Primers: 338F (ACTCCTACGGGAGGCAGCAG); 806R (GGACTACHVGGGTWTCTAAT) Sequencing platform: Illumina MiSeq Sequence reads: Not specified  Demultiplexing; Quality-filtering: Illumina Analysis Pipeline v2.6 (min sequence length 200 bp, min quality value = 20, no ambiguous basecalls)  Clustering: ASV-based approach via DADA2 Taxonomic assignment: RDP classifier trained on the SILVA database v128  Alpha diversity: Shannon, PD Whole Tree  Beta diversity: unweighted UniFrac  Relative abundances: Differential abundance: OTUs ≥ 0.5% other analysis - PICRUSt v1.1.4; Correlation analysis (SparCC, Spearman); Bonferroni and FDR adjustments |
| Zeamer et al^49^ | Fecal sample | OMNIgene•GUT kits (OMR-200); heat-inactivated at 65–70 °C for 1 h; stored at −80 °C; transport time not specified | QIAGEN DNeasy PowerSoil Pro Kits (QIAGEN, catalog no. 47016) | Analytical method: Shotgun whole genome sequencing  Sequencing platform: Illumina NextSeq 500 Sequence reads: paired-end 2x 150 bp  Taxonomic assignment (profiling): Metaphlan3  Adapter Trimming & Quality Filtering**:** KneadData pipeline used for trimming and host contamination removal  Alpha diversity: Simpson and Shannon indices  Beta diversity: Not processed/specified  Relative abundance: Differential abundance: MetaPhlAn3; Linear Mixed Models **(**FDR-corrected *p* values ≤ 0.05; ANOVA and sum of squares calculated); Mixed-Effect Random Forest. Other analysis- HUMAnN3; Spearman correlation |
| Zhang et al^63^ | Fecal sample | Not specified | E.Z.N.A. Soil DNA kit (Omega Bio-Tek, Norcross, GA, USA). | Analytical method: 16S rRNA gene amplicon sequencing, V3-V4 Primers: 338F (5′-ACTCCTACGGGAG-GCAGCAG-3'); 806R (5'-GGACTACHVGGGTWTCTAAT-3') Sequencing platform: Illumina MiSeq Sequence reads: paired-end 2x 250 bp  Demultiplexing; Quality filtering: QIIME (version not specified) Clustering: UPARSE (97% similarity cutoff) Chimera removal: UCHIME Taxonomic assignment: RDP classifier trained on the SILVA database v132 (confidence threshold 70%)  Alpha diversity: Chao1, Shannon Beta diversity: Bray–Curtis dissimilarity visualized with PCoA and weighted UniFrac. ANOSIM  Relative abundance: Differential abundance: OTUs (≥ 0.1%) LEfSe, LDA ≥ 2**,** *p* < 0.05 |
| Zhang et al^45^ | Fecal sample | Fresh; RNA stabilization solution (Tiny-Gen, China); ≥ 1 g within 2 days post-admission; stored at −80 °C | Not specified | Analytical method: 16S rRNA gene amplicon sequencing, V4-V5 Primers: 515F (5′-GTGCCAGCMGCCGCGGTAA-3'); 926R (5'-CCGTCAATTCMTTTGAGTTT-3'). Sequencing platform: Illumina NovaSeq6000 SP 500 Sequence reads: paired-end 2x 250 bp  Adapter Trimming: Trimmomatic v0.35 Quality-filtering: Trimmomatic (SLIDINGWINDOW:50:20 MINLEN:50) Read merging: FLASH v1.2.11 (default parameters) Quality-filtering (2): screen.seqs command in Mothur v1.33.3 (maxambig = 0, minlength = 200, maxlength = 485, maxhomop =8) Clustering: UPARSE in USEARCH version v8.1.1756 (97% similarity cutoff) and R (v3.6.0) Taxonomic assignment: Not specified  Alpha diversity: Shannon, Simpson, Evenness  Beta diversity : UniFrac (unweighted and weighted) distances, visualized with PCoA, Bray–Curtis and Jaccard dissimilarity visualized by R as UniFrac analysis  Relative abundance: Differential abundance: Taxa with abundance > 0.01% were retained; ANOSIM; Wilcoxon signed-rank test; LEfSe (LDA score ≥ 2.0 or ≤ −2.0, *p* < 0.05) |
| Zhang et al^46^ | Fecal sample | Fresh; RNA stabilization solution (Tiny-Gen, China); ≥ 1 g within 2 days post-admission; stored at −80 °C. | Not specified | Analytical method: 16S rRNA gene amplicon sequencing, V4-V5 Primers: 515F (5′-GTGCCAGCMGCCGCGGTAA-3'); 926R (5'-CCGTCAATTCMTTTGAGTTT-3') Sequencing platform: Illumina NovaSeq6000 SP 500 Sequence reads: paired-end 2x 250 bp Target  Demultiplexing: mothur/UPARSE  Adapter Trimming: Trimmomatic v0.35 Quality filtering: Using mothur v1.33.3, UPARSE (USEARCH v8.1.1756), and R (v3.6.0); filters: maxambig = 0, minlength = 200, maxlength = 485, maxhomop = 8 Read merging: FLASH v1.2.11 (default parameters)  Dereplication; Not specified Chimera removal: Not specified Clustering: UPARSE (97% similarity cut-off) Taxonomic assignment: Not specified  Alpha diversity: Shannon, Simpson, Evenness, Chao1, ACE  Beta diversity: Bray Curtis, Weighted Unifrac  Relative abundance: Differential abundance: Metastas |
| Zhang et al^64^ | Fecal sample | Fresh; collected on behavioral assessment day; frozen immediately; stored at −80 °C | PowerSoil DNA Isolation Kit (Qiagen, Germany) | Analytical method: 16S rRNA gene amplicon sequencing, V4 Primers: 515F (5′-GTGCCAGCMGCCGCGGTAA-3'); 806R (5'-GG ACTACHVGGGTWTCTAAT-3') Sequencing platform: Illumina MiSeq Sequence reads: paired-end 2x 250 bp Quality filtering: adaptors and low-quality and ambiguous bases were removed (software not specified) Clustering: UPARSE v7.1 (97% similarity cutoff) Chimera removal: UCHIME v4.2.40 through comparison with the Gold database Taxonomic assignment: RDP classifier v2.2 (confidence threshold 60%) and trained on the Greengenes database v201305 using QIIME v1.8.0  Alpha diversity: Shannon, Simpson  Beta diversity: Aitchison distance and visualized with PCA Relative abundance: Differential abundance: Random Forest Analysis; Other analysis - Kruskal-Wallis test (non-parametric); CLR-transformed prior to statistical analysis; Benjamini–Hochberg correction applied; Spearman correlation, *p* < 0.05 |
| Zhao et al^98^ | Fecal sample | Fresh; collected same day as symptom assessment; frozen immediately; stored at −80 °C | Not specified | Analytical method: Shotgun whole genome sequencing  Sequencing platform: Illumina NovaSeq 6000  Sequence reads: paired-end 2x 150 bp  Sequence parsing: Readfq v8  Assembly: SOAPdenovo v2.04  ORF prediction: MetaGeneMark v2.10 Gene clustering: CD-HIT v.4.5.8  Gene annotation: LCA algorithm in DIAMOND v0.9.9 using the NCBI NR database v2018-01-02  Taxonomic assignment (profiling): DIAMOND (V0.9.9)  Alpha diversity: Not processed/specified  Beta diversity: PCA  Relative Abundance: Differential abundance: Calculated at multiple taxonomic levels from LCA annotation and gene abundance table. Metastats and LEfSe (LDA scores > 2) Random Forest in R (pROC and randomForest packages, v. 2.15.3). Other analysis: KEGG (DIAMOND v0.9.9) |
| Zheng et al^99^ | Fecal sample | Fresh; tube with 2 mL preservative; frozen at −80 °C | QIAamp DNA Stool Mini Kit (QIAGEN) | Analytical method: 16S rRNA gene amplicon sequencing, V3-V4 Primers: 515F (5'-GTGCCAGCMGCCGCGGTAA-3'); 926R (5'-CCGTCAATTCMTTTGAGTTT-3') Sequencing platform: Illumina MiSeq Sequence reads: paired-end 2x 300 bp  Quality filtering: Trimmomatic v0.35 (SLIDING WINDOW: 50:20 MINLEN: 50)  Read merging: FLASH 1.2.11 (default parameters) Contig filtering: screen.seqs command in Mothur v1.33.3 (parameters, maxambig = 0, minlength = 200, maxlength = 580, maxhomop = 8)  Clustering: UPARSE in USEARCH v8.1.1756 (97% sequence identity) Taxonomic assignment: Not specified  Alpha diversity: Chao1, ACE, Shannon, Simpson, Sobe Relative Abundance: Differential abundance: LEfSe |
| Zhong et al^100^ | Fecal sample | Not specified | PowerSoil® DNA Isolation Kit (MO BIO Laboratories, Carlsbad, CA, USA) | Analytical method: 16S rRNA gene amplicon sequencing, V4-V5 Primers: 515F (5′-GTGCCAGCMGCCGCGGTAA-3'); 926R (5'-CCGTCAATTCMTTTGAGTTT-3') Sequencing platform: Illumina MiSeq and NovaSeq Sequence reads: MiSeq: paired-end 2x 300bp; NovaSeq: paired-end 2x 250 bp  Demultiplexing: exact barcode matching, max two-nt mismatch in primer matching; no ambiguous basecalls  Adapter Trimming: Not specified Quality-filtering: FASTP v0.20.0 (min sequence length 50 bp, min quality value = 20 over 50-bp sliding window) Read merging: FLASH v1.2.7; min sequence overlap = 10 bp, max mismatch ratio of the overlap region = 0.2)  Chimera removal: UPARSE Clustering: UPARSE in USEARCH v7.1 (97% similarity cutoff) Taxonomic assignment: RDP classifier v2.2 (confidence threshold of 70%)  Alpha diversity: Shannon, Simpson  Beta diversity: PCoA  Relative Abundance: Differential abundance: Calculated at different taxonomic levels. Other analysis- OPLS-DA; Stepwise logistic regression (AIC-based model selection); ROC curve analysis (AUC) |
| Zhou et al^101^ | Fecal sample | Fresh; sterile plastic cup; immediately stored at −20 °C; transported and stored at −80 °C storage | PowerSoil® DNA Isolation Kit (MO BIO Laboratories, Carlsbad, CA, USA) | Analytical method: 16S rRNA gene amplicon sequencing, V4 Primers: 515F (5′-GTGYCAGCMGCCGCGGTAA-3'); 806R (5'-GGACTACNVGGGTWTCTAAT-3') Sequencing platform: Illumina MiSeq V3 Sequence reads: paired-end 2x 150 bp Demultiplexing: Quality-filtering: QIIME v2.0 Clustering: QIIME v2.0 (ASVs clustered at a 97% similarity threshold) Taxonomic assignment: QIIME v2.0 trained on the SILVA database Alpha diversity: Eveness, Observed species, Shannon, Faith-PD Beta diversity: UniFrac visualized using PCoA  Relative Abundance: Differential abundance: Taxa summaries computed and reformatted; taxonomic composition profiles analysed. LEfSe (alpha = 0.05, LDA scores > 2). Other analysis - Kruskal–Wallis test (α = 0.05); Wilcoxon rank-sum test; Spearman’s Rho test |
| Zhou et al^65^ | Fecal sample | Fresh; immediately transferred to the laboratory; stored at −80 °C | Not specified | Analytical method. Target region: 16S rRNA gene amplicon sequencing, V3-V4 Primers: 338F; 806R (sequences not specified) Sequencing platform: Illumina MiSeq Sequence reads: paired-end 2x 300 bp  Demultiplexing; Quality filtering: QIIME v1.9.0 (default parameters) Chimera removal: ChimeraSlayer Clustering: 97% similarity threshold and minimum occurrence 0.005% of the total seqs; software not specified Taxonomic assignment: Not specified  Alpha diversity: Observed species, ACE, Chao1, Shannon, Simpson   Beta diversity: weighted UniFrac visualized with PCoA  Relative Abundance: Differential abundance: Genera with relative abundance ≥ 0.01 were considered major genera. LEfSe (LDA scores > 2). Other analysis - Kruskal–Wallis test |
| Zhou et al^102^ | Fecal sample | Fresh; at baseline in sterile cups, frozen at −80 °C immediately after defecation | PowerSoil® DNA Isolation Kit (MO BIO Laboratories, Carlsbad, CA, USA) | Analytical method: 16S rRNA gene amplicon sequencing, V4 Primers: 515F (5′-GTGYCAGCMGCCGCGGTAA-3'); 806R (5'-GGACTACNVGGGTWTCTAAT-3') Sequencing platform: Illumina MiSeq V3 Sequence reads: paired-end 2x 150 bp  Demultiplexing; Quality filtering: QIIME2.0  Clustering: QIIME2.0 (ASVs, 97% similarity threshold) Taxonomic assignment: QIIME2.0 trained on the SILVA database  Alpha diversity: Evenness, Observed species, Shannon, Faith-PD  Beta diversity: Bray-Curtis dissimilarity and unweighted UniFrac distance visualized with PCoA  Relative Abundance: Differential abundance: LEfSe (*p* < 0.05, LDA cut-off 2.0). Other analysis: Wilcoxon rank sum test |
| Zhu et al.,^48^ | Fecal sample | Fresh; smeared on FOBT cards before endoscopy; sealed, stored at −80 °C, and shipped on dry ice | PowerSoil® DNA Isolation Kit (MO BIO Laboratories, Carlsbad, CA, USA) | Analytical method: 16S rRNA gene amplicon sequencing, V4.  Primers: 515F (5′-GTGYCAGCMGCCGCGGTAA-3′); 806R (5′-GGACTACNVGGGTWTCTAAT-3′)  Sequencing platform: Illumina Miniseq  Sequence reads: paired-end 2x 300 bp  Adapter Trimming: Illumina MiniSeq Reporter Quality filtering: DADA2 plugin in QIIME2  Read merging: DADA2  Dereplication; DADA2 Clustering: ASVs, 97% similarity threshold  Taxonomic assignment: QIIME2 trained on the Greengenes database v13_8  Alpha diversity: Evenness, Shannon, Observed OTUs, Chao1  Beta diversity: Bray-Curtis dissimilarity, Jaccard distance, UniFrac (unweighted and weighted) distances visualized with PCoA  Relative Abundance: Differential abundance: Genera with relative abundance ≥ 0.01. LEfSe (LDA ≥ 2.032; *p* < 0.05) Other analysis: Wilcoxon rank-sum test; PERMANOVA |
| Zhu et al^103^ | Fecal sample | Not Specified | TIANamp DNA Kit (QIAGEN) | Analytical method: 16S rRNA gene amplicon sequencing, V4  Primers: 520F (5'-AYTGGGYDTAAAGNG-3'); 802R (5'-TACNVGGGTATCTAATCCT-3')  Sequencing platform: Illumina HiSeq2000  Sequence reads: paired (length not specified)  Quality-filtering: min quality score 20, no ambiguous basecalls, no improper primers; software not specified  Read merging: FLASH Chimera removal: chimeras were checked and eliminated during clustering; software not specified Clustering: UPARSE (97% similarity threshold) Taxonomic assignment: not specified  Alpha diversity: Shannon, Chao1  Beta diversity: unspecified metric visualized with PCoA  Relative Abundance: Differential abundance: categorized by taxonomic level (phylum to genus), annotation method not specified |
| Zu et al^104^ | Fecal sample | Fresh; middle portion; ice box; transferred to lab; packed in cryotubes, stored at −80 °C | E.Z.N.A.® soil DNA Kit (Omega Bio-tek, Norcross, GA, U.S.) | Analytical method. 16S rRNA gene amplicon sequencing, V4  Primers: 338F (5'-ACTCCTACGGGAGGCAGCAG-3'); 806R (5'-GGACTACHVGGGTWTCTAAT-3')  Sequencing platform: Illumina MiSeq PE300 platform/NovaSeq PE250 platform (Illumina, San Diego, USA) according to the standard protocols by Majorbio Bio-Pharm Technology Co. Ltd. (Shanghai, China)  Sequence reads: paired (length not specified)  Demultiplexing: In-house Perl script  Quality Filtering: Performed using Fastp v0.19.6 with trimming (average quality score threshold of 20 in 50-bp windows, reads < 50 bp and with Ns removed)  Clustering: UPARSE v7.1 at 97% similarity to define OTUs  Taxonomic Assignment: RDP Classifier v2.11 trained on the SILVA database, using 70% confidence threshold  Alpha diversity: Shannon, Simpson, Sobs, Chao1, ACE, Good's coverage Beta diversity: unspecified metric, PCA  Relative Abundance: Differential abundance: Categorized by taxonomic classification levels. Taxonomic comparisons between groups were conducted using multivariate statistics to identify differential taxa. Pearson correlation analysis was used to assess associations between top 30 bacterial genera and serum/urine metabolites |
| 16S rRNA = 16S ribosomal RiboNucleic Acid; ACE = Abundance-based Coverage Estimator; ANCOM = Analysis of Composition of Microbiomes; ANOSIM = Analysis of Similarities; ASV = Amplicon Sequence Variant; AUC = Area Under the Curve; DIAMOND = Double Index Alignment of Next-generation sequencing Data; FDR = False Discovery Rate; KEGG = Kyoto Encyclopedia of Genes and Genomes; LCA = Lowest Common Ancestor; LC-MS/MS = Liquid Chromatography–Tandem Mass Spectrometry; LDA = Linear Discriminant Analysis; LEfSe = Linear Discriminant Analysis Effect Size; MDS = Multidimensional Scaling; MEGAN5 = MEtaGenome ANalyzer version 5; MetaPhlAn3 = Metagenomic Phylogenetic Analysis version 3; NCBI NR = National Center for Biotechnology Information Non-Redundant Protein Sequence; NMDS = Non-metric Multidimensional Scaling; OPLS-DA = Orthogonal Partial Least Squares Discriminant Analysis; OTU = Operational Taxonomic Unit; PCA = Principal Component Analysis; PCoA = Principal Coordinates Analysis; PERMANOVA = Permutational Multivariate Analysis of Variance; PD = Phylogenetic Diversity; PICRUSt = Phylogenetic Investigation of Communities by Reconstruction of Unobserved States; PGM = Personal Genome Machine; QIIME = Quantitative Insights Into Microbial Ecology; RDP = Ribosomal Database Project; ROC = Receiver Operating Characteristic; SDS = Sodium Dodecyl Sulfate; UniFrac = Unique Fraction Metric; WCNA = Weighted Correlation Network Analysis. | | | | |
